# *Candida*–*Acinetobacter*–Pseudomonas Interaction Modelled within 286 ICU Infection Prevention Studies

**DOI:** 10.3390/jof6040252

**Published:** 2020-10-27

**Authors:** James C. Hurley

**Affiliations:** Rural Health Academic Center, Melbourne Medical School, University of Melbourne, Ballarat 3350, Australia; jamesh@bhs.org.au

**Keywords:** *Candida*, bacteraemia, *Acinetobacter*, *Pseudomonas*, study design, intensive care, mechanical ventilation, generalized structural equation model

## Abstract

Background: Whether *Candida* interacts to enhance the invasive potential of *Acinetobacter* and *Pseudomonas* bacteria cannot be resolved within individual studies. There are several anti-septic, antibiotic, anti-fungal, and non-decontamination-based interventions to prevent ICU acquired infection. These effective prevention interventions would be expected to variably impact *Candida* colonization. The collective observations within control and intervention groups from numerous ICU infection prevention studies simulates a multi-centre natural experiment with which to evaluate *Candida*, *Acinetobacter* and *Pseudomonas* interaction (CAPI). Methods: Eight Candidate-generalized structural equation models (GSEM), with *Candida*, *Pseudomonas* and *Acinetobacter* colonization as latent variables, were confronted with blood culture and respiratory tract isolate data derived from >400 groups derived from 286 infection prevention studies. Results: Introducing an interaction term between *Candida* colonization and each of *Pseudomonas* and *Acinetobacter* colonization improved model fit in each case. The size of the coefficients (and 95% confidence intervals) for these interaction terms in the optimal *Pseudomonas* (+0.33; 0.22 to 0.45) and *Acinetobacter* models (+0.32; 0.01 to 0.5) were similar to each other and similar in magnitude, but contrary in direction, to the coefficient for exposure to topical antibiotic prophylaxis (TAP) on *Pseudomonas* colonization (−0.45; −0.71 to −0.2). The coefficient for exposure to topical antibiotic prophylaxis on *Acinetobacter* colonization was not significant. Conclusions: GSEM modelling of published ICU infection prevention data supports the CAPI concept. The CAPI model could account for some paradoxically high *Acinetobacter* and *Pseudomonas* infection incidences, most apparent among the concurrent control groups of TAP studies.

## 1. Introduction

*Candida* colonization at the respiratory tract and elsewhere is associated with poor outcomes among high-risk ICU patients [1]. The reason for this association remains unclear as *Candida* itself is a rare cause of ventilator-associated pneumonia (VAP) and candidemia occurs infrequently in the ICU [2,3]. Several preclinical studies have implicated a potential interaction between *Candida* colonization and invasive infection with Gram-negative bacilli including *Pseudomonas* and *Acinetobacter* bacteria [4].

Defining the clinical relevance of the postulated *Candida*–*Acinetobacter*–*Pseudomonas* interaction (CAPI) is unlikely to be achieved within the constraints of a single centre study as there are several obstacles. Moreover, measuring colonization and quantifying the impact of the various interventions on it is not simple [5]. However, the collective observations within control and intervention groups from numerous ICU infection prevention studies simulates a multi-centre natural experiment of Candida colonization subject to various exposures and provides an opportunity to evaluate CAPI.

The prevention of VAP and other ICU acquired infections is of great interest and various anti-septic, antibiotic, anti-fungal, or non-decontamination-based interventions have been studied [6,7,8,9,10,11,12,13,14,15,16,17,18,19,20,21,22,23,24,25,26]. These methods variably target colonization with Gram-negative bacteria such as *Pseudomonas* and *Acinetobacter* bacteria and also *Candida* [26,27]. Topical antibiotic prophylaxis (TAP)-based methods appear to be the most effective for the overall prevention of VAP and bacteraemia among ICU patients [27]. Surprisingly, the incidences of infection with *Candida* [28,29] and likewise incidences of both *Acinetobacter* [30] and *Pseudomonas* bacteraemia [31] are unusually high among studies of methods using TAP. This is both paradoxical, as topical amphotericin, polymyxin and aminoglycosides are common TAP constituents, and unexplained.

The objective here is to develop candidate generalized structural equation models (GSEM) founded on CAPI concepts and then confront these models using group-level infection data from published studies of ICU patient groups being subject to various group-level exposures.

## 2. Materials and Methods 

Being an analysis of published work, ethics committee review of this study was not required.

### 2.1. Study Selection and Decant of Groups 

**Figure 1:**
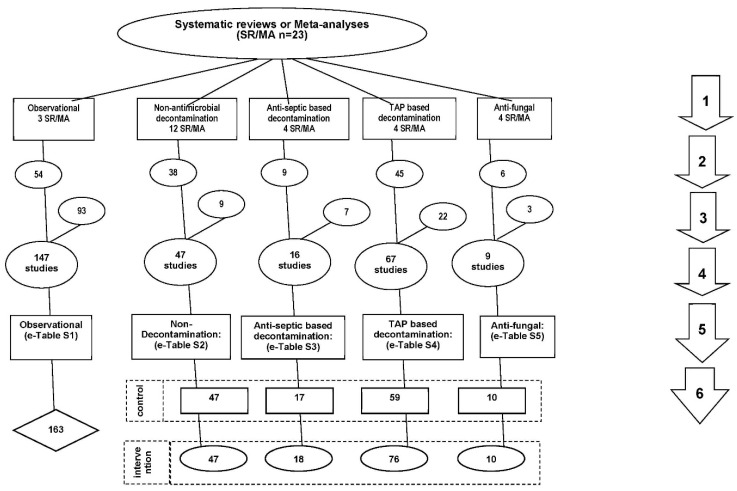
Search method, screening criteria and resulting classification of eligible studies and subsequent decant of component groups. The four numbered arrows are as follows; An electronic search for systematic reviews or meta-analysis (SR/MA) containing potentially eligible studies using search terms; “ventilator associated pneumonia”, “mechanical ventilation”, “intensive care unit”, each combined with either “meta-analysis” or “systematic review” up to December 2018. The systematic reviews were then searched for studies of patient populations requiring prolonged (>24 h) ICU admission; The studies were triaged from the systematic reviews into one of five categories; studies in which there was no intervention (observational studies), studies of various non-decontamination methods such as methods delivered either via the gastric route, the airway route or via the oral care route, studies of anti-septic methods, studies with a TAP-based (in any formulation) intervention, and studies of antifungal prophylaxis as a single agent. All studies were reviewed for potentially eligible studies and screened against inclusion and exclusion criteria. Any duplicate or ineligible studies were removed and studies identified outside of systematic reviews were included. The component groups were decanted from each study being control (rectangles), intervention (ovals) and observation (diamond) groups. Note: the total numbers do not tally as some systematic reviews provided studies in more than one category and some studies provided groups in more than one category and some studies have unequal numbers of control and interventions groups. Abbreviations; TAP = Topical antibiotic prophylaxis; ICU = Intensive Care Unit; SR/MA = systematic reviews or meta-analysis.

The literature search and study decant used here is as in Figure 1. Twenty-three systematic reviews and meta-analyses were used as the starting point as these provided evidence base of the interventions of interest [1,5,6,7,8,9,10,11,12,13,14,15,16,17,18,19,20,21,22,23,24,25,26]. A snowballing search strategy [32] using the “Related articles” function within Google Scholar was undertaken for additional studies not identified within key systematic reviews. The key inclusion and exclusion criteria described previously [27], being a patient group requiring prolonged (>24 h) ICU stay with group-level data for any of *Pseudomonas* or *Acinetobacter* infection data available, was expanded to include *Candida* infection data and studies of single anti-fungal prophylaxis [28,29]. All eligible studies were then collated, and any duplicate studies were removed.

In the GSEM modelling, the *Candida*, *Pseudomonas* and *Acinetobacter* infection data serve as the measurement components, the group-level exposure parameters serve as the structural components and colonization with *Candida*, *Pseudomonas* and *Acinetobacter*, each represented as latent variables, link the structural and measurement components.

### 2.2. Measurement Components 

The incidences of *Pseudomonas* and *Acinetobacter* VAP as well as the incidences of *Pseudomonas* and *Acinetobacter* bacteraemia were extracted. As *Candida* is generally not thought to be a cause of VAP, the count of *Candida* as a respiratory tract (RT *Candida*) isolate among patients with suspected VAP was recorded along with counts of candidemia. Counts for all subspecies of *Candida*, *Pseudomonas* and *Acinetobacter* were included. These were each expressed as a proportion using the number of patients with a prolonged (>24 h) stay in the ICU as the denominator.

### 2.3. Structural Components 

The following data were extracted and used to form the structural components of the models; year of study publication, whether the majority of the group were trauma patients, whether more than 90% of patients of the group received more than 24 h of MV, and whether the mean (or median) length of ICU stay for the group was less than five, five to ten days, or more than ten days. In the extraction of MV percentages, if this was not stated for any group, a percentage less than 90% was assumed. In the extraction of ICU length of stay (ICU-LOS) data from the studies, surrogate measures including mean (or median) length of mechanical ventilation were taken if the length of ICU-LOS was not available in order to generate broad categories of ICU stay of less than 5 days, 5 to 10 days and more than 10 days.

Additionally, the presence of any of the following group wide risk factors for candidemia and invasive *Candida* infection were noted including liver transplantation or liver failure, use of parenteral nutrition, surgery for intestinal perforation, pancreatitis and being colonized with *Candida*, regardless of how *Candida* colonization was defined. An anti-septic exposure included use of agents such as chlorhexidine, povidone-iodine and iseganan. All anti-septic exposures were included regardless of whether the application was to the oropharynx, by tooth-brushing or by body-wash.

Topical antibiotic prophylaxis (TAP) is defined here as the group wide application of topical antibiotics to the oropharynx or stomach without regard to the specific antibiotic constituents. Protocolized parenteral antibiotic prophylaxis (PPAP) is the group wide use of any parenteral antibiotic used on a prophylactic basis. Group wide exposure to anti-fungal prophylaxis was identified whether this was as a single agent, or as part of a multi-agent decontamination regimen such as within selective digestive decontamination, without regard to the specific anti-fungal agent.

### 2.4. Structural Equation Modelling

Eight candidate GSEM models were developed with *Candida* colonization and either *Pseudomonas* (four models; models 1 to 4) or *Acinetobacter* colonization (four models; models 5 to 8) as the latent variables intermediary between the structural and measurement components. The models were constructed with (models 1, 2, 5 and 6) and without (models 3, 4, 7 and 8) the inclusion of studies with group mean length of ICU stay less than 5 days and with (models 2, 4, 6 and 8) and without (models 1, 3, 5 and 7) an interaction term between the *Candida* colonization and respectively the *Pseudomonas* and *Acinetobacter* colonization latent variables.

As the observations are clustered by study, in each model, a study identifier was used in order to generate a robust variance covariance matrix of the parameters of each coefficient estimate. The GSEM model with the lowest Akaike’s information criterion (AIC) score was selected as having parsimony and optimal fit from among the candidate models using the “GSEM” command in Stata [33].

### 2.5. Visual Benchmarking

Scatter plots of the *Candida*, *Pseudomonas* and *Acinetobacter* infection data were generated to facilitate a visual survey of the entire data as derived from the literature. To facilitate this visual survey, a benchmark for each outcome of interest was generated from the groups of the observational studies as described previously [27].

### 2.6. Availability of Data and Materials

All data generated or analysed during this study are included in this published article and its supplementary information files (see Electronic Supplementary Materials; ESM).

## 3. Results

### 3.1. Characteristics of the Studies

Of the 286 studies identified by the search, 135 were sourced from 23 systematic reviews (Table 1, ESM Tables S1–S5). Others were found during previous searches or by snowball sampling (ESM Figure S1). Most studies were published between 1990 and 2010 and most had a mean ICU-LOS exceeding ten days. A minority originated from either North American or trauma ICU’s. Twelve studies had more than one type of intervention group and eight studies had no control group. The majority of groups from studies of infection prevention interventions had less than 150 patients per group whereas most of the observational studies had more than 150 patients per group.

Of the 286 studies, there were 23 groups from 12 studies having a group mean ICU-LOS less than 5 days including the largest (>120,000 patients), which was a study of targeted versus universal decontamination versus standard care [34].

There was inequality in the numbers of infection prevention exposures. The majority of studies of anti-fungal exposure occurred within an exposure to combined TAP and anti-fungals in the context of a Selective Digestive Decontamination (SDD) regimen for which the antifungal used was topical amphotericin in 50 groups. The TAP exposures included either topical polymyxin or a topical aminoglycoside or both in every case except four intervention groups. PPAP, most commonly a cephalosporin, was used within ten control groups and 44 intervention groups of TAP studies. By contrast, anti-fungal prophylaxis used as a single agent (i.e., constituted without topical antibiotics) occurred in only nine groups and for most of these groups, the patient groups were selected on the basis of risk factors for invasive *Candida* infection.

### 3.2. Infection Data

**Figure 2:**
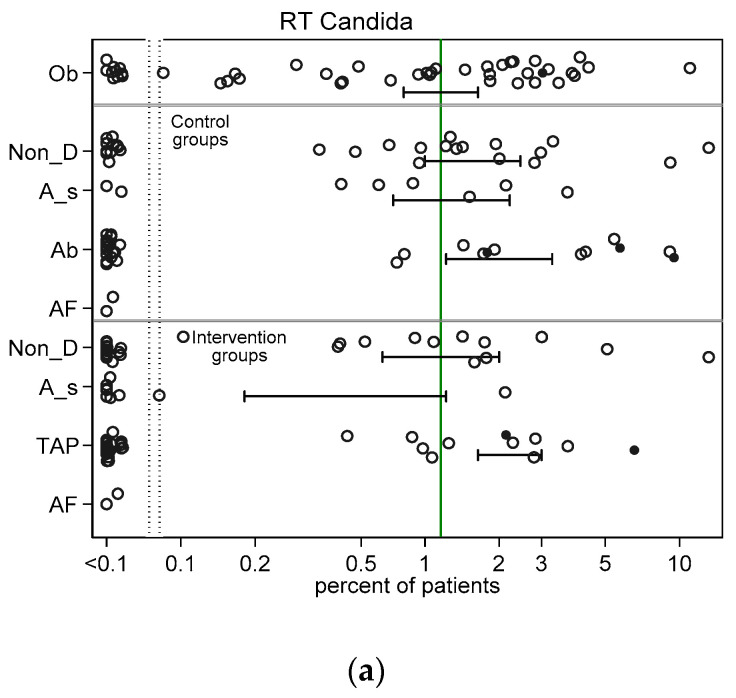

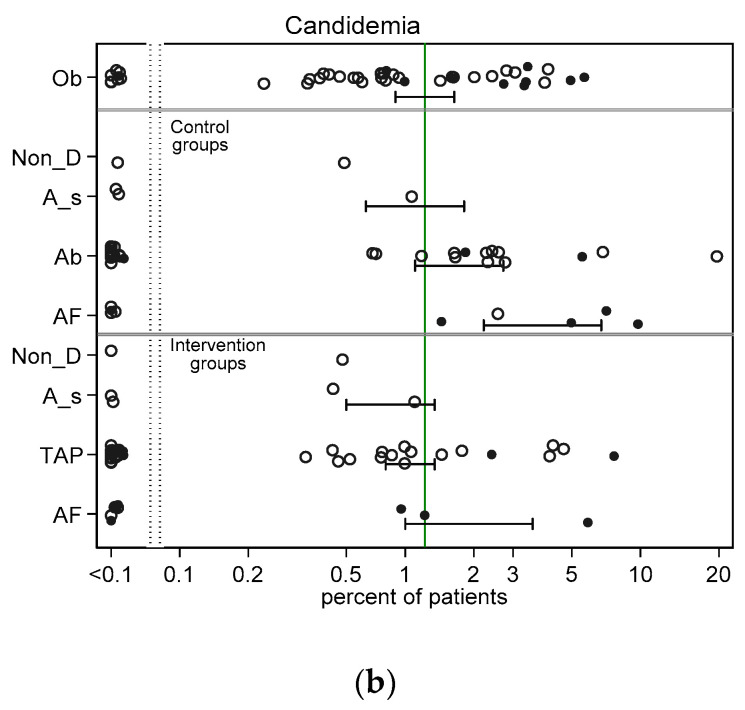
Scatter plots (logit scale) and 95% CI of RT Candida incidence (**a**) and Candidemia (**b**) in component (control and intervention) groups of various methods of infection prevention in the ICU, excluding studies with ICU-LOS < 5 days (ESM Figure S2a,b show including studies with ICU-LOS < 5 days). The benchmark incidence in each plot is the summary mean derived from the observation studies (central vertical line). The groups wide presence of candidemia risk factors (CRF) is identified by solid symbols versus not (open). Abbreviations; Ob = observational; non-D is non-decontamination; A_s is anti-septic; Ab is antibiotic control group; TAP is topical antibiotic prophylaxis and AF is anti-fungal.

**Figure 3:**
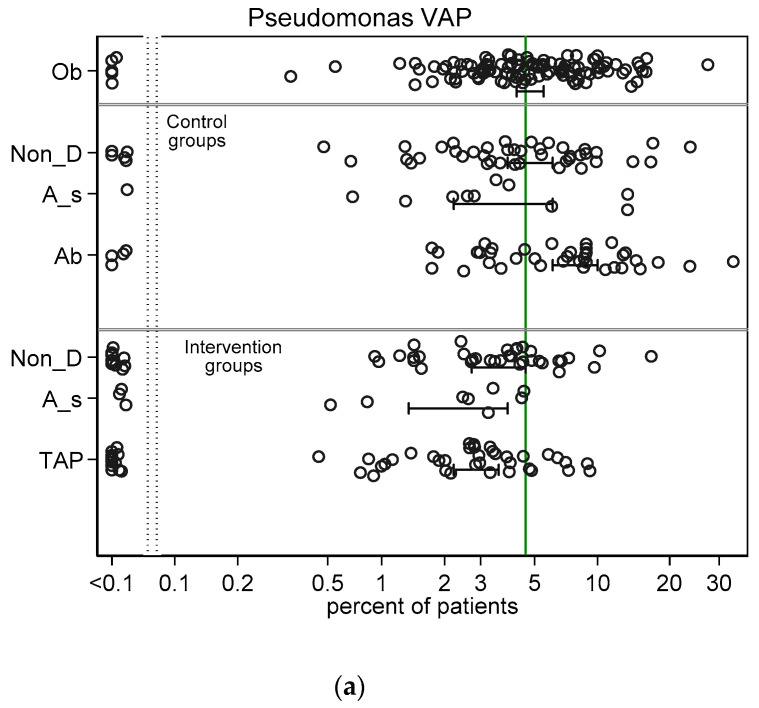

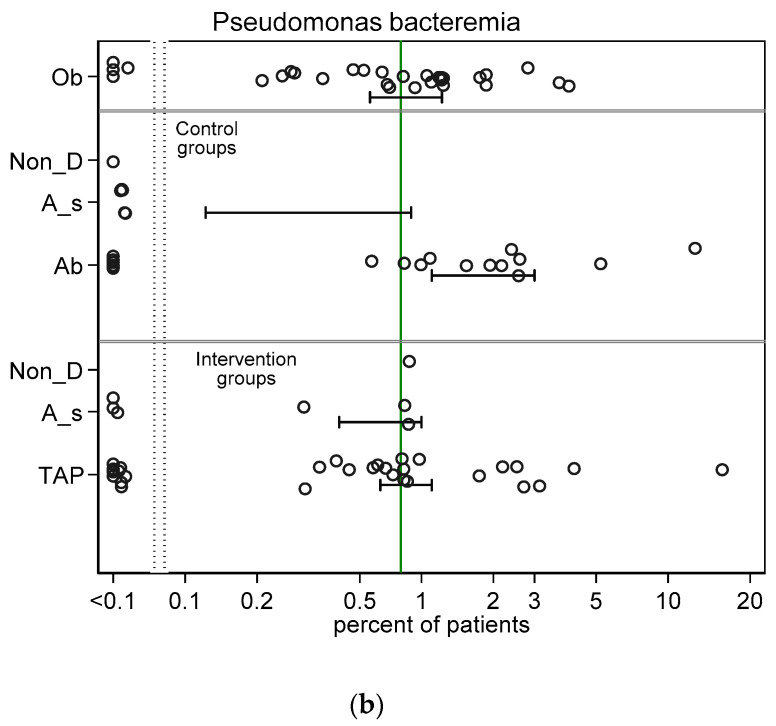
Scatter plots (logit scale) and 95% CI of *Pseudomonas* VAP incidence (**a**) and *Pseudomonas* bacteraemia (**b**) in component (control and intervention) groups of various methods of infection prevention in the ICU, excluding studies with ICU-LOS < 5 days (ESM Figure S3a,b show including studies with ICU-LOS < 5 days). The benchmark incidence in each plot is the summary mean derived from the observation studies (central vertical line). Abbreviations; Ob = observational; non-D is non-decontamination, A_s is anti-septic; Ab is antibiotic control group; TAP is topical antibiotic prophylaxis.

**Figure 4:**
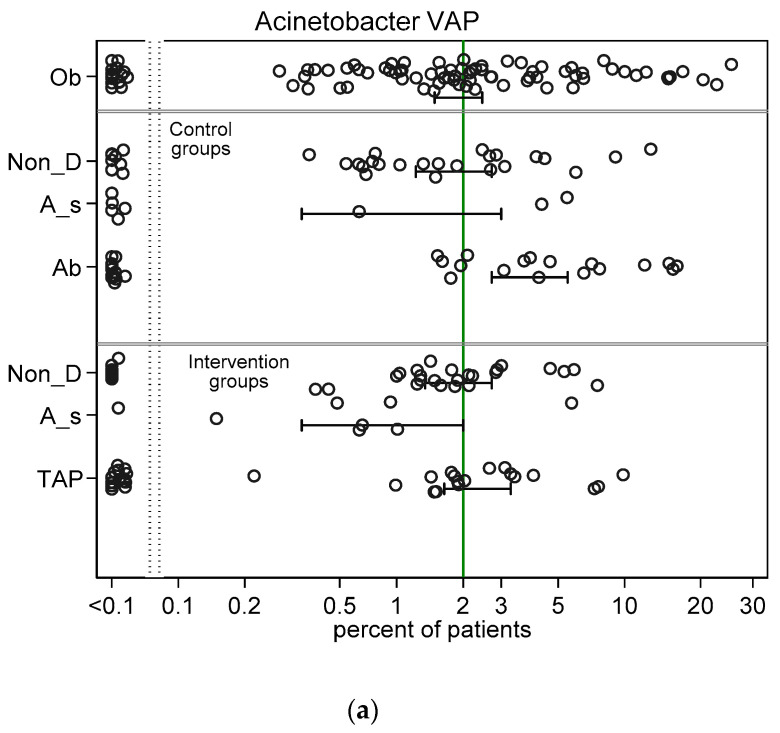

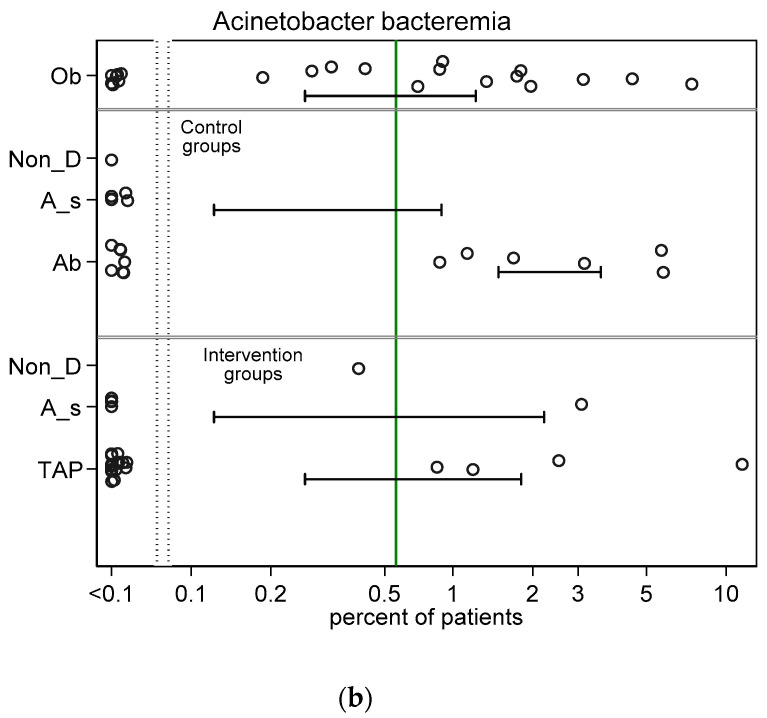
Scatter plots (logit scale) and 95% CI of *Acinetobacter* VAP incidence (**a**) and *Acinetobacter* bacteraemia (**b**) in component (control and intervention) groups of various methods of infection prevention in the ICU, excluding studies with ICU-LOS < 5 days (ESM Figure S4a,b show including studies with ICU-LOS < 5 days). The benchmark incidence in each plot is the summary mean derived from the observation studies (central vertical line). Abbreviations; Ob = observational; non-D is non-decontamination; A_s is anti-septic; Ab is antibiotic control group; TAP is topical antibiotic prophylaxis.

Across all intervention categories among groups with non-zero counts, the incidences for RT *Candida* (Figure 2a) and Candidemia (Figure 2b) and *Pseudomonas* VAP (Figure 3a) and bacteraemia (Figure 3b) and *Acinetobacter* VAP (Figure 4a) and bacteraemia (Figure 4b) in each case varied by >100 fold, ranging approximately ten-fold above and tenfold below the respective literature derived benchmark. In general, the mean incidence among each category of intervention group was up to 60% lower than the mean in the corresponding category of control group (Figure 2, Figure 3 and Figure 4).

The mean control and intervention group incidences of infection for VAP and bacteraemia for each of *Pseudomonas* (Figure 3) and *Acinetobacter* (Figure 4) were generally similar to the benchmark derived from observational groups with the following exceptions; the *Pseudomonas* VAP incidences among the antiseptic and TAP intervention groups were each approximately two percentage points below the *Pseudomonas* VAP benchmark and the mean incidences of infection for each of *Pseudomonas* (Figure 3) and *Acinetobacter* (Figure 4) for both VAP and bacteraemia among the control groups of TAP studies were each up to three percentage points higher than each benchmark.

### 3.3. GSEM Modelling

**Figure 5:**
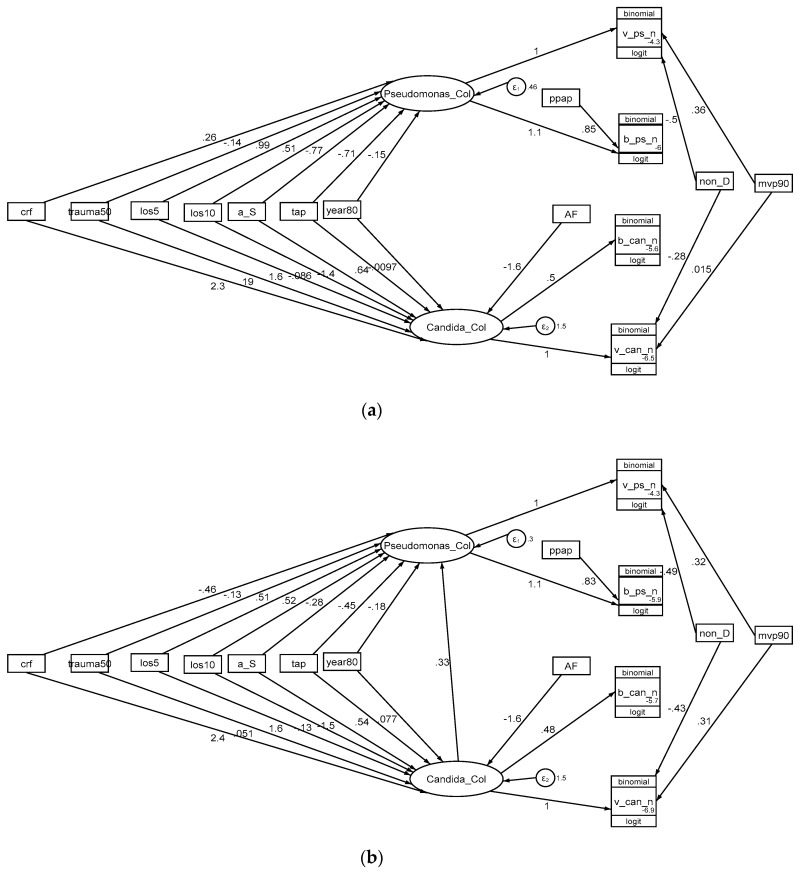
A GSEM founded on CAPI concepts without (**a**, model 1) and with (**b**, model 2) an interaction term between *Pseudomonas* colonization and *Candida* colonization. The model including in interaction term (model 2) is optimal. *Candida*_col, and *Pseudomonas*_col (ovals) are latent variables representing *Candida*, and *Pseudomonas* colonization, respectively. The variables in rectangles are binary predictor variables representing the group-level exposure to the following; a trauma ICU setting (trauma50), mean or median length of ICU stay < 5 days (los5), mean or median length of ICU stay ≥ 10 days (los10), year of study publication (year80), exposure to a topical anti-septic based prevention method (a_S), exposure to an anti-fungal based prevention method (AF), exposure to a TAP based prevention method (tap), exposure to a non-decontamination based prevention method (non-D), use of mechanical ventilation more for than 90% of the group (mvp90) or exposure to PPAP (ppap). The circles contain error terms (ε) associated with the latent variables. The three-part boxes represent the count data for *Candida*, and *Pseudomonas* VAP (v_can_n, v_ps_n) and bacteraemia (b_can_n, b_ps_n) which are each logit transformed with the total number of patients in each group as the denominator using the logit link function in the generalized model of the GSEM. Corresponding models (Models 3 to 8) for *Acinetobacter* colonization and models excluding studies with LOS < 5 days are shown in the ESM (Figures S5–S8).

Eight candidate GSEM models were evaluated for fit and parsimony (see Table 2; ESM Figures S5–S8). In each case, the optimal model included an interaction term between the latent variables representing *Candida* colonization with either *Pseudomonas* colonization (Figure 5, ESM Figures S5 and S6) or *Acinetobacter* colonization (ESM Figures S7 and S8). The size of this interaction term was similar in magnitude and significant in each model. The inclusion (four models; Table 2) or not (four models; ESM Tables S6 and S7) of 23 groups that had mean ICU-LOS less than 5 days made no material difference to the findings.

In the optimal model for *Pseudomonas* infection (Table 2, Figure 5), the following exposures; TAP, mean ICU-LOS > 10 days and the interaction term with *Candida* colonization, displayed the strongest associations with *Pseudomonas* colonization. Exposure to PPAP displayed a strong positive association with *Pseudomonas* bacteraemia counts and exposure to non-decontamination interventions a strong negative association with *Pseudomonas* VAP counts.

In the optimal model for *Acinetobacter* infection (Table 2, ESM Figure S7), the following exposures; mean ICU-LOS > 10 days and the interaction term with *Candida* colonization displayed the strongest associations with *Acinetobacter* colonization but the term for exposure to TAP was non-significant.

In all models, exposure to candidemia risk factors, anti-septic, and antifungal interventions displayed strong and consistent associations with *Candida* colonization. Other exposures were not consistently strong or significant in association with any of the three latent variables or with the infection data.

## 4. Discussion

There are multiple obstacles to defining the clinical relevance of the postulated CAPI model. *Candida* colonization has several predictors [35] some of which, such as prolonged antibiotic exposure, have broad effects on the microbiome. *Candida* [5] and bacterial colonization are problematic to define. How the interaction between *Candida* and bacteria might enhance the infection potential of either is likely more complex than simply co-location within the microbiome [4]. VAP is an imprecise endpoint. Bacteraemia with *Pseudomonas* and *Acinetobacter* are each uncommon, as is candidemia. Finally, data derived from concurrent control and intervention groups from single center clinical studies will exhibit dependency, especially so for infectious disease data.

Presumably as a result of these obstacles, attempts to validate the CAPI model have reported conflicting results. On the one hand, there is some evidence that the risk of VAP in association with *Pseudomonas* aeruginosa is more common in patients colonized by *C. albicans* [36] and that antifungal treatments can reduce this likelihood [37]. One study found that *Candida* colonization of the respiratory tract is associated with *Acinetobacter* VAP but not *Pseudomonas* VAP [38].

On the other hand, several attempts to address the CAPI model though either retrospective studies of the association with anti-fungal use or through studies of either pre-emptive or intensified prophylactic anti-fungal treatment [39,40,41] have failed to resolve the question. Several have questioned the specificity of the association and whether any association is simply a reflection of confounding by illness severity [42] or may depend on altered immune responses [43]. Even multi-centre studies may be underpowered to show differences for *Pseudomonas* bacteraemia, and more so *Acinetobacter* bacteraemia, being rare end points with benchmark incidences of 1.2% and 0.6%, respectively. An adequately powered study of these end points as a cluster randomized study would require >100,000 patients [44]. Moreover, they may be confounded by the geographic variation in the incidences of *Pseudomonas* VAP [45], and more so *Acinetobacter* VAP [46].

The approach here is to circumvent these obstacles by using, as a natural experiment, data from > 400 patient groups from > 250 studies of infection prevention interventions among ICU patients. The various groups of these studies have been exposed to infection prevention interventions which, in conjunction with other exposures, modify the patient microbiome. Of note, any one group here could experience multiple concurrent exposures such as concomitant CRF, TAP, PPAP, anti-fungal and ICU-LOS > 10 days. This is reflected in the wide range in incidences of infections across the >400 groups. Moreover, membership of a concurrent control group within a study of TAP is associated with significant one percentage point higher incidences of both candidemia [29] and also *Candida* as a respiratory tract isolate [28] in comparison to comparable patient groups in the literature.

The findings here recapitulate the findings from earlier analyses of these studies which demonstrated support for the concept that the control of gut overgrowth with *Acinetobacter* and *Pseudomonas* is key in the mediation of the effects of TAP based decontamination [27]. The analysis here includes in addition *Candida* infection data from these studies and also studies of single anti-fungal prophylaxis against invasive *Candida* infections.

SEM is emerging as a method in critical care research to model the relationships among multiple simultaneously observed variables in order to provide a quantitative test of any theoretical model proposed within the literature [33]. An ability to test the validity and inferred relationship of conceptual variables that cannot be directly quantified is achieved by using latent variables within the model. GSEM allows generalized linear response functions in addition to the linear response functions allowed by SEM.

The GSEM analysis takes a structural rather than statistical approach to the CAPI question. The structural approach means that a limited number of conceptually key group-level factors were entered as simple binary variables into intentionally simplistic GSEM models. There was no ability nor purpose to adjust for the underlying patient level risk. The true relationships between exposures and outcomes will likely be centre specific, complex, graded and with multiple exposure interactions. Specifically, the association coefficients for the three broad categories of anti-fungal, anti-septic and topical antibiotic based infection prevention interventions derived here may not reflect the magnitude of their true effect size within an optimally designed study. A statistical approach would use more conventional analytic methods such as meta-analysis which, being based on an assumption of exchangeability between control and intervention groups that randomized assignment of exposures provides, allows more precise effect size estimates for specific individual interventions.

### Limitations

There are five key limitations to this analysis, the first being that this analysis is a group-level modelling of three latent variables, *Candida* colonization, *Pseudomonas* colonization and *Acinetobacter* colonization. These latent variables are defined in each GSEM model but the derived coefficients are indicative and intended for internal reference only. They have no counterpart at the level of any one patient or study. Specifically, they are not estimates of colonization that might be measured by conventional methods at any one body site.

The second limitation is that there was considerable heterogeneity in the interventions, populations, and study designs among the studies here as the inclusion criteria for the various studies have been intentionally broadly specified. This breadth is both a strength, in that the breadth of the group wide exposures is the basis for the natural experiment here, and a limitation, in that the associations for a group wide exposure may not equate to associations at the level of an individual patient exposure.

Thirdly, several assumptions have been made for studies that failed to report key exposure and outcome variables in the analysis. For example, missing data for ICU-LOS and percent receiving MV has been broadly imputed. The extracted data are drawn mostly from studies located in systematic reviews and is provided in sufficient detail in the ESM to enable replication of the analysis.

Fourth, there are a large number of studies not included here because the required infection count data was not reported. However, the differences between control and intervention group mean *Acinetobacter* and *Pseudomonas* infection incidences noted here are similar to the summary effect sizes for each of the three broad categories of TAP, anti-septic and non-decontamination methods, against both overall VAP and against overall bacteraemia which in turn are similar to prior published effect estimates sizes seen in systematic reviews of these interventions from which most of the studies examined here were derived [6,7,8,9,10,11,12,13,14,15,16,17,18,19,20,21,22,23,24,25,26].

Fifth, each category of TAP, anti-septic and anti-fungal intervention include a broad range of specific interventions within the various studies. This is a deliberate simplification in the GSEM modelling as some, for example, the anti-fungal regimens, targeted different body sites. Additionally, the duration of application of the regimens varied among the studies. 

A strength of this analysis is that it attempts to unpack the separate associations between the *Acinetobacter* and *Pseudomonas* infection incidences and the variable exposure to the various SDD components (TAP, PPAP, anti-fungal). As previously noted, PPAP displays a strong positive association with *Pseudomonas* bacteraemia [27]. This is possibly not paradoxical as antibiotics used for PPAP typically lack activity against *Pseudomonas* and *Acinetobacter*. In this regard, the cumulative days of exposure to antibiotics without activity against *Pseudomonas* has been reported as being a risk factor for acquiring *Pseudomonas* and *Acinetobacter* in the ICU [47,48,49,50].

Another strength is that the results of the model is robust to the inclusion of groups with ICU-LOS < 5 days in a sensitivity test. These groups are of interest because of the increasing focus on ICU patient groups with a shorter overall length of stay. Likewise, whether patients receiving MV are the optimal patient group to target for decontamination is unclear and the analysis here is not limited to the MV patient group.

## 5. Conclusions

GSEM modelling of *Pseudomonas*, *Acinetobacter* and *Candida* colonization, each as latent variables, versus group-level exposures, provide support to the CAPI concept. The magnitude of the interaction between *Candida* colonization with indices of infections arising from *Acinetobacter* and *Pseudomonas* colonization could be as large as the association of these indices with TAP exposure. The CAPI interactions could explain the observed infection incidences being higher among the groups of TAP studies and the TAP interventions being paradoxically without effect against *Pseudomonas* VAP [51]. These observations raise further concerns about the safety of TAP as an infection prevention method used within the ICU context [52,53,54,55].

## Figures and Tables

**Table 1:** Characteristics of studies ^a^.

	Observational Studies	Infection Prevention Studies			
	(No Intervention)	Non-Decontamination	Anti-Septic	TAP ± PPAP	Anti-Fungal
Study characteristics					
Sources	ESM Table S1	ESM Table S2	ESM Table S3	ESM Table S4	ESM Table S5
Number of studies	147	47	16	67	9
Origin from systematic review ^b^	46	38	7	38	9
Study publication year (range)	1987–2018	1987–2017	2000–2016	1984–2018	1994–2014
North American ICU’s ^c^	36	11	7	6	0
Trauma ICUs ^d^	24	10	3	15	0
Group characteristics					
number of groups	164	94	35	102	20
LOS < 5 days	10	0	8	6	0
LOS > 10 days	101	54	18	97	15
MV for >48 h for <90% ^e^	40	0	16	33	8
PPAP use in control group ^f^	0	0	0	10	0
CRF ^g^	11	0	0	17	12
Numbers of patients per control group; (median; IQR) ^h^	279 118–604	75 60–143	130 36–347	61 318–123	65 51–75

^a^. Note, several studies had more than one control and or intervention group. Hence the number of groups does not equal the number of studies. Abbreviations; TAP = Topical antibiotic prophylaxis; PPAP = Protocolized parenteral antibiotic prophylaxis; ICU = Intensive Care Unit; ESM = electronic supplemental material (web-only supplement); LOS = length of stay; MV = mechanical ventilation. ^b^. Studies that were sourced from 16 systematic reviews (references in web-only supplement) ^c.^ Study originating from an ICU in Canada of the United States of America. ^d^. Trauma ICU arbitrarily defined as an ICU with more than 50% of admissions for trauma. ^e.^ Studies for which less than 90% of patients were reported to receive >48 h of MV. ^f^. Use of PPAP for control group patients. ^g^. CRF is risk factors for Candidemia or invasive *Candida* infection such as patient groups selected on the basis of *Candida* colonization. ^h.^ Data is median and inter-quartile range (IQR).

**Table 2:** Development of GSEM model ^a, b, c^.

Pseudomonas Models				Acinetobacter Models			
	Model 1	Model 2			Model 5	Model 6	
	Figure 5a and Figure S5a	Figure 5b and ESM Figure S5b			ESM Figure S7a	ESM Figure S7b	
			95%CI				95%CI
Factor ^d–i^				Factor ^d–i^			
b_Ps_n				b_Ac_n			
Pseudomonas colonization ^j^	1.09 ***	1.10 ***	0.71 to 1.5	Acinetobacter colonization ^k^	1.18 ***	1.17 ***	0.92 to 1.4
ppap	0.85 **	0.83 **	0.25 to 1.4	ppap	0.24	0.17	−0.87 to 1.4
_cons	−5.96 ***	−5.93 ***	−6.8 to −5.1	_cons	−8.14 ***	−8.08 ***	−9.2 to −6.9
v_Ps_n				v_Ac_n			
Pseudomonas colonization ^j^	1	1	(constrained)	Acinetobacter colonization ^k^	1	1	(constrained)
mvp90	0.36	0.32	−0.04 to 0.7	mvp90	0.58	0.54	−0.12 to 1.3
non_D	−0.50 ***	−0.49 ***	−0.76 to −0.2	non_D	−0.41	−0.37	−0.76 to 0.05
_cons	−4.33 ***	−4.31 ***	−5.1 to −3.5	_cons	−6.56 ***	−6.56 ***	−7.8 to −5.3
Pseudomonas colonization ^j^				Acinetobacter colonization ^k^			
TAP	−0.71 ***	−0.45 ***	−0.71 to −0.2	TAP	−0.69 **	−0.43	−1.1 to 0.0
year	−0.15 *	−0.18 **	−0.31 to −0.1	year	0.17	0.17	−0.12 to 0.4
los5	0.99 *	0.51	−0.20 to 1.2	los5	0.76	0.34	−0.4 to 1.3
los10	0.51 ***	0.52 ***	0.28 to 0.7	los10	0.94 ***	0.91 ***	0.46 to 1.3
trauma	−0.14	−0.13	−0.47 to 0.2	trauma	0.44	0.4	−0.03 to 0.95
Anti-septic	−0.77 ***	−0.28	−0.77 to 0.2	Anti-septic	−1.09 **	−0.59	−1.5 to 0.1
crf	0.26	−0.46	−1.2 to 0.2	crf	−0.27	−0.95	−3.1 to 1.2
*Candida* colonization ^l^	-	0.33 ***	0.22 to 0.45	*Candida* colonization ^l^	-	0.32 *	0.01 to 0.5
b_can_n				b_can_n			
*Candida* colonization ^l^	0.50 ***	0.48 ***	0.3 to 0.64	*Candida* colonization ^l^	0.49 ***	0.49 ***	0.3 to 0.67
_cons	−5.62 ***	−5.66 ***	−6.2 to −5.1	_cons	−5.62 ***	−5.56 ***	−6.1 to −4.9
v_can_n				v_can_n			
*Candida* colonization ^l^	1	1	(constrained)	*Candida* colonization ^l^	1	1	(constrained)
mvp90	0.02	0.31	−0.53 to 1.1	mvp90	0.02	0.12	−0.80 to 0.89
non_D	−0.28	−0.43	−1.1 to 0.21	non_D	−0.28	−0.27	−0.97 to 0.38
_cons	−6.53 ***	−6.86 ***	−8.4 to −5.3	_cons	−6.53 ***	−6.51 ***	−8.1 to −4.9
*Candida* colonization ^l^				*Candida* colonization ^l^			
TAP	0.64	0.54	−0.31 to 1.4	TAP	0.64	0.64	−0.22 to 1.5
year	−0.01	0.08	−0.26 to 0.4	year	−0.01	−0.02	−0.36 to 0.28
los5	1.62 **	1.59 **	0.62 to 2.7	los5	1.62 **	1.55 **	0.51 to 2.7
los10	−0.09	−0.13	−0.59 to 0.4	los10	−0.09	−0.08	−0.55 to 0.5
trauma	0.19	0.05	−0.9 to 0.9	trauma	0.19	0.16	−0.83 to 0.99
Anti-septic	−1.44 **	−1.47 **	−2.4 to −0.6	Anti-septic	−1.44 **	−1.39 **	−2.4 to −0.5
Anti-fungal	−1.58 ***	−1.6 ***	−2.5 to −0.72	Anti-fungal	−1.58 ***	−1.6 ***	−2.5 to −0.69
crf	2.10 ***	2.25 ***	1.2 to 3.3	crf	2.10 ***	2.16 ***	1.05 to 3.1
Error terms				Error terms			
var (e. *Candida* colonization)	1.49 ***	1.49 ***	1.0 to 2.3	var (e. *Candida* colonization)	1.49 ***	1.47 ***	1.0 to 2.3
var (e. Pseudomonas colonization)	0.46 ***	0.30 ***	0.20 to 0.44	var (e. Acinetobacter colonization)	1.42 ***	1.27 ***	0.95 to 1.7
Model fit ^m^				Model fit ^m^			
AIC	3631.39	3600.45	-	AIC	2723.04	2717.31	-
Groups (n)	439	439	-	Groups (n)	395	395	-
Clusters (n)	276	276		Clusters (n)	247	247	-
Factors	30	31	-	Factors	30	31	-

^a^. Legend: * *p* < 0.05; ** *p* < 0.01; *** *p* < 0.001. ^b^. Shown in this table are models derived with all studies (models 1, 2, 5 and 6). ^c^. Models derived after excluding studies with LOS < 5 days (models 3, 4, 7 and 8) are shown in the ESM (ESM Tables S6 and S7). The figures corresponding to models 1 (Figure S5a), model 2 (Figure 1 and ESM Figure S5b), model 5 (ESM Figure S7a) and Model 6 (ESM Figure S7b). ^d^. Abbreviations; v_ps_n is the count of *Pseudomonas* VAP; ^e^. v_ac_n is the count of *Acinetobacter* VAP; ^f^. v_can_n is the count of *RT Candida*; ^g^. b_ps_n is the count of *Pseudomonas* bacteraemia and ^h^. b_ac_n is the count of *Acinetobacter* bacteraemia; ^i^. b_can_n is the count of Candidemia; VAP is ventilator-associated pneumonia; non-D is non-decontamination, A_s is anti-septic; Ab is antibiotic control group; TAP is topical antibiotic prophylaxis. PPAP is the group wide use of protocolized parenteral antibiotic prophylaxis; tap is topical antibiotic prophylaxis; non-D is a non-decontamination intervention; year = year of study publication in units of ten (decade). MVP90 is use of mechanical ventilation by more than 90% of the group. Crf is group wide exposure to a candidemia risk factor. LOS5 is a mean or median length of ICU stay for the group of less than 5 days. LOS10 is a mean or median length of ICU stay for the group of more than 10 days. Trauma ICU arbitrarily defined as an ICU for which >50% of admissions were for trauma. ^j^. Pseudomonas colonization (Pseudomonas col) is a latent variable. ^k^. Acinetobacter colonization (Acinteobacter col) is a latent variable. ^l^. *Candida* colonization (*Candida* col) is a latent variable. ^m^. Model fit; AIC is Akaike’s information criteria. This indicates model fit taking into account the statistical goodness of fit and the number of parameters in the model. Lower values of AIC indicate a better model fit. Groups is the number of patient groups; clusters is the number of studies; N is the number of parameters in the model.

## Supplementary Materials

The following are available online at https://www.mdpi.com/2309-608X/6/4/252/s1, Table S1: Observational studies (Benchmark groups); Table S2: Groups of non-decontamination studies; Table S3: Groups of anti-septic studies; Table S4: Groups of TAP studies; Table S5: Groups from anti-fungal studies; Table S6: Development of *Pseudomonas* GSEM models (models 1–4); Table S7: Development of *Acinetobacter* GSEM models (models 5–8); Supplementary References; Figure S1. Search, screening, triage and decant of studies and groups; Figure S2a,b. Candidemia and RT *Candida* count data; Figure S3a,b. Pseudomonas; VAP and bacteraemia count data; Figure S4a,b. *Acinetobacter*; VAP and bacteraemia count data; Figure S5a,b. *Pseudomonas* GSEM models 1 and 2 (all studies); Figure S6a,b. *Pseudomonas* GSEM models 3 and 4; (Excluding studies with ICU-LOS < 5 days); Figure S7a,b. *Acinetobacter* GSEM models 5 and 6 (all studies); Figure S8a,b. *Acinetobacter* GSEM models 5 and 6.
