# Supplementary material for: Candida–Acinetobacter–Pseudomonas Interaction Modelled within 286 ICU Infection Prevention Studies
Source: J Fungi (Basel). 2020 Oct 27;6(4):252. doi: 10.3390/jof6040252 (PMC7712580; doi:10.3390/jof6040252)

Candida-Acinetobacter-Pseudomonas-interaction modelled within 286 ICU infection prevention studies

James C Hurley, MB BS, D Med Sci, M Epi, PhD, FRACP;

Author affiliations;

- Associate Professor, Melbourne Medical School, University of Melbourne and Physician, Division of Internal Medicine, Ballarat Health Services, Ballarat, Victoria, Australiae; jamesh@bhs.org.au

Supplemental file contents:

Table S1: Observational studies (Benchmark groups) 2 - 6

Table S2: Groups of non-decontamination studies 7- 10

Table S3: Groups of anti-septic studies 11 – 12

Table S4: Groups of TAP studies 13 – 18

Table S5: Groups from anti-fungal studies 19

Table S6: Development of *Pseudomonas* GSEM models (models 1-4) 20 – 22

Table S7: Development of *Acinetobacter* GSEM models (models 5-8) 23 – 25

References 26 – 44

Fig S1. Search, screening, triage and decant of studies and groups 44 - 45

Fig S2 a & b. Candidemia and RT candida count data 46

Fig S3 a & b. *Pseudomonas*; VAP and bacteremia count data 47

Fig S4 a & b. *Acinetobacter*; VAP and bacteremia count data 48

Fig S5 *a & b. Pseudomonas* GSEM models 1 & 2 (all studies) 49

Fig S6 *a & b. Pseudomonas* GSEM models 3 & 4

(*Excluding studies with ICU-LOS<5 days)* 50

Fig S7 *a & b.* Acinetobacter GSEM models 5 & 6 (all studies) 51

Fig S8 *a & b.* Acinetobacter GSEM models 5 & 6

(*Excluding studies with ICU-LOS<5 days)* 52

Table S1: Observational studies (Benchmark groups) [a]

Author	Year	Ref	Notes	MV	LOS	Patients	VAP			Bacteremia		
				%	d	n	v_ps_n	v_ac_n	v_can_n	b_ps_n	b_ac_n	b_can_n
A'court	1993	1	T	100	>10	150	17	0		6		
Alvarez -Lerma	1996	2		93	5to10	6494	174	56				
Antonelli	1994	3		70	>10	124	5	6	1			
Apostolo-poulou	2003	4		100	>10	175	17	6				
Arumugam	2018	5	T	100	5to10	332	7	4				
Azoulay	2006	6		100	>10	589	30					4
Azoulay	2006	6	crf	100	>10	214	19					2
Bailly	2015	7		100	>10	1491						11
Baraibar	1997	8		100	5to10	707		12				
Beck-Sague	1996	9		100	5to10	145	5	0				
Bekaert	2011	10		100	5to10	4479	155	24				
Bercault	2001	11		100	>10	1144			2			
Bercault_ IHT	2005	12		100	5to10	118	9	2				
Bercault _noINT	2005	12	I	100	>10	118	3	3				
Berrouane	1998	13		83	>10	565	40					
Blot 45_64	2014	14		100	5to10	670	20	15				
Blot 65_74	2014	14		100	5to10	549	20	14				
Blot >74	2014	14		100	5to10	516	10	8				
Bochicchio	2004	15	T	100	>10	678	22	10				
Bonten'94	1994	16		100	>10	64	6	0				
Bonten'96	1996	17		100	>10	141	12					
Boots	2008	18		100	>10	412	15	14	0			
Bornstain	2004	19		100	>10	747	23	1				
Borzotta	1999	20		85	5to10	459						7
Braun	1986	21		100	5to10	66	0					
Bregeon	1997	22		100	NS	660	33	10	3			
Bronchard	2004	23	T	100	>10	109	0					
Cade	1993	24		98	>10	98	4	0	5	1	0	1
Cavalcanti	2006	25		100	5to10	190	9	1	6			
Cenderero	1999	26		100	5to10	123	4	2	0			
Chaari	2015	27		100	5to10	175	20	12				
Charles	2005	28		75	>10	36						0
Charles	2005	28	crf	97	>10	56						1
Chastre	1998	29		100	>10	243	36	13	7			
Chevret	1993	30		100	5to10	255	21	5	4			
Combes	2000	31	T	100	>10	104	0					

Table S1 (continued): Observational studies (Benchmark groups)

Author	Year	Ref	Notes	MV	LOS	Patients	VAP			Bacteremia		
				%	d	n	v_ps_n	v_ac_n	v_can_n	b_ps_n	b_ac_n	b_can_n
Cook_ non-trauma	2010	32		100	5-10	2080	7	1	4			
Cook_trauma	2010	32	T	100	>10	511	16	8	1			
Craven-medical	1988	33		100	5-10	277	9	0	1	1		0
Craven-surgical	1988	33		100	5-10	521	17	0	5	6		3
Daschner	1988	34		100	5-10	116	9	1	4			
De waele	2003	35	crf	79	>10	28						0
de_Latorre	1995	36		100	>10	80	7	0	2			
de_Santis	2000	37		NS	NS	713				3		2
de_Santis	2013	37		NS	NS	1318				1		3
El-Masri	2004	38		NS	>10	361				1	3	6
Ensminger	2006	39	C	100	5-10	92	2	1				
Ertugrul	2006	40		100	5-10	100	3	6		0	2	
Esteve	2007	41		80	>10	404				8	1	0
Esteve	2007	41		78	>10	395				4	6	2
Evans	2010	42		100	5-10	416	18					
Ewig	1999	43		100	5-10	48	4	0	1			
Fabian	1993	44	T	100	>10	278	10	15				
Fagon	1989	45		100	>10	567	16	8				
Ferreira	2015	46		94	5-10	2527						23
Gacouin	2009	47		100	>10	361	21	4				
Garcı´a -Garmendia	2001	48		46	5-10	2640				14	42	11
Garrouste -Orgeas	1997	49		100	>10	86	9	11	1			
Garrouste -Orgeas	2006	50		75	>10	3247				19	1	15
George	1998	51		100	5-10	223	6	1	2			
Georges	2000	52		100	>10	135	19	8				
Giamarellos-Bourboulis	2009	53	T	100	>10	72	5	17	2	1	2	3
Giard	2008	54		100	5-10	7236	168					
Gruson-95-96	2000	55		100	NS	1004	62	20				
Gruson-97-98	2000	55	I	100	NS	1029	47	7				
Gruson-99-01	2003	56		100	NS	823	41	15				
Guérin	1997	57		100	>10	260	14	1	0			
Gursel	2010	58		100	5-10	92	10	23	1			
Heyland	1999	59		100	5-10	1014	38	6	26			5
Holzapfel_93	1993	60		100	5-10	300	0	1		0		
Huang_1SC	2013	61		NS	<5	15816				14	7	49
Huang_1pre	2013	61		NS	<5	23480				5	9	38

Table S1 (continued): Observational studies (Benchmark groups)

Author	Year	Ref	Notes	MV	LOS	Patients	VAP			Bacteremia		
				%	d	n	v_ps_n	v_ac_n	v_can_n	b_ps_n	b_ac_n	b_can_n
Hugonnet	2007	62		100	5-10	936	31	8	40			
Hyllienmark	2007	63		100	<5	221	0	1				
Hyllienmark	2013	64	T	42	<5	135	4	2				
Ibáñez	2000	65		100	5-10	30	1	1				
Ibrahim'00	2000	66		100	5-10	1882	130	16	19			
Ibrahim'00	2000	67		69	>10	4913				22	8	41
Ibrahim'01	2001	68		56	5-10	880	49	4				
Jacobs	1990	69		100	>10	24	4	0		1		
Jaillette	2011	70		100	>10	439	59	22				
Jensen_HE	2015	71		66	5-10	604						24
Jensen_SOC	2015	71		67	5-10	596						13
Jimenez	1989	72		100	5-10	77	7	6				
Kautzky	2014	73		37	>10	35			1			0
Kautzky	2014	73	crf	57	>10	30			1			2
Ko	2013	74		100	>10	1453				21	11	
Kollef' 93	1993	75		100	5-10	277	4	1				
Kollef '95	1995	76		100	NS	314	16	4				
Kollef '95	1995	77		100	>10	300	23	6				
Kollef '97	1997	78		100	5-10	521	15	2				
Kollef _post	1997	79	C I	90	<5	327	3	1	0	1	0	3
Kollef _pre	1997	79	C	90	<5	353	7	2	1	2	1	5
Kollef _Europe	2014	80		100	>10	495	24					
Kollef _USA	2014	80		100	>10	502	17					
Koss– N	2001	81		100	>10	87	4	2	10			
Koss– P	2001	81	I	100	>10	66	9	2	3			
Kunac	2014	82		100	NS	716	23	13		2	1	
Laggner	1989	83		100	>10	32	0	0	0			0
Lambert	2011	84		NS	5-10	119699				389	143	
Laupland	2002	85		NS	5-10	1017				2	0	3
Laupland	2004	86		84	5-10	4473				11	4	19
León	2006	87	crf	95	>10	1699						58
León	2009	88	crf	91	>10	1107						37
León	2016	89	crf	84	>10	233						11
Lepelletier	2010	90	T	100	>10	161	10					
Li	2012	91		17	>10	29			1			0
Li	2012	91	crf	40	>10	82			10			3
Luna	2003	92		100	5-10	427	13	25	2			

Table S1 (continued): Observational studies (Benchmark groups)

Author	Year	Ref	Notes	MV	LOS	Patients	VAP			Bacteremia		
				%	d	n	v_ps_n	v_ac_n	v_can_n	b_ps_n	b_ac_n	b_can_n
Luyt	2005	93		100	NS	290	11	3				
Magnason	2008	94		100	5-10	280	5	0	0	0	0	5
Mahul	1992	95		100	>10	145	8	3	3			
Makris	2011	96	I	100	>10	152	7					
Markowicz	2000	97		100	5-10	744	58	13				
Memish	2000	98		100	>10	202	21	14	4			
Michel	2005	99		100	>10	299	12					
Mitsogianni	2011	100		NS	>10	124				1	5	1
Mitsogianni	2010	100		NS	>10	143				2	12	0
Moine	2002	101		80	>10	764	27	1	2			
Montecalvo	1992	102		100	5-10	38				1		1
Myny	2005	103		100	<5	385	28	13				
Nguile-Makao	2010	104		100	5-10	2873	130					
Nielsen	1992	105		100	5-10	242	3	0				
Nseir	2005	106		100	5-10	1241	32	20				
Nseir	2007	107	crf	100	>10	102						3
Orsi	2007	108		98	>10	1741				29	16	10
Orsi	2012	108		100	>10	1165				12	23	4
Osmon	2003	109		72	5-10	893				9		26
Outcomerea	2019	110		100	5-10	7735	454	48				
Papazian	1996	111		100	NS	586	26	5				
Petri	1997	112	crf	95	>10	409						3
Potgieter	1987	113		78	5-10	250	26	32	5			
Prowle	2011	114		69	5-10	6339						51
Ramirez	2016	115		100	>10	440			0			
Rello'91	1991	116		100	5-10	264	14	2	1			
Rello'92	1992	117		80	5-10	208	10					
Rello'92	1992	118		67	5-10	161	4					
Rello'94	1994	119		72	NS	1650				16	5	4
Rello'96	1996	120		100	>10	83	4					
Rello'02	2002	121		100	5-10	9080	119					
Rello'03	2003	122		100	>10	99	8	2	0			
Resende	2013	123		100	>10	126	11	11	0			
Reusser	1989	124		100	5-10	40	2	1		0	0	
Rincón-Ferrari	2004	125		100	5-10	310	6	27				
Rodrigues	2009	126		100	5-10	133	10	14	2			
Rodriguez	1991	127	T	100	>10	294	31	11				
Ruiz-Santana	1987	128		100	5-10	1005	56	0	1			

Table S1 (continued): Observational studies (Benchmark groups)

Author	Year	Ref	Notes	MV	LOS	Patients	VAP			Bacteremia		
				%	d	n	v_ps_n	v_ac_n	v_can_n	b_ps_n	b_ac_n	b_can_n
Salata	1987	129		100	>10	51	7	0	1			
Shahin	2013	130		100	5to10	267	4		3			
Sofianou	2000	131		100	>10	198	19	35				
Stéphan	2006	132	T	100	>10	175	14	8				
Tan	2016	133		100	>10	618	49	114				
Tejada-Artigas	2001	134		100	>10	103	5	10				0
Thompson	2008	135		NS	5to10	4270				13	1	24
Timsit	1996	136		100	>10	387	11	8				
Torres	1990	137		100	<5	322	5	9				
Trouillet	1998	138		100	>10	498	39	22				
Urli	2002	139		95	>10	178	27		1	3		4
Valles	2007	140		100	>10	60	15	0				
Vanhems	2011	141		100	5to10	3387	24					
Verhamme	2007	142		84	5to10	4000	54	3	6			
Violan	1998	143		100	>10	314	25	1				
Warren	2001	144		28	<5	3163				3	0	4
Woske	2001	145		100	>10	103	8	1				
Xie	2011	146		100	5to10	4155	169	137	88			
Zahar	2009	147		100	5to10	1233	62					

Table S1 footnotes

Notes; T = Data originating from a study for which the majority of ICU admission were for trauma; C = cardio-thoracic ICU; I = Infection control intervention to entire ICU; crf = group wide candidemia risk factor

MV = percentage of group receiving mechanical ventilation; NS – Not stated; LOS is mean or median length of ICU stay; The ICU-LOS is the ICU length of stay. This is based on surrogate measures including mean (or median) length of MV were taken if the length of ICU LOS was not available in order to generate broad categories of ICU stay of <5 days, 5 to 10 days and >10 days

v_ps_n is the count of *Pseudomonas* VAP; v_sr_n is the count of *Staphylococcus aureus* VAP; v_ac_n is the count of *Acinetobacter* VAP and v_can_n is the count of *Candida* isolates from patients with VAP.

b_ps_n is the count of *Pseudomonas* bacteremia; b_sr_n is the count of *Staphylococcus aureus* bacteremia; and b_ac_n is the count of *Acinetobacter* bacteremia; b_can_n is the count of Candidemia; b_cns_n is the count of coagulative negative *Staphylococcus* bacteremia and b_ent_n is the count of *Enterococcal* bacteremia.

Several (n = 43) of these studies were cited in the following source systematic reviews.

- Melsen WG, Rovers MM, Bonten MJM: Ventilator-associated pneumonia and mortality: A systematic review of observational studies. *Crit Care Med* 2009, 37:2709–2718.
- Safdar N, Dezfulian C, Collard HR, Saint S: Clinical and economic consequences of ventilator-associated pneumonia: a systematic review. *Crit Care Med* 2005, 33:2184–93.
- Agrafiotis M, Siempos II, Ntaidou TK, Falagas ME. Attributable mortality of ventilator-associated pneumonia: a meta-analysis. *Intern J Tub Lung Dis.* 2011;15(9):1154-1163.

Table S2: Groups of non decontamination studies [a]

Author	Year	Ref	Notes	MV	LOS	Patients	VAP			Bacteremia		
				%	d	n	v_ps_n	v_ac_n	v_can_n	b_ps_n	b_ac_n	b_can_n
Acosta -escribano	2010	148	T	100	>10	54	4	0	1			
Acosta -escribano	2010	148	T	100	>10	50	3	0	0			
Bonten '95	1995	149		100	>10	67	11	0				0
Bonten '95	1995	149		100	>10	74	7	1				0
Cook	1998	150		100	5to10	604	21		11			
Cook	1998	150		100	5to10	596	20		19			
Daumal	1999	151		100	>10	174	11		2			
Daumal	1999	151		100	5to10	187	12		6			
Djedaini	1995	152		100	5to10	68	3	0	1			
Djedaini	1995	152		100	5to10	61	0	3	0			
Drakulovic	1999	153		100	5to10	39	1	0	0			
Drakulovic	1999	153		100	5to10	47	3	1	0			
Dreyfuss	1991	154		100	5to10	28	1	2				
Dreyfuss	1991	154		100	>10	35	3	3				
Dreyfuss	1995	155		100	5to10	70	1	2				
Dreyfuss	1995	155		100	>10	61	0	3				
Driks	1987	156		100	>10	61	1		0			
Driks	1987	156		100	>10	69	5		0			
Forestier	2008	157		100	>10	106	8					
Forestier	2008	157		100	>10	102	3					
Heyland	1999	158		100	>10	49	0		0			
Heyland	1999	158		100	>10	46	0		0			
Holzapfel_C	1999	159		100	>10	200	6	3	3	0	0	1
Holzapfel_I	1999	159		100	>10	199	10	4	1	2	1	1
Kappstein	1991	160		100	5to10	49			3			
Kappstein	1991	160		100	5to10	55			7			
Kirschenbaum	2002	161		100	>10	20	5					
Kirschenbaum	2002	161		100	>10	17	1					
Kirton	1997	162		100	>10	140	6					
Kirton	1997	162		100	>10	140	6					
Knight	2009	163		100	5to10	130	0	3	0			
Knight	2009	163		100	5to10	129	1	1	0			
Kollef	2008	164		100	5to10	743	11	5	7			
Kollef _silverETT	2008	164		100	5to10	766	8	1	5			
Kortbeek	1999	165	T	100	5to10	37	0	1				
Kortbeek	1999	165	T	100	5to10	43	0	1				

Table S2 (continued): Groups of non decontamination studies [a]

Author	Year	Ref	Notes	MV	LOS	Patients	VAP			Bacteremia		
				%	d	n	v_ps_n	v_ac_n	v_can_n	b_ps_n	b_ac_n	b_can_n
Lacherade	2005	166		100	>10	185	9	2	0			
Lacherade	2005	166		100	>10	184	14	0	2			
Lacherade	2010	167		100	>10	164	16	2				
Lacherade	2010	167		100	>10	169	9	2				
Laueny	2014	168		100	>10	98	0	1	0			
Laueny	2014	168		100	>10	91	0	0	0			
Lorente '03	2003	169		100	>10	114	9	0	14			
Lorente '03	2003	169		100	>10	116	10	1	10			
Lorente '04	2004	170		100	>10	161	9	3	2			
Lorente '04	2004	170		100	>10	143	8	1	3			
Lorente '05	2006	171		100	>10	210	12	1	2			
Lorente '05	2006	171		100	>10	233	12	2	1			
Lorente '06a	2005	172		100	5to10	236	9	1	1			
Lorente '06a	2005	172		100	5to10	221	7	1	1			
Lorente '06b	2006	173		100	>10	53	2		0			
Lorente '06b	2006	173		100	>10	51	5		0			
Lorente '07	2007	174		100	>10	140	4	3	0			
Lorente '07	2007	174		100	>10	140	4	1	0			
Lorente'14	2014	175		100	>10	150	6	1				
Lorente'14	2014	175		100	>10	134	3	0				
Manzano	2008	176		100	>10	63	0	4				
Manzano	2008	176		100	5to10	64	0	2				
Martin	1993	177		100	5to10	65	0	1	1			
Martin	1993	177		100	5to10	66	2	0	0			
Morrow	2010	178		100	>10	73	0	3	0			
Morrow	2010	178		100	>10	73	6	2	1			
Nseir	2011	179		100	5to10	61	2	2				
Nseir	2011	179		100	>10	61	0	1				
Pickworth	1993	180	T	100	5to10	44	1		0			
Pneumatikos	2006	181		100	>10	39	0	1	0			
Pneumatikos	2006	181		100	>10	40	1	1	0			
Prod'hom_A	1994	182		100	5to10	81	4	0				
Prod'hom_R	1994	182		100	5to10	80	1	1				
Prod'hom_S	1994	182		100	5to10	83	1	0				
Reigneir	2013	183		100	5to10	227	12					
Reigneir	2013	183		100	5to10	222	9					

Table S2 (continued): Groups of non decontamination studies [a]

Author	Year	Ref	Notes	MV	LOS	Patients	VAP			Bacteremia		
				%	d	n	v_ps_n	v_ac_n	v_can_n	b_ps_n	b_ac_n	b_can_n
Rumbak	2004	184		100	5to10	60	1					
Rumbak	2004	184		100	>10	60	5					
Ryan_C	1993	185		100	5to10	56	2	1				
Ryan_S	1993	185		100	5to10	58	1	0				
Smulders	2002	186		100	>10	75	3		1			
Smulders	2002	186		100	>10	75	1		0			
Staudinger	2010	187		100	>10	75	5		2			
Staudinger	2010	187		100	5to10	75	3		0			
**Thomachot**	1998	188		100	>10	66	3	1	0			
**Thomachot**	1998	188		100	>10	70	2	1	0			
**Thomachot**	1999	189		100	>10	77	1	0	0			
**Thomachot**	1999	189		100	>10	63	2	0	0			
**Thomachot**	2002	190		100	5to10	71	0	1				
**Thomachot**	2002	190		100	5to10	84	2	0				
**Valencia**	2007	191		100	>10	69	1	0				
**Valencia**	2007	191		100	>10	73	3	1				
**Valles**	1995	192		100	5to10	77	12					
**Valles**	1995	192		100	5to10	76	12					
**Walaszek**	2017	193		100	5to10	804	5	39	5			
**Walaszek**	2017	193		100	5to10	1003	10	22	1			
**Zeng**	2016	194		100	>10	118	13	10	2			
**Zeng**	2016	194		100	>10	117	19	14	4			

Table S2 footNotes

Notes; T = Data originating from a study for which the majority of ICU admission were for trauma; C = cardio-thoracic ICU; I = Infection control intervention to entire ICU; crf = group wide candidemia risk factor

MV = percentage of group receiving mechanical ventilation; NS – Not stated; LOS is mean or median length of ICU stay, The ICU-LOS is the ICU length of stay. This is based on surrogate measures including mean (or median) length of MV were taken if the length of ICU LOS was not available in order to generate broad categories of ICU stay of <5 days, 5 to 10 days and >10 days

v_ps_n is the count of *Pseudomonas* VAP; v_sr_n is the count of *Staphylococcus aureus* VAP; v_ac_n is the count of *Acinetobacter* VAP and v_can_n is the count of *Candida* isolates from patients with VAP.

b_ps_n is the count of *Pseudomonas* bacteremia; b_sr_n is the count of *Staphylococcus aureus* bacteremia; and b_ac_n is the count of *Acinetobacter* bacteremia; b_can_n is the count of Candidemia; b_cns_n is the count of coagulative negative *Staphylococcus* bacteremia and b_ent_n is the count of *Enterococcal* bacteremia.

Several (n = 47) of these studies were cited in the following source systematic reviews.

- Messori A, Trippoli S, Vaiani M, Gorini M, Corrado A: Bleeding and pneumonia in intensive care patients given ranitidine and sucralfate for prevention of stress ulcer: meta-analysis of randomised controlled trials. *BMJ* 2000, 321:1103–1106.
- Huang J, Cao Y, Liao C, Wu L, Gao F: Effect of histamine-2-receptor antagonists versus sucralfate on stress ulcer prophylaxis in mechanically ventilated patients: a meta-analysis of 10 randomized controlled trials. *Crit Care* 2010, 14:R194.
- Alhazzani W, Almasoud A, Jaeschke R, Lo BW, Sindi A, Altayyar S, Fox-Robichaud A: Small bowel feeding and risk of pneumonia in adult critically ill patients: a systematic review and meta-analysis of randomized trials. *Crit Care* 2013, 17:R127.
- Melsen WG, Rovers MM, Bonten MJM: Ventilator-associated pneumonia and mortality: A systematic review of observational studies. *Crit Care Med* 2009, 37:2709–2718.
- Safdar N, Dezfulian C, Collard HR, Saint S: Clinical and economic consequences of ventilator-associated pneumonia: a systematic review. *Crit Care Med* 2005, 33:2184–93.
- Han J, Liu Y. Effect of ventilator circuit changes on ventilator-associated pneumonia: a systematic review and meta-analysis. *Respiratory care*, 2010; 55: 467-474.
- Subirana M, Solà I, Benito S: Closed tracheal suction systems versus open tracheal suction systems for mechanically ventilated adult patients. *Cochrane Database Syst Rev* 2007, 4: CD004581;
- Siempos II, Vardakas KZ, Kopterides P, Falagas ME. Impact of passive humidification on clinical outcomes of mechanically ventilated patients: A meta-analysis of randomized controlled trials. *Crit Care Med* 2007; 35: 2843-51;
- Muscedere J, Rewa O, McKechnie K, Jiang X, Laporta D, Heyland DK. Subglottic secretion drainage for the prevention of ventilator-associated pneumonia: a systematic review and meta-analysis. *Crit Care Med* 2011; 39:1985–1991.
- Delaney A, Gray H, Laupland KB, Zuege DJ. Kinetic bed therapy to prevent nosocomial pneumonia in mechanically ventilated patients: a systematic review and meta-analysis. *Crit Care* 2006; 10:R70;
- Sud S, Friedrich JO, Taccone P, Polli F, Adhikari NK, Latini R, Gattinoni L. Prone ventilation reduces mortality in patients with acute respiratory failure and severe hypoxemia: systematic review and meta-analysis. *Inten Care Med* 2010; 36(4); 585-599.
- Siempos II, Vardakas KZ, Falagas ME. Closed tracheal suction systems for prevention of ventilator-associated pneumonia. *Brit J Anaesthesia*, 2008; 100(3): 299-306.

Table S3: Groups of anti-septic studies [a]

Author & regimen	Year	Ref	Notes	MV	LOS	Patients	VAP			Bacteremia		
				%	d	n	v_ps_n	v_ac_n	v_can_n	b_ps_n	b_ac_n	b_can_n
Bellissimo-Rodrigues	2014	195		76	>10	127			1			
Bellissimo-Rodrigues Chlx	2014	195		77	>10	127			0			
Bleasdale	2007	196		35	<5	445				0		1
Bleasdale Chlx	2007	196		36	<5	391				0		0
Cabov	2010	197		57	5to10	30	4	0		0	0	
Cabov	2010	197		77	5to10	30	0	0		0	0	
Caruso	2009	198		100	>10	132	9	5	3			
Caruso Sal	2009	198		100	>10	130	7	1	0			
Climo	2013	199		NS	5to10	1398				2	2	16
Climo Chlx(BW)	2013	199		NS	5to10	1410				4	1	7
Fourrier	2000	200		100	>10	30	4	2	1	0	0	0
Fourrier Chlx	2000	200		100	>10	30	1	2	0	0	1	0
Fourrier	2005	201		100	>10	114	5	0	0	0	0	0
Fourrier Chlx	2005	201		100	>10	114	6	1	0	1	0	0
Huang_2pre	2013	61		NS	<5	15218				8	5	56
Huang_3pre	2013	61		NS	<5	17356				11	10	59
Huang_2TD	2013	61		NS	<5	24752				13	2	63
Huang_3UD	2013	61		NS	<5	26024				14	3	62
Koeman	2006	202		100	>10	130	4		1			
Koeman -Chlx	2006	202		100	>10	127	0		3			
Koeman ChlxC	2006	202		100	>10	128	2		4			
Kollef	2006	203		100	>10	347	9	2	6			
Kollef Iseganan	2006	203		100	>10	362	8	2	0			
Lorente	2012	204		100	5to10	219	5	0	0			
Lorente Chlx	2012	204		100	5to10	217	5	2	0			
Mori	2006	205		100	5to10	414	9	0	2			
Mori PVI	2006	205		100	5to10	1248	10	2	1			
Noto	2015	206		NS	<5	4852				2	0	6
Noto Chlx(BW)	2015	206		NS	<5	4488				4	0	2
Seguin	2006	207		100	>10	31	0					
Seguin	2006	207		100	>10	31	1					
Seguin-PVI	2006	207	T	100	>10	36	0					

Table S3 (continued): Groups of anti-septic studies [a]

Author & regimen	Year	Ref	Notes	MV	LOS	Patients	VAP			Bacteremia		
				%	d	n	v_ps_n	v_ac_n	v_can_n	b_ps_n	b_ac_n	b_can_n
Seguin	2014	208		100	>10	72	1					
Seguin-PVI	2014	208		100	>10	78	3					
Swan	2016	209		57	5to10	164	1	0	1	0	0	
Swan Chlx(BW)	2016	209		69	5to10	161	1	1	0	0	0	
Wittekamp Chlx	2018	210		100	5to10	2108				16		22

Table S3: Footnotes

Notes; T = Data originating from a study for which the majority of ICU admission were for trauma; C = cardio-thoracic ICU; I = Infection control intervention to entire ICU; crf = group wide candidemia risk factor

MV = percentage of group receiving mechanical ventilation; NS – Not stated; LOS is mean or median length of ICU stay; The ICU-LOS is the ICU length of stay. This is based on surrogate measures including mean (or median) length of MV were taken if the length of ICU LOS was not available in order to generate broad categories of ICU stay of <5 days, 5 to 10 days and >10 days

v_ps_n is the count of *Pseudomonas* VAP; v_sr_n is the count of *Staphylococcus aureus* VAP; v_ac_n is the count of *Acinetobacter* VAP and v_can_n is the count of *Candida* isolates from patients with VAP.

b_ps_n is the count of *Pseudomonas* bacteremia; b_sr_n is the count of *Staphylococcus aureus* bacteremia; and b_ac_n is the count of *Acinetobacter* bacteremia; b_can_n is the count of Candidemia; b_cns_n is the count of coagulative negative *Staphylococcus* bacteremia and b_ent_n is the count of *Enterococcal* bacteremia.

Several (n = 5) of these studies were cited in the following source meta-analyses.

- Chan EY, Ruest A, Meade MO, Cook DJ: Oral decontamination for prevention of pneumonia in mechanically ventilated adults: systematic review and meta-analysis. *BMJ* 2007, 334:889–900.
- Labeau SO, Van de Vyver K, Brusselaers N, Vogelaers D, Blot SI: Prevention of ventilator-associated pneumonia with oral antiseptics: a systematic review and meta-analysis. *Lancet Infect Dis* 2011, 11:845-854.
- Pileggi C, Bianco A, Flotta D, *et al*. Prevention of ventilator-associated pneumonia, mortality and all intensive care unit acquired infections by topically applied antimicrobial or antiseptic agents: a meta-analysis of randomized controlled trials in intensive care units. *Crit Care.* 2011; **15**: R155.
- Price R, MacLennan G, Glen J. Selective digestive or oropharyngeal decontamination and topical oropharyngeal chlorhexidine for prevention of death in general intensive care: systematic review and network meta-analysis. *BMJ*. 2014; **348**: g2197.
- Klompas M, Speck K, Howell MD, *et al*. Reappraisal of routine oral care with chlorhexidine gluconate for patients receiving mechanical ventilation: systematic review and meta-analysis. *JAMA Intern Med.* 2014; **174**: 751-61.

Intervention regimens abbreviations

Chlx = chlorhexidine; Chlx BW = chlorhexidine body wash; ChC = chlorhexidine and colisitn; TD = targetted decolonization; UD = universal decolonization; PVI = povidone iodine; CC = concurrent control; SC = saline control; iseganan, is a synthetic variant of a porcine protegrin, which is a natural antibiotic peptide released by neutrophils in response to invasion by microbes [Kollef 2006].

Table S4: Groups of studies of topical antibiotics [a]

Author & regimen	Year	Ref	Notes	MV	LOS	Patients	VAP			Bacteremia		
				%	d	n	v_ps_n	v_ac_n	v_can_n	b_ps_n	b_ac_n	b_can_n
Groups from NCC studies												
Bergmans	2001	211		100	>10	61	5		1			
Bonten	1994	212		91	>10	54	4					
Camus	2014	213		28	<5	925				0	1	2
Camus PTA	2014	213		28	<5	1022				3	0	1
de Smet	2009	214		88	5-10	1990				36		16
de Smet PTA-Ctx	2009	214		93	5-10	2045				16		8
de Smet PTA	2009	214		94	5-10	1904				17		14
Frencken PTA-Ctx	2018	215		100	5-10	1874				15		
Garbino PNeV	2002	216		100	5-10	204	4		2			10
Godard	1990	217		80	>10	84	5					2
Godard PT	1990	217		81	>10	97	0					0
Hartenauer Ctx	1991	218		100	>10	101	20	4		1	1	
Hartenauer PTA-Ctx	1991	218		100	>10	99	0	0		1	0	
Hjortrup CefTMyco	1997	219	crf	100	NS	150			11	1	0	4
Konrad	1989	220		100	NS	83	9					
Konrad PTA-Ctx	1989	220		100	NS	82	2					
Landelle	2018	221		100	5-10	291	9	0				
Landelle PTNy	2018	221		100	5-10	413	12	1				
Landelle PTNy	2018	221		100	5-10	356	2	0				
Ledingham	1988	222		60	5-10	161	3					
Ledingham PTA-Ctx	1988	222		60	5-10	163	2					
Leone PTA-Cef	2002	223		100	>10	324	3	5				
Nardi	1990	224		100	>10	50		7				
Nardi PTA	1990	224		100	>10	47		4				
Nardi PTA	2001	225	T	100	>10	104	4	1	1			1
Nardi PTAM	2001	225	T	100	>10	119	3	0	0			0
Ong PTA-Ctx	2015	226		87	5-10	3080				10		17
Oostdijk PTA-Ctx	2014	227		51	5-10	5483				20	1	26
Oostdijk PTA	2014	227		52	5-10	5508				23	3	55
Rouby E	1994	228		100	>10	251	26					
Rouby P	1994	228		100	>10	347	12					

Table S4 (continued): Groups of studies of topical antibiotics [a]

Author & regimen	Year	Ref	Notes	MV	LOS	Patients	VAP			Bacteremia		
				%	d	n	v_ps_n	v_ac_n	v_can_n	b_ps_n	b_ac_n	b_can_n
Silvestri PTA-Ctx	1999	229		100	5to10	117	2		0	0		0
Steffen PTNy-Ctx	1994	230	crf	100	>10	127				1	0	0
Stoutenbeek	1984	231		100	>10	59	5	1	0	1	2	1
Stoutenbeek PTA-Ctx	1984	231		100	>10	63	3	1	0	0	0	0
Stoutenbeek	1987	232		100	>10	59	5	1	0			
Stoutenbeek PTA	1987	232		100	>10	42	1	2	0	1	0	
Stoutenbeek PTA-Ctx	1987	232		100	>10	63	0	0	0	0	0	0
Valles	2013	233		100	>10	58	2	0				
Valles Ctx	2013	233		100	5to10	71	1	0				
Veelo PTA-Ctx	2008	234		100	>10	231	2					
Winter	1992	235		92	5to10	84	2	0	0			0
Wittekamp	2018	210		100	5to10	2251				21		15
Wittekamp PTNy	2018	210		100	5to10	2224				15		23
Wittekamp PTNy	2018	210		100	>10	2082				9		18
Groups from CC studies												
Abele-Horn	1997	236	T	100	>10	30	3	0				
Abele-Horn PTA-Ctx	1997	236	T	100	>10	58	2	0				
Acquarolo	2005	236		100	>10	19	2	0	0	0		
Acquarolo Ampsul	2005	237		100	>10	19	2	0	0	0		
Aerdts	1991	238		100	>10	39	10	3	2			1
Aerdts PNoA-Ctx	1991	238		100	>10	17	0	0	0			0
Bergmans	2001	212		100	>10	78	8		3			
Bergmans PGV	2001	212		100	>10	87	3		1			
Bion	1991	239	crf	50	<5	31	3	0	2	0	0	0
Bion PTA-Ctx	1991	239	crf	50	<5	21	0	0	3	2	0	0
Blair	1991	240		93	5to10	130	9		1	3		0
Blair PTA-Ctx	1991	240		93	5to10	126	1		0	1		1
Blaise	1994	240	crf	33	5to10	45				1		
Blaise Ofl	1994	240	crf	37	5to10	46				1		

Table S4 (continued): Groups of studies of topical antibiotics [a]

Author & regimen	Year	Ref	Notes	MV	LOS	Patients	VAP			Bacteremia		
				%	d	n	v_ps_n	v_ac_n	v_can_n	b_ps_n	b_ac_n	b_can_n
Bonten	1994	213		86	5-10	21	0					
Bonten PTA	1994	213		100	>10	22	0					
Bouza	2013	242	C	100	>10	38	3		0			
Bouza Lnz-Mrp	2013	242	C	100	5-10	40	3		0			
Camus	2005	243		100	>10	126						
Camus MCh	2005	243		100	5-10	130						
Camus PT	2005	243		100	>10	130						
Camus PT&MCh	2005	243		100	>10	129						
Cerra	1992	244		26	>10	21				1	0	4
Cerra NoNy	1992	244		18	>10	25				1	0	1
Cockerill	1992	245		85	>10	75	4					2
Cockerill PGNy-Ctx	1992	245		85	5-10	75	1					1
de la Cal	2005	246	T	80	>10	54	7	5	0	7	3	0
de la Cal PTA-Ctx	2005	246	T	74	>10	53	2	6	2	9	6	1
Ferrer Ctx	1994	247		100	>10	41	4	0	2	0	0	0
Ferrer PTA-Ctx	1994	247		100	>10	39	1	0	0	1	0	0
Flaherty	1990	248	C	40	<5	56						0
Flaherty PGA-Ctx	1990	248	C	40	<5	51						0
Garbino_ PNeV	2004	249		100	>10	71			1			3
Garbino_ PNeV	2004	249	crf	100	>10	29			0			2
Gaussorgues	1991	250		100	NS	59			1			1
Gaussorgues PGA	1991	250		100	NS	59			0			0
Georges	1994	251		100	5-10	33		0	0			0
Georges PNeA	1994	251		100	5-10	31		1	0			0
Hammond Ctx	1993	252		100	>10	20		1				
Hammond PTA-Ctx	1993	252		100	>10	13		0				
Hammond Ctx	1994	253		100	>10	33	0	0				
Hammond PTA-Ctx	1994	253		100	>10	39	0	1				
Hellinger	2002	254	crf	NS	>10	43						0
Hellinger PGNy	2002	254	crf	NS	>10	37						0

Table S4 (continued): Groups of studies of topical antibiotics [a]

Author & regimen	Year	Ref	Notes	MV	LOS	Patients	VAP			Bacteremia		
				%	d	n	v_ps_n	v_ac_n	v_can_n	b_ps_n	b_ac_n	b_can_n
Jacobs	1992	255		100	5-10	43	0	0	0	0	0	0
Jacobs PTA-Ctx	1992	255		100	5-10	36	0	0	0	0	1	0
Karvouniaris	2015	256		100	>10	84		11				
Karvouniaris P	2015	256		100	>10	84		2				
Kerver	1988	257		100	>10	47			0			0
Kerver PTA-Ctx	1988	257		100	>10	49			0			0
Korinek	1993	258		100	>10	60	3	1	0			
Korinek PTA-V	1993	258		100	>10	63	0	1	0			
Laggner	1994	259		100	>10	34	1		0	1	0	0
Laggner GA	1994	259		100	>10	33	0		0	0	0	0
Langlois -Karaga	1995	260		100	>10	50						
Langlois -Karaga PGA	1995	260		100	>10	47						
Palomar	1997	261		100	5-10	42	6	0	0			0
Palomar PTA-Ctx	1997	261		100	5-10	41	1	0	0			0
Palomar Ctx	1997	261		100	>10	46	1	2				1
Pneumatikos	2002	262	T	100	>10	30	1	1	0			
Pneumatikos PTA	2002	262	T	100	>10	31	0	1	0			
Quinio	1995	263	T	100	>10	72	12		0	0	0	0
Quinio PGA	1995	263	T	100	>10	76	5		0	0	0	0
Rimola	1985	264	crf	NS	5-10	72				0	0	
Rimola PGVNy	1985	264	crf	NS	>10	68				0	0	
Rocha	1992	265	T	100	>10	54	8	4		1	3	0
Rocha PTA-Ctx	1992	265	T	100	>10	47	1	1		0	0	0
Rodríguez -Roldán	1990	266		100	>10	15	5	2				
Rodríguez -Roldán PTNeA	1990	266		100	5-10	14	0	0				
Rolando	1996	267	crf	73	NS	61	2	0	1	0	0	1
Rolando PTA	1996	267	crf	73	NS	47	1	0	1	0	0	0
Rolando	1993	268	crf	75	5-10	31	2	0	3			0
Rolando PTAM-Cfu	1993	268	crf	75	5-10	28	1	0	0			0

Table S4 (continued): Groups of studies of topical antibiotics [a]

Author & regimen	Year	Ref	Notes	MV	LOS	Patients	VAP			Bacteremia		
				%	d	n	v_ps_n	v_ac_n	v_can_n	b_ps_n	b_ac_n	b_can_n
Sanchez-Garcia	1998	269		100	>10	140			0			
Sanchez-Garcia PTA-Ctx	1998	269		100	>10	131			0			
Sirvent	1997	270		100	>10	50	1	2	0			
Sirvent -Cef	1997	270		100	>10	50	3	1	0			
Smith	1993	271	crf	100	5to10	18	1		1			1
Smith PTA-Ctx	1993	271	crf	100	5to10	18	0		0			0
Stoutenbeek	2007	272	T	100	>10	200	28	23	21	2	3	0
Stoutenbeek PTA-Ctx	2007	272	T	100	>10	201	11	15	6	3	0	1
Ulrich	1989	273		83	>10	52			1			0
Ulrich_ PNoA-Tr	1989	273		77	>10	48			1			0
Unertl	1987	274		100	>10	20	2		0			
Unertl_PGA	1987	274		100	>10	19	0		0			
van Delden	2012	275		100	>10	45	6					
van Delden -Azith	2012	275		100	5to10	47	2					
Verwaest	1997	276		100	>10	185	7	4	0	1	2	4
Verwaest OfA-Of	1997	276		100	>10	193	2	4	1	2	2	0
Verwaest PTA-Ctx	1997	276		100	>10	200	10	4	0	6	2	0
Wiener	1995	277		100	>10	31	0	0	0			2
Wiener_ PGNy	1995	277		100	>10	30	2	1	0			0
Winter	1992	235		92	5to10	92	8	2	0			1
Winter_ PTA-Ctz	1992	235		92	5to10	91	3	0	0			0

Table S4: Footnotes

Notes; T = Data originating from a study for which the majority of ICU admission were for trauma; C = cardio-thoracic ICU; I = Infection control intervention to entire ICU; crf = group wide candidemia risk factor

MV = percentage of group receiving mechanical ventilation; NS – Not stated; LOS is mean or median length of ICU stay; The ICU-LOS is the ICU length of stay. This is based on surrogate measures including mean (or median) length of MV were taken if the length of ICU LOS was not available in order to generate broad categories of ICU stay of <5 days, 5 to 10 days and >10 days

v_ps_n is the count of *Pseudomonas* VAP; v_sr_n is the count of *Staphylococcus aureus* VAP; v_ac_n is the count of *Acinetobacter* VAP and v_can_n is the count of *Candida* isolates from patients with VAP.

b_ps_n is the count of *Pseudomonas* bacteremia; b_sr_n is the count of *Staphylococcus aureus* bacteremia; and b_ac_n is the count of *Acinetobacter* bacteremia; b_can_n is the count of Candidemia; b_cns_n is the count of coagulative negative *Staphylococcus* bacteremia and b_ent_n is the count of *Enterococcal* bacteremia.

* De Smet 2009 [214] The *Pseudomonas* and *Acinetobacter* counts in this study are available only as a combined category count for gram negative non-fermentative GNB and are provided here for reference but are not used in the analysis. The Pseudomonas counts in the study of Oostdijk [227] includes counts from the study by De Smet 2009 [214]. The Pseudomonas counts in the study of Oostdijk [227] are taken as given in Table 2 of Oostdijk [227] even though this Table appears to be mislabelled in comparison to the text and table 1 of Oostdijk [227] and in relation to the data in De Smet 2009 [214].

The control group in one study by Stoutenbeek [1987] appears also as the control group in another study by this Author [1984] and is used only once in the analysis here.

Several (n = 24) of these studies were cited in the following source systematic reviews.

- Liberati A, D'Amico R, Pifferi S, Torri V, Brazzi L, Parmelli E: Antibiotic prophylaxis to reduce respiratory tract infections and mortality in adults receiving intensive care. *Cochrane Database Syst Rev* 2009, 4.
- Pileggi C, Bianco A, Flotta D, Nobile CG, Pavia M. Prevention of ventilator-associated pneumonia, mortality and all intensive care unit acquired infections by topically applied antimicrobial or antiseptic agents: a meta-analysis of randomized controlled trials in intensive care units. *Crit Care* 2011; 15:R155.
- Silvestri L, Van Saene HK, Milanese M, Gregori D. Impact of selective decontamination of the digestive tract on fungal carriage and infection: systematic review of randomized controlled trials. *Intensive Care Med* 2005, 31:898-910.
- Chan EY, Ruest A, Meade MO, Cook DJ. Oral decontamination for prevention of pneumonia in mechanically ventilated adults: systematic review and meta-analysis. *BMJ.* 2007; 334:889–900.

TAP intervention regimens abbrevations; PTA (=P, topical polymyxin; T, topical tobramycin; A, topical amphotericin); PTA-Ctx (=P, topical polymyxin; T, topical tobramycin; A, topical amphotericin; Ctx, parenteral cephalosporin); P (P = polymyxin either aerosolized or topical); PNeV (P = polymyxin; Ne = Neomycin; V = Vancomycin); PGA-Ctx (=P, topical polymyxin; G, topical gentamicin; A, topical amphotericin; Ctx, parenteral cephalosporin); PTAM (=P, topical polymyxin; T, topical tobramycin; A, topical amphotericin; topical mupirocin); E (=E, topical erythromycin); PNoA-Ctx (=P, topical polymyxin; No, topical norfloxacin; A, topical amphotericin; Ctx, parenteral cephalsporin); PGV (=P, topical polymyxin; G, topical gentamicin; V, topical vancomycin); PGNy-Ctx (=P, topical polymyxin; G, topical gentamicin; Ny, topical nystatin; Ctx, parenteral cephalsporin); P-Ctx (=P, topical polymyxin; Ctx, parenteral cephalosporin); PTAV (=P, topical polymyxin; T, topical tobramycin; A, topical amphotericin; V, topical vancomycin); PGA (=P, topical polymyxin; G, topical gentamicin; A, topical amphotericin); PTNeA (=P, topical polymyxin; T, topical tobramycin; Ne, topical Neomycin; A, topical amphotericin); PGNy (=P, topical polymyxin; G, topical gentamicin; Ny, topical nystatin); PTA-Cz (=P, topical polymyxin; T, topical tobramycin; A, topical amphotericin; Cz, parenteral Ceftazidime).

Table S5: Groups of studies of antifungal prophylaxis

Author & regimen	Year	Ref	Notes	MV	LOS	Patients	RT Candida	Candidiemia
				%	d	n	v_can_n	b_can_n
Ables	2000	278	crf	95	5to10	60		0
Ables_Fluc	2000	278	crf	95	5to10	59		0
Eggimann	1999	279	crf	NS	>10	20		2
Eggimann_Fluc	1999	279	crf	NS	>10	23		0
Giglio	2012	280		100	>10	50	0	0
Giglio_Ny	2012	280		100	>10	49	0	0
Jacobs	2003	281		NS	>10	39		1
Jacobs_Fluc	2003	281		NS	>10	32		0
Lumbreras	1996	282	crf	NS	>10	67		0
Lumbreras_Fluc	1996	282	crf	NS	>10	76		1
Normand	2005	283		100	>10	47	0	0
Normand_Ny	2005	283		100	>10	51	0	0
Ostrosky-Zeichner	2014	284	crf	100	5to10	102		7
Ostrosky-Zeichner_ Casp	2014	284		100	5to10	117		1
Savino	1994	285		31	>10	72		0
Savino _Cl_ket_ny	1994	285		23	>10	80		0
Savino	1994	285	crf	33	5to10	65		2
Savino _Cl_ket_ny	1994	285	crf	39	>10	75		7
Schuster	2008	286		NS	>10	122		2
Schuster_Fluc	2008	286		NS	>10	122		0

Table S5: FootNotes

Notes; T = Data originating from a study for which the majority of ICU admission were for trauma; C = cardio-thoracic ICU; I = Infection control intervention to entire ICU; crf = group wide candidemia risk factor

MV = percentage of group receiving mechanical ventilation; NS – Not stated; LOS is mean or median length of ICU stay; The ICU-LOS is the ICU length of stay. This is based on surrogate measures including mean (or median) length of MV were taken if the length of ICU LOS was not available in order to generate broad categories of ICU stay of <5 days, 5 to 10 days and >10 days

RT Candida is respiratory tract candida. v_can_n is the count of *Candida* isolates from patients with VAP; b_can_n is the count of Candidemia;

Table S6: Development of Pseudomonas GSEM model [a, b]

	Full models (all studies)			Models excluding studies ICU-LOS<5 days		
	**Model 1**	**Model 2**		**Model 3**	**Model 4 .**	
	Fig S3a	Fig S3b		Fig S4a	Fig S4a	
			95%CI			95%CI
Factor [c - k]						
b_Ps_n						
Pseudomonas colonization	1.09***	1.10***	0.71 to 1.5	1.30***	1.28***	0.84 to 1.7
ppap	0.85**	0.83**	0.25 to 1.4	0.98**	0.93**	0.31 to 1.7
_cons	-5.96***	-5.93***	-6.8 to -5.1	-5.16***	-5.05***	-5.5 to -4.5
v_Ps_n						
Pseudomonas colonization	1	1	(constrained)	1	1	(constrained)
mvp90	0.36	0.32	-0.04 to 0.7	0.42*	0.39*	0.02 to 0.76
non_D	-0.50***	-0.49***	-0.76 to -0.2	-0.48***	-0.46***	-0.73 to -0.2
_cons	-4.33***	-4.31***	-5.1 to -3.5	-3.51***	-3.45***	-3.9to -3.0
Pseudomonas colonization						
tap	-0.71***	-0.45***	-0.71 to -0.2	-0.64***	-0.39**	-0.66 to -0.12
year	-0.15*	-0.18**	-0.31 to -0.1	-0.14*	-0.18**	-0.3 to -0.05
los5	0.99*	0.51	-0.20 to 1.2			
los10	0.51***	0.52***	0.28 to 0.7	0.60***	0.54***	0.32 to 0.76
trauma	-0.14	-0.13	-0.47 to 0.2	-0.14	-0.12	-0.48 to 0.2
Anti-septic	-0.77***	-0.28	-0.77 to 0.2	-0.84***	-0.27	-0.75 to 0.22
crf	0.26	-0.46	-1.2 to 0.2	0.18	-0.54	-1.1 to 0.05
Candida colonization	-	0.33***	0.22 to 0.45		0.36***	0.24 to 0.48
b_can_n						
Candida colonization	0.50***	0.48***	0.3 to 0.64	0.58***	0.54***	0.36 to 0.73
_cons	-5.62***	-5.66***	-6.2 to -5.1	-5.02***	-5.09***	-5.6 to -4.6
v_can_n						
Candida colonization	1	1	(constrained)	1	1	(constrained)
mvp90	0.02	0.31	-0.53 to 1.1	0.2	0.3	-0.4 to 1.3
non_D	-0.28	-0.43	-1.1 to 0.21	-0.22	-0.21	-1.0 to 0.28
_cons	-6.53***	-6.86***	-8.4 to -5.3	-5.26***	-5.29***	-6.7 to -4.5

Table S6: Development of Pseudomonas GSEM model (continued) [a, b]

Full models (all studies)					Models excluding studies ICU-LOS<5 days			
	**Model 1**	**Model 2**				**Model 3**	**Model 4 .**	
	Fig S3a	Fig S3b				Fig S4a	Fig S4a	
			95%CI					95%CI
Factor [c - k]								
Candida colonization								
tap	0.64	0.54	-0.31 to 1.4			0.62	0.62	-0.31 to 1.3
year	-0.01	0.08	-0.26 to 0.4			-0.03	-0.04	-0.23 to 0.37
los5	1.62**	1.59**	0.62 to 2.7					
los10	-0.09	-0.13	-0.59 to 0.4			0.17	0.16	-0.39 to 0.6
trauma	0.19	0.05	-0.9 to 0.9			0.19	0.16	-0.8 to 0.9
Anti-septic	-1.44**	-1.47**	-2.4 to -0.6			-1.66**	-1.59**	-2.6 to -0.77
Anti-fungal	-1.58***	-1.6***	-2.5 to -0.72			-1.43***	-1.46***	-2.3 to -0.63
crf	2.10***	2.25***	1.2 to 3.3			2.11***	2.17***	1.3 to 3.2
Error terms								
var (e. Candida colonization)	1.49***	1.49***	1.0 to 2.3			1.52***	1.53***	1.0 to 2.3
var (e. Pseudomonas colonization)	0.46***	0.30***	0.20 to 0.44			0.47***	0.29***	0.19 to 0.43
			-					
Model fit [l]								
AIC	3631.39	3600.45	-			3660.34	3622.49	-
Groups (n)	439	439	-			421	421	-
Clusters (n)	276	276				271	271	-
Factors	30	31	-			28	29	-

Footnotes

a. Legend: * p<0.05; ** p<0.01; *** p<0.001
b. Shown in this table are models derived with all studies including those with LOS<5 days (models 1, & 2) and models derived after excluding studies with LOS< 5 days (models 3 & 4). The figures corresponding to models 1 (Figure S5a), model 2 (Figure 1 & figure S5b) models 3 (Figure S6a), model 4 (figure S6b).

c. v_ps_n is the count of *Pseudomonas* VAP; v_ac_n is the count of *Acinetobacter* VAP; v_can_n is the count of *RT Candida*; b_ps_n is the count of *Pseudomonas* bacteremia and b_ac_n is the count of *Acinetobacter* bacteremia; b_can_n is the count of Candidemia.

d. PPAP is the group wide use of protocolized parenteral antibiotic prophylaxis; tap is topical antibiotic prophylaxis; non-D is a non-decontamination intervention; year = year of study publication in units of ten (decade)

e. MVP90 is use of mechanical ventilation by more than 90% of the group

f. Crf is group wide exposure to a candidemia risk factor.

g. LOS5 is a mean or median length of ICU stay for the group of less than 5 days

h. LOS10 is a mean or median length of ICU stay for the group of more than 10 days

i. Trauma ICU arbitrarily defined as an ICU for which >50% of admissions were for trauma

j. Pseudomonas colonization (Pseudomonas col) is a latent variable

k. *Candida* colonization (*Candida* col) is a latent variable

l. Model fit; AIC is Akaike's information criteria. This indicates model fit taking into account the statistical goodness of fit and the number of parameters in the model. Lower values of AIC indicate a better model fit. Groups is the number of patient groups; clusters is the number of studies; N is the number of parameters in the model.

Table S7: Development of Acinetobacter GSEM model [a, b]

	Full models (all studies)			Models excluding studies ICU-LOS<5 days		
	Model 5	Model 6 .		Model 7	Model 8	
	Fig S5a	Fig S5b		Fig S6a	Fig S7a	
			95%CI			95%CI
Factor [c - k]						
b_Ac_n						
Acinetobacter colonization	1.18***	1.17***	0.92 to 1.4	1.25***	1.23***	0.92 to 1.4
ppap	0.24	0.17	-0.87 to 1.4	0.31	0.21	-0.87 to 1.4
_cons	-8.14***	-8.08***	-9.2 to -6.9	-7.60***	-7.42***	-9.2 to -6.9
v_Ac_n						
Acinetobacter colonization	1	1	(constrained)	1	1	(constrained)
mvp90	0.58	0.54	-0.12 to 1.3	0.6	0.57	-0.12 to 1.3
non_D	-0.41	-0.37	-0.76 to 0.05	-0.39	-0.34	-0.76 to 0.05
_cons	-6.56***	-6.56***	-7.8 to -5.3	-5.92***	-5.85***	-7.8 to -5.3
Acinetobacter colonization						
tap	-0.69**	-0.43	-1.1 to 0.0	-0.66*	-0.4	-1.1 to 0.0
year	0.17	0.17	-0.12 to 0.4	0.16	0.17	-0.12 to 0.4
los5	0.76	0.34	-0.4 to 1.3			
los10	0.94***	0.91***	0.46 to 1.3	1.05***	0.95***	0.46 to 1.3
trauma	0.44	0.4	-0.03 to 0.95	0.45	0.4	-0.03 to 0.95
Anti-septic	-1.09**	-0.59	-1.5 to 0.1	-1.16**	-0.55	-1.5 to 0.1
crf	-0.27	-.095	-3.1 to 1.2	-0.29	-1.05	-3.1 to 1.2
Candida colonization	-	0.32*	0.01 to 0.5		0.36**	0.01 to 0.5
b_can_n						
Candida colonization	0.49***	0.49***	0.3 to 0.67	0.49***	0.49***	0.3 to 0.67
_cons	-5.6***	-5.56***	-6.1 to -4.9	-5.62***	-5.56***	-6.1 to -4.9
v_can_n						
Candida colonization	1	1	(constrained)	1	1	(constrained)
mvp90	0.02	0.12	-0.80 to 0.89	0.2	0.3	-0.80 to 0.89
non_D	-0.28	-0.27	-0.97 to 0.38	-0.22	-0.21	-0.97 to 0.38
_cons	-6.5***	-6.51***	-8.1 to -4.9	-5.26***	-5.29***	-8.1 to -4.9

Table S7: Development of Acinetobacter GSEM model (continued) [a, b]

Full models (all studies)					Models excluding studies ICU-LOS<5 days			
	Model 5	Model 6				Model 7	Model 8	
	Fig S5a	Fig S5b				Fig S6a	Fig S7a	
			95%CI					95%CI
Factor [c - k]								
Candida colonization								
tap	0.64	0.64	-0.22 to 1.5			0.62	0.62	-0.22 to 1.5
year	-0.01	-0.02	-0.36 to 0.28			-0.03	-0.04	-0.36 to 0.28
los5	1.62**	1.55**	0.51 to 2.7					
los10	-0.09	-0.08	-0.55 to 0.5			0.17	0.16	-0.55 to 0.5
trauma	0.19	0.16	-0.83 to 0.99			0.19	0.16	-0.83 to 0.99
Anti-septic	-1.44**	-1.39**	-2.4 to -0.5			-1.66**	-1.59**	-2.4 to -0.5
Anti-fungal	-1.58***	-1.6***	-2.5 to -0.69			-1.43***	-1.46***	-2.5 to -0.69
crf	2.10***	2.16***	1.05 to 3.1			2.11***	2.17***	1.05 to 3.1
Error terms								
var (e. Candida colonization)	1.49***	1.47***	1.0 to 2.3			1.52***	1.50***	1.0 to 2.3
var (e. Acinetobacter colonization)	1.42***	1.27***	0.95 to 1.7			1.42***	1.27***	0.95 to 1.7
Model fit [l]								
AIC	2723.04	2717.31	-			2736.76	2728.7	-
Groups (n)	395	395	-			378	378	-
Clusters (n)	247	247	-			239	239	-
Factors	30	31	-			28	29	-

Table S7: Development of Acinetobacter GSEM model (continued) [a]

Footnotes

a. Legend: * p<0.05; ** p<0.01; *** p<0.001
b. Shown in this table are models derived with all studies including those with LOS<5 days (models 5, & 6) and after excluding studies with LOS< 5 days (models 7 & 8). The figures corresponding to models 5 (Figure S7a), model 6 (figure S7b) models 7 (Figure S8a), model 8 (figure S8b).
c. v_ps_n is the count of *Pseudomonas* VAP; v_ac_n is the count of *Acinetobacter* VAP; v_can_n is the count of *RT Candida*; b_ps_n is the count of *Pseudomonas* bacteremia and b_ac_n is the count of *Acinetobacter* bacteremia; b_can_n is the count of Candidemia.
d. PPAP is the group wide use of protocolized parenteral antibiotic prophylaxis; tap is topical antibiotic prophylaxis; non-D is a non-decontamination intervention; year = year of study publication in units of ten (decade)
e. MVP90 is use of mechanical ventilation by more than 90% of the group
f. Crf is group wide exposure to a candidemia risk factor.
g. LOS5 is a mean or median length of ICU stay for the group of less than 5 days
h. LOS10 is a mean or median length of ICU stay for the group of more than 10 days
i. Trauma ICU arbitrarily defined as an ICU for which >50% of admissions were for trauma
j. Acinetobacter colonization (Acinteobacter col) is a latent variable
k. *Candida* colonization (*Candida* col) is a latent variable
l. Model fit; AIC is Akaike's information criteria. This indicates model fit taking into account the statistical goodness of fit and the number of parameters in the model. Lower values of AIC indicate a better model fit. Groups is the number of patient groups; clusters is the number of studies; N is the number of parameters in the model.

References

S1. A'Court CH, Garrard CS, Crook D, Bowler I, Conlon C, Peto T, Anderson E: Microbiological lung surveillance in mechanically ventilated patients, using non-directed bronchial lavage and quantitative culture. Q J Med. 1993;86:635-48.

S2. Alvarez-Lerma F, ICU-acquired Pneumonia Study Group. Modification of empiric antibiotic treatment in patients with pneumonia acquired in the intensive care unit. *Intens Care Med.* 1996;22(5):387-94.

S3. Antonelli M, Moro ML, Capelli O, De Blasi RA, D'Errico RR, Conti G, Bufi M, Gasparetto A: Risk factors for early onset pneumonia in trauma patients. Chest. 1994;105:224-228

S4. Apostolopoulou E, Bakakos P, Katostaras T, Gregorakos L: Incidence and risk factors for ventilator-associated pneumonia in 4 multidisciplinary intensive care units in Athens, Greece. Respir Care. 2003;48: 681-688.

S5. Arumugam SK, Mudali I, Strandvik G, El-Menyar A, Al-Hassani A, Al-Thani H. Risk factors for ventilator-associated pneumonia in trauma patients: A descriptive analysis. World J Emerg Med. 2018;9(3):203.

S6. Azoulay E, Timsit JF, Tafflet M, de Lassence A, Darmon M, Zahar JR, Schlemmer B. *Candida* colonization of the respiratory tract and subsequent pseudomonas ventilator-associated pneumonia. *Chest* 2006;129(1):110-117.

S7. Bailly S, Bouadma L, Azoulay E, Orgeas MG, Adrie C, Souweine B, Schwebel C, Maubon D, Hamidfar-Roy R, Darmon M, Wolff M. Failure of empirical systemic antifungal therapy in mechanically ventilated critically ill patients. *Am J Respir Crit Care Med.* 2015;191(10):1139-46.

S8. Baraibar J, Correa H, Mariscal D, Gallego M, Valles J, Rello J. Risk factors for infection by *Acinetobacter baumannii* in intubated patients with nosocomial pneumonia. *Chest.* 1997; 112:1050-1054.

S9. Beck-Sague CM, Sinkowitz RL, Chinn RY, Vargo J, Kaler W, Jarvis WR: Risk factors for ventilator-associated pneumonia in surgical intensive-care-unit patients. Infect Control Hosp Epidemiol. 1996;17:374-6.

S10. Bekaert M, Timsit JF, Vansteelandt S, Depuydt P, Vésin A, Garrouste-Orgeas M, Decruyenaere J, Clec'h C, Azoulay E, Benoit D. Attributable mortality of ventilator-associated pneumonia: a reappraisal using causal analysis. *Am J Respir Crit Care Med*. 2011;184(10):1133-9.

S11. Bercault N, Boulain T: Mortality rate attributable to ventilator-associated nosocomial pneumonia in an adult intensive care unit: a prospective case-control study. Crit Care Med. 2001;29:2303-2309

S12. Bercault N, Wolf M, Runge I, et al. Intrahospital transport of critically ill ventilated patients: a risk factor for ventilator-associated pneumonia--a matched cohort study. *Crit Care Med* 2005;33:2471-8.

S13. Berrouane Y, Daudenthun I, Riegel B, Emery MN, Martin G, Krivosic R, Grandbastien B. Early onset pneumonia in neurosurgical intensive care unit patients. J Hosp Infect. 1998;40(4):275-80.

S14. Blot S, Koulenti D, Dimopoulos G, Martin C, Komnos A, Krueger WA, Spina G, Armaganidis A, Rello J. Prevalence, risk factors, and mortality for ventilator-associated pneumonia in middle-aged, old, and very old critically ill patients. Crit Care Med. 2014;42(3):601-9.

S15. Bochicchio GV, Joshi M, Bochicchio K, Tracy K, Scalea TM: A time-dependent analysis of intensive care unit pneumonia in trauma patients. J Trauma. 2004;56:296-301.

S16. Bonten MJ, Gaillard CA, van Tiel FH, Smeets HG, van der Geest S, Stobberingh EE: The stomach is not a source for colonization of the upper respiratory tract and pneumonia in ICU patients. Chest. 1994;105(3):878-84.

S17. Bonten MJ, Bergmans DC, Ambergen AW, De Leeuw PW, Van der Geest S, Stobberingh EE, Gaillard CA. Risk factors for pneumonia, and colonization of respiratory tract and stomach in mechanically ventilated ICU patients. *Am J Respir Crit Care Med* 1996;154(5):1339-1346.

S18. Boots RJ, Phillips GE, George N, Faoagali JL. Surveillance culture utility and safety using low-volume blind bronchoalveolar lavage in the diagnosis of ventilator-associated pneumonia. Respirology. 2008;13:87-96.

S19. Bornstain C, Azoulay E, De Lassence A, Cohen Y, Costa MA, Mourvillier B, Descorps-Declere A, Garrouste-Orgeas M, Thuong M, Schlemmer B, Timsit JF: Sedation, sucralfate, and antibiotic use are potential means for protection against early-onset ventilator-associated pneumonia. Clin Infect Dis. 2004;38(10):1401-8.

S20. Borzotta AP, Beardsley K (1999) Candida infections in critically ill trauma patients: a retrospective case-control study. Arch Surg 134(6):657-665

S21. Braun SR, Levin AB, Clark KL. Role of corticosteroids in the development of pneumonia in mechanically ventilated head-trauma victims. Crit Care Med 1986;14:198-201

S22. Bregeon F, Papazian L, Visconti A, Gregoire R, Thirion X, Gouin F: Relationship of microbiologic diagnostic criteria to morbidity and mortality in patients with ventilator-associated pneumonia. JAMA. 1997;277: 655-662

S23. Bronchard R, Albaladejo P, Brezac G, et al. Early onset pneumonia: risk factors and consequences in head trauma patients. *Anesthesiology* 2004;100:234-9.

S24. Cade JF, McOwat E, Siganporia R, Keighley C, Presneill J, Sinickas V: Uncertain relevance of gastric colonization in the seriously ill. Intensive Care Med. 1992;18:210-217

S25. Cavalcanti M, Ferrer M, Ferrer R, Morforte R, Garnacho A, Torres A: Risk and prognostic factors of ventilator-associated pneumonia in trauma patients. Crit Care Med. 2006;34:1067-1072

S26. Cendrero JA, Solé-Violán J, Benitez AB, Catalán JN, Fernández JA, Santana PS, de Castro FR: Role of different routes of tracheal colonization in the development of pneumonia in patients receiving mechanical ventilation. Chest. 1999;116:462-470

S27. Chaari A, El Habib M, Ghdhoun H, Algia NB, Chtara K, Hamida CB, Chelly H, Bahloul M, Bouaziz M. Does low-dose hydrocortisone therapy prevent ventilator-associated pneumonia in trauma patients?. Am J Therap. 2015;22(1):22-8.

S28. Charles PE, Dalle F, Aube H, Doise JM, Quenot JP, Aho LS, Blettery B (2005) Candida spp. colonization significance in critically ill medical patients: a prospective study. Intensive Care Med 31(3):393-400.

S29. Chastre J, Trouillet JL, Vuagnat A, Joly-Guillou ML, Clavier H, Dombret MC, Gibert C: Nosocomial pneumonia in patients with acute respiratory distress syndrome. *Am J Respir Crit Care Med.* 1998;157:1165-1172

S30. Chevret S, Hemmer M, Carlet J: Incidence and risk factors of pneumonia acquired in intensive care units. Results from a multicenter prospective study on 996 patients. European Cooperative Group on Nosocomial Pneumonia. Intensive Care Med. 1993;19:256-264

S31. Combes P, Fauvage B, Oleyer C. Nosocomial pneumonia in mechanically ventilated patients, a prospective randomised evaluation of the Stericath closed suctioning system. *Intensive Care Med* 2000;26:878-82.

S32. Cook A, Norwood S, Berne J: Ventilator-associated pneumonia is more common and of less consequence in trauma patients compared with other critically ill patients. J Trauma Acute Care Surg. 2010;69(5):1083-91.

S33. Craven DE, Kunches LM, Lichtenberg DA, Kollisch NR, Barry MA, Heeren TC, McCabe WR: Nosocomial infection and fatality in medical and surgical intensive care unit patients. Arch Intern Med. 1988;148:1161-1168

S34. Daschner F, Kappstein I, Schuster F, Scholz R, Bauer E, Jooβens D, Just H: Influence of disposable ('Conchapak') and reusable humidifying systems on the incidence of ventilation pneumonia. J Hosp Infect. 1988;11:161-168

S35. De Waele JJ, Vogelaers D, Blot S Colardyn F (2003) Fungal infections in patients with severe acute pancreatitis and the use of prophylactic therapy. Clin Infect Dis 37:208–213.

S36. De Latorre FJ, Pont T, Ferrer A, Rosselló J, Palomar M, Planas M: Pattern of tracheal colonization during mechanical ventilation. Am J Respir Crit Care Med. 1995;152:1028-1033

S37. De Santis V, Gresoiu M, Corona A, Wilson AP, Singer M. Bacteraemia incidence, causative organisms and resistance patterns, antibiotic strategies and outcomes in a single university hospital ICU: continuing improvement between 2000 and 2013. J Antimicrob Chemoth. 2015;70(1):273-8.

S38. El-Masri MM, Hammad TA, McLeskey SW, Joshi M, Korniewicz DM. Predictors of nosocomial bloodstream infections among critically ill adult trauma patients. *Infect Cont & Hosp Epidemiol.* 2004;25(8):656-63.

S39. Ensminger SA, Wright RS, Baddour LM, Afess B: Suspected ventilator-associated pneumonia in cardiac patients admitted to the coronary care unit. Mayo Clin Proc. 2006;81:32–35

S40. Ertugrul BM, Yildirim A, Ay P, Oncu S, Cagatay A, Cakar N, Ertekin C, Ozsut H, Eraksoy H, Calangu S. Ventilator-associated pneumonia in surgical emergency intensive care unit. Saudi Med J. 2006;27(1):52.

S41. Esteve F, Pujol M, Limon E, Saballs M, Argerich MJ, Verdaguer R, Manez R, Ariza X, Gudiol F. Bloodstream infection related to catheter connections: a prospective trial of two connection systems. *J Hosp Infect*. 2007;67(1):30-4.

S42. Evans HL, Zonies DH, Warner KJ, Bulger EM, Sharar SR, Maier RV, Cuschieri J. Timing of intubation and ventilator-associated pneumonia following injury. Arch Surg. 2010;145(11):1041-6.

S43. Ewig S, Torres A, El-Ebiary M, Fàbregas N, Hernandez C, Gonzalez J, Nicolas JM, Soto L: Bacterial colonization patterns in mechanically ventilated patients with traumatic and medical head injury. Incidence, risk factors, and association with ventilator-associated pneumonia. Am J Respir Crit Care Med. 1999;159:188-198

S44. Fabian TC, Boucher BA, Croce MA, Kuhl DA, Janning SW, Coffey BC, Kudsk KA: Pneumonia and stress ulceration in severely injured patients: a prospective evaluation of the effects of stress ulcer prophylaxis. Arch Surg. 1993;128(2):185-92.

S45. Fagon JY, Chastre J, Domart Y, Trouillet JL, Pierre J, Darne C, Gibert C: Nosocomial pneumonia in patients receiving continuous mechanical ventilation. Prospective analysis of 52 episodes with use of a protected specimen brush and quantitative culture techniques. *Am Rev Respir Dis* 1989;139:877-884.

S46. Ferreira D, Grenouillet F, Blasco G, Samain E, Hénon T, Dussaucy A, Millon L, Mercier M, Pili-Floury S. Outcomes associated with routine systemic antifungal therapy in critically ill patients with Candida colonization. Intens care Med. 2015;41(6):1077-88.

S47. Gacouin A, Barbarot N, Camus C, Salomon S, Isslame S, Marque S, Lavoué S, Donnio PY, Thomas R, Le Tulzo Y. Late-onset ventilator-associated pneumonia in nontrauma intensive care unit patients. Anesth Analg. 2009;109(5):1584-90.

S48. García-Garmendia JL, Ortiz-Leyba C, Garnacho-Montero J, Jiménez-Jiménez FJ, Pérez-Paredes C, Barrero-Almodóvar AE, Miner MG. Risk factors for Acinetobacter baumannii nosocomial bacteremia in critically ill patients: a cohort study. *Clin Infect Dis*. 2001;33(7):939-46.

S49. Garrouste-Orgeas M, Chevret S, Arlet G, Marie O, Rouveau M, Popoff N, Schlemmer B: Oropharyngeal or gastric colonization and nosocomial pneumonia in adult intensive care unit patients. A prospective study based on genomic DNA analysis. Am J Respir Crit Care Med. 1997;156(5):1647-56.

S50. Garrouste-Orgeas M, Timsit JF, Tafflet M, Misset B, Zahar JR, Soufir L, Carlet J: Excess risk of death from intensive care unit—acquired nosocomial bloodstream infections: a reappraisal. *Clin Infect Dis* 2006, 42:1118-1126.

S51. George DL, Falk PS, Wunderink RG, Leeper Jr KV, Meduri GU, Steere EL, Glen Mayhall C: Epidemiology of ventilator-acquired pneumonia based on protected bronchoscopic sampling. Am J Respir Crit Care Med. 1998;158:1839-1847

S52. Georges H, Leroy O, Guery B, Alfandari S, Beaucaire G: Predisposing factors for nosocomial pneumonia in patients receiving mechanical ventilation and requiring tracheotomy. Chest. 2000;118:767–774.

S53. Giamarellos-Bourboulis EJ, Bengmark S, Kannellakopoulou K, Kotzampassi K: Pro- and synbiotics to control inflammation and infection in patients with multiple injuries. *J Trauma* 2009;67:815-821.

S54. Giard M, Lepape A, Allaouchiche B, Guerin C, Lehot JJ, Robert MO, Vanhems P: Early-and late-onset ventilator-associated pneumonia acquired in the intensive care unit: comparison of risk factors. *J Crit Care* 2008, 23:27-33.

S55. Gruson D, Hilbert G, Vargas F, Valentino R, Bebear C, Allery A, Bebear C, Gbikpi-Benissan GE, Cardinaud JP: Rotation and restricted use of antibiotics in a medical intensive care unit: impact on the incidence of ventilator-associated pneumonia caused by antibiotic-resistant gram-negative bacteria. Am J Respir Crit Care Med. 2000, 162(3):837-43.

S56. Gruson D, Hilbert G, Vargas F, Valentino R, Bui N, Pereyre S, Bebear C, Bebear CM, Gbikpi-Benissan G: Strategy of antibiotic rotation: long-term effect on incidence and susceptibilities of Gram-negative bacilli responsible for ventilator-associated pneumonia. Crit Care Med. 2003;31:1908-1914.

S57. Guérin C, Girard R, Chemorin C, De Varax R, Fournier G: Facial mask noninvasive mechanical ventilation reduces the incidence of nosocomial pneumonia. Intens care Med. 1997;23(10):1024-32.

S58. Gursel G, Aydogdu M, Nadir Ozis T, Tasyurek S. Comparison of the value of initial and serial endotracheal aspirate surveillance cultures in predicting the causative pathogen of ventilator-associated pneumonia. Scand J Infect Dis. 2010, 42:341-346.

S59. Heyland DK, Cook DJ, Schoenfeld PS, Frietag A, Varon J, Wood G: The effect of acidified enteral feeds on gastric colonization in critically ill patients: results of a multicenter randomized trial. Canadian Critical Care Trials Group. Crit Care Med. 1999;27:2399-2406

S60. Holzapfel L, Chevret S, Madinier G, Ohen F, Demingeon G, Coupry A, Chaudet M: Influence of long-term oro- or nasotracheal intubation on nosocomial maxillary sinusitis and pneumonia: results of a prospective, randomized, clinical trial. Crit Care Med. 1993;21:1132-1138

S61. Huang SS, Septimus E, Kleinman K, Moody J, Hickok J, Avery TR, Lankiewicz J, Gombosev A, Terpstra L, Hartford F, Hayden MK. Targeted versus universal decolonization to prevent ICU infection. *N Engl J Med*. 2013;368(24):2255-65.

S62. Hugonnet S, Uçkay I, Pittet D Staffing level: a determinant of late-onset ventilator-associated pneumonia. Crit Care. 2007;11(4):R80

S63. Hyllienmark P, Gardlund B, Persson JO, Ekdahl K. Nosocomial pneumonia in the ICU: a prospective cohort study. Scand J Infect Dis. 2007;39:676-82.

S64. Hyllienmark P, Brattström O, Larsson E, Martling CR, Petersson J, Oldner A: High incidence of post-injury pneumonia in intensive care-treated trauma patients. Acta Anaesthesiologica Scandinavica. 2013;57(7):848-54.

S65. Ibáñez J, Peñafiel A, Marsé P, Jordá R, Raurich JM, Mata F: Incidence of gastroesophageal reflux and aspiration in mechanically ventilated patients using small-bore nasogastric tubes. J Parenteral and Enteral Nutrition. 2000;24(2):103-6.

S66. Ibrahim EH, Ward S, Sherman G, Kollef MH: A comparative analysis of patients with early-onset vs late-onset nosocomial pneumonia in the ICU setting. Chest. 2000;117:1434-1442

S67. Ibrahim EH, Sherman G, Ward S, Fraser VJ, Kollef MH. The influence of inadequate antimicrobial treatment of bloodstream infections on patient outcomes in the ICU setting. Chest. 2000;118(1):146-55.

S68. Ibrahim EH, Tracy L, Hill C, et al. The occurrence of ventilator-associated pneumonia in a community hospital: risk factors and clinical outcomes. *Chest* 2001;120:555-61.

S69. Jacobs S, Chang RW, Lee B, Bartlett FW: Continuous enteral feeding: a major cause of pneumonia among ventilated intensive care unit patients. *JPEN J Parenter Enteral Nutr* 1990;14:353-6.

S70. Jaillette E, Nseir S: Relationship between inhaled β2-agonists and ventilator-associated pneumonia: A cohort study. Critical Care Med. 2011;39(4):725-30.

S71. Jensen JUS, Hein L, Lundgren B, Bestle MH, Mohr T, Andersen MH, Procalcitonin And Survival Study Group (2015) Invasive *Candida* Infections and the Harm From Antibacterial Drugs in Critically Ill Patients: Data From a Randomized, Controlled Trial to Determine the Role of Ciprofloxacin, Piperacillin-Tazobactam, Meropenem, and Cefuroxime. *Crit Care Medicine* 2015;43(3):594-602.

S72. Jiménez P, Torres A, Rodríguez-Roisin R, de la Bellacasa JP, Aznar R, Gatell JM, Agustí-Vidal A: Incidence and etiology of pneumonia acquired during mechanical ventilation. Crit Care Med. 1989;17:882-5.

S73. Kautzky S, Staudinger T, Presterl E. Invasive Candida infections in patients of a medical intensive care unit. Wiener klinische Wochenschrift. 2015;127(3-4):132-42.

S74. Ko HK, Yu WK, Lien TC, Wang JH, Slutsky AS, Zhang H, Kou YR. Intensive care unit-acquired bacteremia in mechanically ventilated patients: clinical features and outcomes. *PloS one*. 2013;8(12):e83298.

S75. Kollef MH: Ventilator-associated pneumonia. A multivariate analysis. JAMA. 1993;270:1965-70.

S76. Kollef MH, Silver P, Murphy DM, Trovillion E: The effect of late-onset ventilator-associated pneumonia in determining patient mortality. Chest. 1995;108: 1655-62.

S77. Kollef MH, Shapiro SD, Fraser VJ, Silver P, Murphy DM, Trovillion E, Hearns ML, Richards RD, Cracchilo L, Hossin L: Mechanical ventilation with or without 7-day circuit changes. A randomized controlled trial. Ann Intern Med.1995;123:168–174

S78. Kollef MH, Von Harz B, Prentice D, Shapiro SD, Silver P, John RS, Trovillion E: Patient transport from intensive care increases the risk of developing ventilator-associated pneumonia. Chest. 1997;112(3):765-773.

S79. Kollef MH, Vlasnik JO, Sharpless L, Pasque C, Murphy D, Fraser V. Scheduled change of antibiotic classes: a strategy to decrease the incidence of ventilator-associated pneumonia. Am J Resp Crit Care Med. 1997;156(4):1040-8.

S80. Kollef MH, Chastre J, Fagon JY, François B, Niederman MS, Rello J, Torres A, Vincent JL, Wunderink RG, Go KW, Rehm C. Global prospective epidemiologic and surveillance study of ventilator-associated pneumonia due to Pseudomonas aeruginosa. Crit care med. 2014;42(10):2178-87.

S81. Koss WG, Khalili TM, Lemus JF, Chelly MM, Margulies DR, Shabot MM: Nosocomial pneumonia is not prevented by protective contact isolation in the surgical intensive care unit. Am Surg. 2001;67:1140-4.

S82. Kunac A, Sifri ZC, Mohr AM, Horng H, Lavery RF, Livingston DH: Bacteremia and Ventilator-Associated Pneumonia: A Marker for Contemporaneous Extra-Pulmonic Infection. Surg Infect. 2014;15:77-83.

S83. Laggner AN, Lenz K, Base W, Druml W, Schneeweiss B, Grimm G: Prevention of upper gastrointestinal bleeding in long-term ventilated patients. Sucralfate versus ranitidine. Am J Med. 1989;86:81-4.

S84. Lambert ML, Suetens C, Savey A, Palomar M, Hiesmayr M, Morales I, Agodi A, Frank U, Mertens K, Schumacher M, Wolkewitz M. Clinical outcomes of health-care-associated infections and antimicrobial resistance in patients admitted to European intensive-care units: a cohort study. Lancet Infect Dis. 2011;11(1):30-8.

S85. Laupland KB, Zygun DA, Davies HD, Church DL, Louie TJ, Doig CJ Population-based assessment of intensive care unit-acquired bloodstream infections in adults: incidence, risk factors, and associated mortality rate. *Crit Care Med* 2002;30:2462-2467.

S86. Laupland KB, Kirkpatrick AW, Church DL, Ross T, Gregson DB Intensive-care-unit-acquired bloodstream infections in a regional critically ill population. *J Hosp Infect* 2004;58(2): 137-145.

S87. León C, Ruiz-Santana S, Saavedra P, Almirante B, Nolla-Salas J, Álvarez-Lerma F, Garnacho-Montero J, León MÁ. A bedside scoring system ("Candida score") for early antifungal treatment in nonneutropenic critically ill patients with Candida colonization. Crit Care Med. 2006 Mar 1;34(3):730-7.

S88. León C, Ruiz-Santana S, Saavedra P, Galván B, Blanco A, Castro C, Balasini C, Utande-Vázquez A, de Molina FJ, Blasco-Navalproto MA, López MJ. Usefulness of the "Candida score" for discriminating between Candida colonization and invasive candidiasis in non-neutropenic critically ill patients: a prospective multicenter study. Crit Care Med. 2009;37(5):1624-33.

S89. León C, Ruiz-Santana S, Saavedra P, Castro C, Loza A, Zakariya I, Úbeda A, Parra M, Macías D, Tomás JI, Rezusta A. Contribution of Candida biomarkers and DNA detection for the diagnosis of invasive candidiasis in ICU patients with severe abdominal conditions. Crit Care. 2016;20(1):149.

S90. Lepelletier D, Roquilly A, Mahe PJ, Loutrel O, Champin P, Corvec S, Naux E, Pinaud M, Lejus C, Asehnoune K. Retrospective analysis of the risk factors and pathogens associated with early-onset ventilator-associated pneumonia in surgical-ICU head-trauma patients. J Neurosurg Anesthesiol. 2010;22(1):32-7.

S91. Li Z, Jiang C, Dong D, Zhang L, Tian Y, Ni Q, Mao E, Peng Y. The correlation between Candida colonization of distinct body sites and invasive candidiasis in emergency intensive care units: statistical and molecular biological analysis. Mycopathologia. 2016;181(7-8):475-84.

S92. Luna CM, Blanzaco D, Niederman MS, et al. Resolution of ventilator-associated pneumonia: prospective evaluation of the clinical pulmonary infection score as an early clinical predictor of outcome. Crit Care Med. 2003;31:676-682.

S93. Luyt CE, Guérin V, Combes A, Trouillet JL, Ayed SB, Bernard M, Gibert C, Chastre J: Procalcitonin kinetics as a prognostic marker of ventilator-associated pneumonia. Am J Respir Crit Care Med. 2005;171:48-53.

S94. Magnason S, Kristinsson KG, Stefansson T, Erlendsdottir H, Jonsdottir K, Kristjansson M, Gudmundsson S: Risk factors and outcome in ICU-acquired infections. Acta Anaesthesiol Scand. 2008;52:1238-1245

S95. Mahul P, Auboyer C, Jospe R, Ros A, Guerin C, el Khouri Z, Galliez M, Dumont A, Gaudin O: Prevention of nosocomial pneumonia in intubated patients respective role of mechanical subglottic secretions drainage and stress ulcer prophylaxis. Intensive Care Med. 1992;18:20-25

S96. Makris D, Manoulakas E, Komnos A, Papakrivou E, Tzovaras N, Hovas A, Zintzaras E, Zakynthinos E. Effect of pravastatin on the frequency of ventilator-associated pneumonia and on intensive care unit mortality: open-label, randomized study. Crit care med. 2011;39(11):2440-6.

S97. Markowicz P, Wolff M, Djedaini K, Cohen Y, Chastre J, Delclaux C: Multicenter prospective study of ventilator-associated pneumonia during acute respiratory distress syndrome. Incidence, prognosis, and risk factors. ARDS Study Group. Am J Respir Crit Care Med. 2000;161:1942-8.

S98. Memish ZA, Cunningham G, Oni GA, et al. The incidence and risk factors of ventilator-associated pneumonia in a Riyadh hospital. Infect Control Hosp Epidemiol. 2000;21:271-3.

S99. Michel F, Franceschini B, Berger P, Arnal JM, Gainnier M, Sainty JM, Papazian L. Early antibiotic treatment for BAL-confirmed ventilator-associated pneumonia: a role for routine endotracheal aspirate cultures. Chest. 2005;127(2):589-97.

S100. Mitsogianni M, Vasileiadis I, Parisi M, Tzanis G, Kampisiouli E, Psaroudaki Z, Perivolioti E, Fountoulis K, Routsi C, Nanas S, Tsiodras S. A Multifaceted Intervention Program to Prevent Bloodstream Infection in an IntensiveCare Unit. *Health Science J*. 2016;10(2):1.

S101. Moine P, Timsit JF, De Lassence A, Troché G, Fosse JP, Alberti C, Cohen Y: Mortality associated with late-onset pneumonia in the intensive care unit: results of a multi-center cohort study. Intensive Care Med. 2002;28:154-163

S102. Montecalvo MA, Steger KA, Farber HW: Nutritional outcome and pneumonia in critical care patients randomized to gastric versus jejunal tube feedings. The Critical Care Research Team. *Crit Care Med* 1992, 20:1377-1387.

S103. Myny D, Depuydt P, Colardyn F, Blot S: Ventilator-associated pneumonia in a tertiary care ICU analysis of risk factors for acquisition and mortality. Acta Clin Belg. 2005;60:114-121.

S104. Nguile-Makao M, Zahar JR, Français A, Tabah A, Garrouste-Orgeas M, Allaouchiche B, Goldgran-Toledano D, Azoulay E, Adrie C, Jamali S, Clec'h C. Attributable mortality of ventilator-associated pneumonia: respective impact of main characteristics at ICU admission and VAP onset using conditional logistic regression and multi-state models. Intens care med. 2010;36(5):781-9.

S105. Nielsen SL, Røder B, Magnussen P, Engquist A, Frimodt-møller N. Nosocomial pneumonia in an intensive care unit in a Danish university hospital: incidence, mortality and etiology. Scand J Infect Dis. 1992;24:65-70.

S106. Nseir S, Di Pompeo C, Soubrier S, Cavestri B, Jozefowicz E, Saulnier F, Durocher A: Impact of ventilator-associated pneumonia on outcome in patients with COPD. Chest. 2005;128(3):1650-1656.

S107. Nseir S, Jozefowicz E, Cavestri B, Sendid B, Di Pompeo C, Dewavrin F, Durocher A. Impact of antifungal treatment on Candida–Pseudomonas interaction: a preliminary retrospective case–control study. *Intensive Care Med* 2007;33(1):137-142.

S108. Orsi GB, Giuliano S, Franchi C, Ciorba V, Protano C, Giordano A, Rocco M, Venditti M. Changed epidemiology of ICU acquired bloodstream infections over 12 years in an Italian teaching hospital. *Minerva Anestesiol.* 2015;81(9):980-8.

S109. Osmon S, Warren D, Seiler SM, Shannon W, Fraser VJ, Kollef MH: The influence of infection on hospital mortality for patients requiring >48 h of intensive care. *Chest* 2003, 124:1021-1029.

S110. Saied WI, Mourvillier B, Cohen Y, Ruckly S, Reignier J, Marcotte G, Siami S, Bouadma L, et al on behalf of the OUTCOMEREA study group. A comparison of the mortality risk associated with ventilator-acquired bacterial pneumonia and nonventilator ICU-acquired bacterial pneumonia. Crit care med. 2019;47:345-52.

S111. Papazian L, Bregeon F, Thirion X, Gregoire R, Saux P, Denis JP, Perin G, Charrel J, Dumon JF, Affray JP, Gouin F: Effect of ventilator-associated pneumonia on mortality and morbidity. Am J Respir Crit Care Med. 1996;154:91-7.

S112. Petri MG, König J, Moecke HP, Gramm HJ, Barkow H, Kujath P, Dennhart R, Lode H. Epidemiology of invasive mycosis in ICU patients: a prospective multicenter study in 435 non-neutropenic patients. *Intensive Care Med* 1997;23(3):317-325.

S113. Potgieter PD, Linton DM, Oliver S, Forder AA: Nosocomial infections in a respiratory intensive care unit. Crit Care Med. 1987;15:495-498

S114. Prowle JR, Echeverri JE, Ligabo EV, Sherry N, Taori GC, Crozier TM, Bellomo R. Acquired bloodstream infection in the intensive care unit: incidence and attributable mortality. Crit Care 2011;15(2):R100.

S115. Ramirez P, Lopez-Ferraz C, Gordon M, Gimeno A, Villarreal E, Ruiz J, Menendez R, Torres A. From starting mechanical ventilation to ventilator-associated pneumonia, choosing the right moment to start antibiotic treatment. *Crit Care* 2016;20(1):169.

S116. Rello J, Quintana E, Ausina V, Castella J, Luquin M, Net A, Prats G: Incidence, etiology, and outcome of nosocomial pneumonia in mechanically ventilated patients. Chest. 1991;100:439-444

S117. Rello J, Ausina V, Castella J, et al Nosocomial respiratory tract infections in multiple trauma patients. Influence of level of consciousness with implications for therapy. Chest 1992;102:525-529

S118. Rello J, Ricart M, Mirelis B, Quintana E, Gurgui M, Net A, Prats, G: Nosocomial bacteremia in a medical-surgical intensive care unit: epidemiologic characteristics and factors influencing mortality in 111 episodes. *Intensive Care Med* 1994;20:94-98.

S119. Rello J, Ausina V, Ricart M, Puzo C, Quintana E, Net A, Prats G. Risk factors for infection by *Pseudomonas aeruginosa* in patients with ventilator-associated pneumonia. *Intens Care Med*. 1994;20(3):193-8.

S120. Rello J, Sonora R, Jubert P, et al. Pneumonia in intubated patients: role of respiratory airway Care *Am J Respir Crit Care Med* 1996;154:111-5.

S121. Rello J, Ollendorf DA, Oster G, et al. Epidemiology and outcomes of ventilator-associated pneumonia in a large US database. Chest 2002;122:2115-2121

S122. Rello J, Lorente C, Diaz E, et al. Incidence, etiology, and outcome of nosocomial pneumonia in ICU patients requiring percutaneous tracheotomy for mechanical ventilation. *Chest*. 2003;124:2239-2243.

S123. Resende MM, Monteiro SG, Callegari B, Figueiredo PM, Monteiro CR, Monteiro-Neto V. Epidemiology and outcomes of ventilator-associated pneumonia in northern Brazil: an analytical descriptive prospective cohort study. BMC infect Dis 2013;13(1): 119

S124. Reusser P, Zimmerli W, Scheidegger D, Marbet GA, Buser M, Gyr K: Role of gastric colonization in nosocomial infections and endotoxemia: a prospective study in neurosurgical patients on mechanical ventilation. J Infect Dis. 1989;160:414-421

S125. Rincón-Ferrari MD, Flores-Cordero JM, Leal-Noval SR, Murillo-Cabezas F, Cayuelas A, Muñoz-Sánchez MA, Sánchez-Olmedo JI: Impact of ventilator-associated pneumonia in patients with severe head injury. J Trauma Acute Care Surg. 2004;57(6):1234-40.

S126. Rodrigues PM, Neto C, Santos LR, Knibel MF. Ventilator-associated pneumonia: epidemiology and impact on the clinical evolution of ICU patients. Jornal brasileiro de pneumologia. 2009 Nov;35(11):1084-91.

S127. Rodriguez JL, Gibbons KJ, Bitzer LG, Dechert RE, Steinberg SM, Flint LM: Pneumonia: incidence, risk factors, and outcome in injured patients. J Trauma. 1991;31: 907-12.

S128. Ruiz-Santana S, Garcia Jimenez A, Esteban A, et al. ICU pneumonias: a multi-institutional study. *Crit Care Med*. 1987;15:930-932.

S129. Salata RA, Lederman MM, Shlaes DM, Jacobs MR, Eckstein E, Tweardy D, Toossi Z, Chmielewski R, Marino J, King CH: Diagnosis of nosocomial pneumonia in intubated, intensive care unit patients. Am Rev Respir Dis. 1987;135:426-432

S130. Shahin J, Bielinski M, Guichon C, Flemming C, Kristof AS Suspected ventilator-associated respiratory infection in severely ill patients: a prospective observational study. Crit Care 2013;17(5): R251

S131. Sofianou DC, Constandinidis TC, Yannacou M, Anastasiou H, Sofianos E: Analysis of risk factors for ventilator-associated pneumonia in a multidisciplinary intensive care unit. *Eur J Clin Microbiol Infect Dis* 2000, 19:460-463.

S132. Stéphan F, Mabrouk N, Decailliot F, Delclaux C, Legrand P: Ventilator-associated pneumonia leading to acute lung injury after trauma: importance of *Haemophilus influenzae*. Anesthesiology. 2006;104: 235-41.

S133. Tan X, Zhu S, Yan D, Chen W, Chen R, Zou J, Yan J, Zhang X, Farmakiotis D, Mylonakis E. Candida spp. airway colonization: A potential risk factor for Acinetobacter baumannii ventilator-associated pneumonia. Med Mycol. 2016:myw009.

S134. Tejada Artigas AT, Dronda SB, Vallés EC, Marco JM, Usón MC, Figueras P, Suarez FJ, Hernandez A: Risk factors for nosocomial pneumonia in critically ill trauma patients. Crit Care Med. 2001;29:304-9.

S135. Thompson DS. Estimates of the rate of acquisition of bacteraemia and associated excess mortality in a general intensive care unit: a 10 year study. *J Hosp Infect*. 2008;69(1):56-61.

S136. Timsit JF, Chevret S, Valcke J, Misset B, Renaud B, Goldstein FW, Vaury P, Carlet J: Mortality of nosocomial pneumonia in ventilated patients: influence of diagnostic tools. Am J Respir Crit Care Med. 1996;154:116-23.

S137. Torres A, Aznar R, Gatell JM, Jiménez P, González J, Ferrer A, Celis R, Rodriguez-Roisin R: Incidence, risk, and prognosis factors of nosocomial pneumonia in mechanically ventilated patients. Am Rev Respir Dis. 1990;142:523-8.

S138. Trouillet JL, Chastre J, Vuagnat A, Joly-Guillou ML, Combaux D, Dombret MC, Gibert C: Ventilator-associated pneumonia caused by potentially drug-resistant bacteria. Am J Respir Crit Care Med. 1998;157(2):531-9.

S139. Urli T, Perone G, Acquarolo A, Zappa S, Antonini B, Ciani A: Surveillance of infections acquired in intensive care: usefulness in clinical practice. *J Hosp Infect* 2002, 52:130-5.

S140. Valles J, Pobo A, Garcia-Esquirol O, Mariscal D, Real J, Fernández R. Excess ICU mortality attributable to ventilator-associated pneumonia: the role of early vs late onset. *Intensive care medicine,* 2007;33(8):1363-1368.

S141. Vanhems P, Bénet T, Voirin N, Januel JM, Lepape A, Allaouchiche B, Argaud L, Chassard D, Guérin C. Early-onset ventilator-associated pneumonia incidence in intensive care units: a surveillance-based study. BMC Infect Dis. 2011;11(1):236.

S142. Verhamme KM, De Coster W, De Roo L, De Beenhouwer H, Nollet G, Verbeke J, Demeyer I, Jordens P: Pathogens in early-onset and late-onset intensive care unit–acquired pneumonia. Infection Control Hospital Epidemiol. 2007;28(4):389-397.

S143. Violan JS, Sanchez-Ramirez C, Mujica AP, Cendrero JC, Fernandez JA, de Castro FR: Impact of nosocomial pneumonia on the outcome of mechanically-ventilated patients. Crit Care (Lond). 1998;2:19-23.

S144. Warren DK, Zack JE, Elward AM, Cox MJ, Fraser VJ. Nosocomial primary bloodstream infections in intensive care unit patients in a nonteaching community medical center: a 21-month prospective study. *Clin Infect Dis.* 2001;33(8):1329-35.

S145. Woske HJ, Röding T, Schulz I, Lode H: Ventilator-associated pneumonia in a surgical intensive care unit Epidemiology, etiology and comparison of three bronchoscopic methods for microbiological specimen sampling. Crit Care. 2001;5:167–173.

S146. Xie DS, Xiong W, Lai RP, Liu L, Gan XM, Wang XH, Nie SF. Ventilator-associated pneumonia in intensive care units in Hubei Province, China: a multicentre prospective cohort survey. J Hosp Infect 2011;78(4): 284-288

S147. Zahar JR, Nguile-Makao M, Français A, Schwebel C, Garrouste-Orgeas M, Goldgran-Toledano D, Azoulay E, Thuong M, Jamali S, Cohen Y, De Lassence A. Predicting the risk of documented ventilator-associated pneumonia for benchmarking: construction and validation of a score. Crit care med. 2009;37(9):2545-51.

S148. Acosta-Escribano J, Fernández-Vivas M, Carmona TG, Caturla-Such J, Garcia-Martinez M, Menendez-Mainer A, Sanchez-Payá J (2010) Gastric versus transpyloric feeding in severe traumatic brain injury: a prospective, randomized trial. Intensive Care Med 36:1532-1539

S149. Bonten MJ, Gaillard CA, Van der Geest S, Van Tiel FH, Beysens AJ, Smeets HG, Stobberingh EE: The role of intragastric acidity and stress ulcer prophylaxis on colonization and infection in mechanically ventilated ICU patients. A stratified, randomized, double-blind study of sucralfate versus antacids. Am J Respir Crit Care Med. 1995;152:1825-1834.

S150. Cook D, Guyatt G, Marshall J, et al A comparison of sucralfate and ranitidine for the prevention of upper gastrointestinal bleeding in patients requiring mechanical ventilation. Canadian Critical Care Trials Group. N Engl J Med 1998;338:791-797

S151. Daumal F, Colpart E, Manoury B, Mariani M, Daumal M. Changing heat and moisture exchangers every 48 hours does not increase the incidence of nosocomial pneumonia. Infection Control & Hospital Epidemiology. 1999;20(5):347-9.

S152. Djedaini K, Billiard M, Mier L, Le Bourdelles G, Brun P, Markowicz P, Estagnasie P, Coste F, Boussougant Y, Dreyfuss D: Changing heat and moisture exchangers every 48 hours rather than 24 hours does not affect their efficacy and the incidence of nosocomial pneumonia. Am J Respir Crit Care Med. 1995;152(5):1562-9.

S153. Drakulovic MB, Torres A, Bauer TT, Nicolas JM, Nogué S, Ferrer M: Supine body position as a risk factor for nosocomial pneumonia in mechanically ventilated patients: a randomised trial. Lancet. 1999;354(9193):1851-1858

S154. Dreyfuss D, Djedaini K, Weber P, Brun P, Lanore JJ, Rahmani J, Coste F: Prospective study of nosocomial pneumonia and of patient and circuit colonization during mechanical ventilation with circuit changes every 48 hours versus no change. Am Rev Respir Dis. 1991;143(4 Pt 1), 738-743.

S155. Dreyfuss D, Djedaïni K, Gros I, Mier L, Le Bourdellés G, Cohen Y, Estagnasié P, Coste F, Boussougant Y: Mechanical ventilation with heated humidifiers or heat and moisture exchangers: effects on patient colonization and incidence of nosocomial pneumonia. Am J Respir Crit Care Med. 1995;151:986-92.

S156. Driks MR, Craven DE, Celli BR, et al (1987) Nosocomial pneumonia in intubated patients given sucralfate as compared with antacids or histamine type 2 blockers. The role of gastric colonization. N Engl J Med 317:1376-1382

S157. Forestier C, Guelon D, Cluytens V, Guillart T, Sirot J, De champs C: Oral probiotic and prevention of *Pseudomonas aeruginosa* infections: a randomized, double-blind, placebo controlled pilot study in intensive care unit patients. *Crit Care* 2008;12:R69.

S158. Heyland DK, Cook DJ, Schoenfeld PS, Frietag A, Varon J, Wood G The effect of acidified enteral feeds on gastric colonization in critically ill patients: results of a multicenter randomized trial. Canadian Critical Care Trials Group. Crit Care Med 1999;27:2399-2406

S159. Holzapfel L, Chastang C, Demingeon G, Bohe J, Piralla B, Coupry A: A randomized study assessing the systematic search for maxillary sinusitis in nasotracheally mechanically ventilated patients. Influence of nosocomial maxillary sinusitis on the occurrence of ventilator-associated pneumonia. Am J Respir Crit Care Med. 1999;159:695-701

S160. Kappstein I, Schulgen G, Friedrich T, Hellinger P, Benzing A, Geiger K, Daschner FD. Incidence of pneumonia in mechanically ventilated patients treated with sucralfate or cimetidine as prophylaxis for stress bleeding: bacterial colonization of the stomach. Am J Med. 1991;91(2):S125-31

S161. Kirschenbaum L, Azzi E, Sfeir T, et al. Effect of continuous lateral rotational therapy on the prevalence of ventilator-associated pneumonia in patients requiring long-term ventilatory care *Crit Care Med* 2002;30:1983-6.

S162. Kirton OC, DeHaven B, Morgan J, et al. A prospective, randomized comparison of an in-line heat moisture exchange filter and heated wire humidifiers: rates of ventilator-associated early-onset (community-acquired) or late-onset (hospital-acquired) pneumonia and incidence of endotracheal tube occlusion. *Chest* 1997;112:1055-9.

S163. Knight DJ, Gardiner D, Banks A, Snape SE, Weston VC, Bengmark S, Girling KJ: Effect of synbiotic therapy on the incidence of ventilator associated pneumonia in critically ill patients: a randomised, double-blind, placebo-controlled trial. Intensive Care Med. 2009;35:854-861.

S164. Kollef MH, Afessa B, Anzueto A, Veremakis C, Kerr KM, Margolis BD, Schinner R: Silver-coated endotracheal tubes and incidence of ventilator-associated pneumonia: the NASCENT randomized trial. JAMA. 2008;300(7):805-813

S165. Kortbeek JB, Haigh PI, Doig C. Duodenal versus gastric feeding in ventilated blunt trauma patients: a randomized controlled trial. *J Trauma* 1999;46:992-6.

S166. Lacherade JC, Auburtin M, Cerf C, Van de Louw A, Soufir L, Rebufat Y, Rezaiguia S, Ricard JD, Lellouche F, Brun-Buisson C, Brochard L: Impact of humidification systems on ventilator-associated pneumonia: a randomized multicenter trial. Am J Respir Crit Care Med. 2005;172:1276-1282

S167. Lacherade JC, De Jonghe B, Guezennec P, Debbat K, Hayon J, Monsel A, Bastuji-Garin S: Intermittent subglottic secretion drainage and ventilator-associated pneumonia A multicenter trial. Am J Respir Crit Care Med. 2010;182:910-917.

S168. Launey Y, Nesseler N, Le Cousin A, Feuillet F, Garlantezec R, Mallédant Y, Seguin P: Effect of a fever control protocol-based strategy on ventilator-associated pneumonia in severely brain-injured patients. Crit Care. 2014;18(6):1.

S169. Lorente L, Lecuona M, Málaga J, Revert C, Mora ML, Sierra A: Bacterial filters in respiratory circuits: an unnecessary cost? Crit Care Med 2003;31:2126-2130

S170. Lorente L, Lecuona M, Galván R, Ramos MJ, Mora ML, Sierra A: Periodically changing ventilator circuits is not necessary to prevent ventilator-associated pneumonia when a heat and moisture exchanger is used. Infect Control Hosp Epidemiol. 2004;25:1077-1082

S171. Lorente L, Lecuona M, Martín MM, García C, Mora ML, Sierra A: Ventilator-associated pneumonia using a closed versus an open tracheal suction system. Crit Care Med. 2005;33:115-119

S172. Lorente L, Lecuona M, Jiménez A, Mora ML, Sierra A: Tracheal suction by closed system without daily change versus open system. Intensive Care Med. 2006;32:538-44.

S173. Lorente L, Lecuona M, Jimenez A, Mora ML, Sierra A: Ventilator-associated pneumonia using a heated humidifier or a heat and moisture exchanger: a randomized controlled trial [ISRCTN88724583]. Crit Care 2006;10:R116

S174. Lorente L, Lecuona M, Jimenez A, Mora ML, Sierra: Influence of an endotracheal tube with polyurethane cuff and subglottic secretion drainage on pneumonia. Am J Respir Crit Care Med. 2007;176:1079-1083

S175. Lorente L, Lecuona M, Jiménez A, Lorenzo L, Roca I, Cabrera J, Llanos C, Mora ML: Continuous endotracheal tube cuff pressure control system protects against ventilator-associated pneumonia. Crit Care. 2014;18(2):1.

S176. Manzano F, Fernandez-Mondejar E, Colmenero M, Poyatos ME, Rivera R, Machado J, Catalan I, Artigas A: Positive-end expiratory pressure reduces incidence of ventilator-associated pneumonia in nonhypoxemic patients. Crit Care Med: 2008;36(8):2225-31.

S177. Martin C, Perrin G, Gevaudan MJ, Saux P, Gouin F. Heat and moisture exchangers and vaporizing humidifiers in the intensive care unit. Chest. 1990;97(1):144-9.

S178. Morrow LE, Kollef MH, Casale TB: Probiotic prophylaxis of ventilator-associated pneumonia: a blinded, randomized, controlled trial. Am J Respir Crit Care Med. 2010;182:1058-1064

S179. Nseir S, Zerimech F, Fournier C, Lubret R, Ramon P, Durocher A, Balduyck M: Continuous control of tracheal cuff pressure and microaspiration of gastric contents in critically ill patients. Am J Respir Crit Care Med. 2011;184(9):1041-7.

S180. Pickworth KK, Falcone RE, Hoogeboom JE, et al Occurrence of nosocomial pneumonia in mechanically ventilated trauma patients: a comparison of sucralfate and ranitidine. Crit Care Med 1993;21:1856-1862

S181. Pneumatikos I, Konstantonis D, Tsagaris I, Theodorou V, Vretzakis G, Danielides V, Bouros D: Prevention of nosocomial maxillary sinusitis in the ICU: the effects of topically applied alpha-adrenergic agonists and corticosteroids. Intensive Care Med. 2006;32:532-537

S182. Prod'hom G, Leuenberger P, Koerfer J, Blum A, Chiolero R, Schaller MD, Perret C, Spinnler O, Blondel J, Siegrist H, Saghafi L: Nosocomial pneumonia in mechanically ventilated patients receiving antacid, ranitidine, or sucralfate as prophylaxis for stress ulcer. A randomized controlled trial. Ann Intern Med. 1994;120:653-62.

S183. Reignier J, Mercier E, Le Gouge A, Boulain T, Desachy A, Bellec F, Lascarrou JB: Effect of Not Monitoring Residual Gastric Volume on Risk of Ventilator-Associated Pneumonia in Adults Receiving Mechanical Ventilation and Early Enteral Feeding. A Randomized Controlled Trial. *JAMA* 2013, 309;249-256.

S184. Rumbak MJ, Truncale T, Newton MN, Adams B, Hazard P. A Prospective, Randomized Study Comparing Early Versus Delayed Percutaneous Tracheostomy In Critically Ill Medical Patients Requiring Prolonged Mechanical Ventilation. Chest. 2000;118(4):97S-8S.

S185. Ryan P, Dawson J, Teres D, Celoria G, Navab F: Nosocomial pneumonia during stress ulcer prophylaxis with cimetidine and sucralfate. Arch Surg. 1993;128(12):1353-7.

S186. Smulders K, van der Hoeven H, Weers-Pothoff I, Vandenbroucke-Grauls C A randomized clinical trial of intermittent subglottic secretion drainage in patients receiving mechanical ventilation. Chest 2002;121:858-862

S187. Staudinger T, Bojic A, Holzinger U, Meyer B, Rohwer M, Mallner F, Locker GJ Continuous lateral rotation therapy to prevent ventilator-associated pneumonia Crit Care Med 2010;38(2):486-490

S188. Thomachot L, Viviand X, Arnaud S, Boisson C, Martin CD: Comparing two heat and moisture exchangers, one hydrophobic and one hygroscopic, on humidifying efficacy and the rate of nosocomial pneumonia. Chest. 1998;114:1383-1389

S189. Thomachot L, Leone M, Razzouk K, Antonini F, Vialet R, Martin C: Do the components of heat and moisture exchanger filters affect humidifying efficacy and the incidence of nosocomial pneumonia? Crit Care Med. 1999;27:923–928

S190. Thomachot L, Leone M, Razzouk K, Antonini F, Vialet R, Martin C: Randomized Clinical Trial of Extended Use of a Hydrophobic Condenser Humidifier: 1 vs 7 Days. Crit Care Med. 2002;30:232-7

S191. Valencia M, Ferrer M, Farre R, Navajas D, Badia JR, Nicolas JM, Torres A: Automatic control of tracheal tube cuff pressure in ventilated patients in semirecumbent position: a randomized trial. Crit Care Med. 2007;35: 1543-9.

S192. Valles J, Artigas A, Rello J, et al. Continuous aspiration of subglottic secretions in preventing ventilator-associated pneumonia. *Ann Intern Med* 1995;122:179-86.

S193. Walaszek M, Gniadek A, Kolpa M, Wolak Z, Kosiarska A. The effect of subglottic secretion drainage on the incidence of ventilator associated pneumonia. *Biomed Pap Med Fac Univ Palacky Olomouc Czech Repub.* 2017;161(4):374-80.

S194. Zeng J, Wang CT, Zhang FS, Qi F, Wang SF, Ma S, Wu TJ, Tian H, Tian ZT, Zhang SL, Qu Y. Effect of probiotics on the incidence of ventilator-associated pneumonia in critically ill patients: a randomized controlled multicenter trial. Intens care med. 2016;42(6):1018-28.

S195. Bellissimo-Rodrigues WT, Menegueti MG, Gaspar GG, Nicolini EA, Auxiliadora-Martins M, Basile-Filho A, Bellissimo-Rodrigues F. Effectiveness of a Dental Care Intervention in the Prevention of Lower Respiratory Tract Nosocomial Infections among Intensive Care Patients: A Randomized Clinical Trial. Infect Control Hosp Epidemiol 2014, 35:1342-1348

S196. Bleasdale SC, Trick WE, Gonzalez IM, Lyles RD, Hayden MK, Weinstein RA. Effectiveness of chlorhexidine bathing to reduce catheter-associated bloodstream infections in medical intensive care unit patients. Arch intern med. 2007;167(19):2073-9.

S197. Ćabov T, Macan D, Husedžinović I, Škrlin-Šubić J, Bošnjak D, Šestan-Crnek S, Perić B, Kovač Z, Golubović V. The impact of oral health and 0.2% chlorhexidine oral gel on the prevalence of nosocomial infections in surgical intensive-care patients: a randomized placebo-controlled study. Einfluss von Mundgesundheit und von 0, 2% Chlorhexidin-Gel auf die Entwicklung von nosokomialen Infektionen bei Patienten auf einer chirurgischen Intensivstation. Wiener klinische Wochenschrift. 2010;122(13-14):397-404.

S198. Caruso P, Denari S, Ruiz SA, Demarzo SE, Deheinzelin D. Saline instillation before tracheal suctioning decreases the incidence of ventilator-associated pneumonia. Crit Care Med 2009, 37:32-38.

S199. Climo MW, Yokoe DS, Warren DK et al. Effect of daily chlorhexidine bathing on hospital-acquired infection. *N Engl J Med* 2013; 368: 533–542.

S200. Fourrier FE, Cau-Pottier H, Boutigny M, Roussel-Delvallez M, Jourdain, Chopin C: Effects of dental plaque antiseptic decontamination on bacterial colonization and nosocomial infections in critically ill patients. Intensive Care Med. 2000;26:1239-1247

S201. Fourrier F, Dubois D, Pronnier P, Herbecq P, Leroy O, Desmettre T, Roussel-Delvallez M: Effect of gingival and dental plaque antiseptic decontamination on nosocomial infections acquired in the intensive care unit a double-blind placebo-controlled multicenter study. Crit Care Med. 2005;33:1728-1735

S202. Koeman M, van der Ven AJ, Hak E, et al. Oral decontamination with chlorhexidine reduces the incidence of ventilator-associated pneumonia. Am J Respir Crit Care Med 2006;173:1348-1355

S203. Kollef M, Pittet D, Sanchez Garcia M, et al. A randomized double-blind trial of iseganan in prevention of ventilator-associated pneumonia. *Am J Respir Crit Care Med* 2006: 173:91-7.

S204. Lorente L, Lecuona M, Jiménez A, Palmero S, Pastor E, Lafuente N, Ramos MJ, Mora ML, Sierra A: Ventilator-associated pneumonia with or without toothbrushing a randomized controlled trial. Eur J Clin Microbiol Infect Dis. 2012;31:1-9

S205. Mori H, Hirasawa H, Oda S, Shiga H, Matsuda K, Nakamura M: Oral care reduces incidence of ventilator-associated pneumonia in ICU populations. Intensive Care Med, 2006, 32(2), 230-236.

S206. Noto MJ, Domenico HJ, Byrne DW, Talbot T, Rice TW, Bernard GR, Wheeler AP. Chlorhexidine bathing and health care–associated infections: a randomized clinical trial. *JAMA.* 2015;313(4):369-78.

S207. Seguin P, Tanguy M , Laviolle B, Tirel O, Malledant Y: Effect of oropharyngeal decontamination by povidone-iodine on ventilator-associated pneumonia in patients with head trauma. *Crit Care Med* 2006, 34:1514-1519.

S208. Seguin P, Laviolle B, Dahyot-Fizelier C, Dumont R, Veber B, Gergaud S, Asehnoune K, Mimoz O, Donnio PY, Bellissant E, Malledant Y. Effect of oropharyngeal povidone-iodine preventive oral care on ventilator-associated pneumonia in severely brain-injured or cerebral hemorrhage patients: a multicenter, randomized controlled trial. Crit care med. 2014;42(1):1-8.

S209. Swan JT, Ashton CM, Bui LN, Pham VP, Shirkey BA, Blackshear JE, Bersamin JB, Pomer RM, Johnson ML, Magtoto AD, Butler MO. Effect of chlorhexidine bathing every other day on prevention of hospital-acquired infections in the surgical ICU: a single-center, randomized controlled trial. Crit Care Med. 2016;44(10):1822-32.

S210. Wittekamp BH, Plantinga NL, Cooper BS, Lopez-Contreras J, Coll P, Mancebo J, Wise MP, Morgan MP, Depuydt P, Boelens J, Dugernier T. Decontamination strategies and bloodstream infections with antibiotic-resistant microorganisms in ventilated patients: a randomized clinical trial. *JAMA*. 2018.

S211. Bergmans DC, Bonten MJ, Gaillard CA, et al Prevention of ventilator-associated pneumonia by oral decontamination: a prospective, randomized, double-blind, placebo-controlled study. Am J Respir Crit Care Med 2001;164:382-388

S212. Bonten MJ, Gaillard CA, Johanson Jr WG, Van Tiel FH, Smeets HG, Van Der Geest S, Stobberingh EE. Colonization in patients receiving and not receiving topical antimicrobial prophylaxis. *Am J Respir Crit Care Med* 1994;150(5):1332-1340.

S213. Camus C, Salomon S, Bouchigny C, Gacouin A, Lavoué S, Donnio PY, Javaudin L, Chapplain JM, Uhel F, Le Tulzo Y, Bellissant E. Short-term decline in all-cause acquired infections with the routine use of a decontamination regimen combining topical polymyxin, tobramycin, and amphotericin B with mupirocin and chlorhexidine in the ICU: a single-center experience. *Crit Care Med.* 2014;42(5):1121-30.

S214. de Smet AMGA, Kluytmans JAJW, Cooper BS, Mascini EM, Benus RFJ, van der Werf TS, van der Hoeven JG, Pickkers P, Bogaers-Hofman D, van der Meer NJ, Bernards AT, Kuijper EJ, Joore JC, Leverstein-van Hall MA, Bindels AJ, Jansz AR, Wesselink RM, de Jongh BM, Dennesen PJ, van Asselt GJ, te Velde LF, Frenay IH, Kaasjager K, Bosch FH, van Iterson M, Thijsen SF, Kluge GH, Pauw W, de Vries JW, Kaan JA, Arends JP, Aarts LP, Sturm PD, Harinck HI, Voss A, Uijtendaal EV, Blok HE, Thieme Groen ES, Pouw ME, Kalkman CJ, Bonten MJ: Decontamination of the digestive tract and oropharynx in ICU patients. *N Engl J Med* 2009, 360:20–31.

S215. Frencken JF, Wittekamp BH, Plantinga NL, Spitoni C, van de Groep K, Cremer OL, Bonten MJ. Associations Between Enteral Colonization With Gram-Negative Bacteria and Intensive Care Unit–Acquired Infections and Colonization of the Respiratory Tract. *Clin Infect Dis*. 2017;66(4):497-503.

S216. Garbino J, Lew DP, Romand JA, Hugonnet S, Auckenthaler R, Pittet D: Prevention of severe Candida infections in nonneutropenic, high-risk, critically ill patients: a randomized, double-blind, placebo-controlled trial in patients treated by selective digestive decontamination. Intensive Care Med 2002;28:1708-1717

S217. Godard J, Guillaume C, Reverdy ME, Bachmann P, Bui-Xuan B, Nageotte A, Motin J: Intestinal decontamination in a polyvalent ICU. A double-blind study. *Intensive Care Med* 1990, 16:307-311.

S218. Hartenauer U, Thulig B, Diemer W, Lawin P, Fegeler W, Kehrel R, Ritzerfeld W Effect of selective flora suppression on colonization, infection, and mortality in critically ill patients: a one-year, prospective consecutive study. *Crit Care Med* 1991;19:463-473

S219. Hjortrup A, Rasmussen A, Hansen BA, Hoiby N, Heslet L, Moesgaard F, Kirkegaard P (1997) Early bacterial and fungal infections in liver transplantation after oral selective bowel decontamination. *Transplantation proceedings* 29:3106-3110

S220. Konrad F, Schwalbe B, Heeg K, et al. [Frequency of colonization and pneumonia and development of resistance in long-term ventilated intensive-care patients subjected to selective decontamination of the digestive tract]. *Anaesthesist* 1989;38:99-109.

S221. Landelle C, Boyer VN, Abbas M, Genevois E, Abidi N, Naimo S, Raulais R, Bouchoud L, Boroli F, Terrisse H, Bosson JL. Impact of a multifaceted prevention program on ventilator-associated pneumonia including selective oropharyngeal decontamination. *Intensive Care Med* 2018;44(11):1777-86.

S222. Ledingham I, Eastaway A, Mckay I, Alcock S, Mcdonald J, Ramsay G: Triple regimen of selective decontamination of the digestive tract, systemic cefotaxime, and microbiological surveillance for prevention of acquired infection in intensive care. *Lancet* 1988, 1:785-90.

S223. Leone M, Bourgoin A, Giuly E, et al. Influence on outcome of ventilator-associated pneumonia in multiple trauma patients with head trauma treated with selected digestive decontamination. *Crit Care Med* 2002; 30:1741-6.

S224. Nardi G, Valentinis U, Bartaletti R, Bello A, De AM, Muzzi R, Giordano F, Troncon MG. Effectiveness of topical selective decontamination, without systemic antibiotic prophylaxis, in prevention of pulmonary infection in intensive care. *Minerva anestesiologica.* 1990;56(1-2):19-26.

S225. Nardi G, Di Silvestre A, De Monte A, Massarutti D, Proietti A, Troncon MG, Zussino M: Reduction in gram-positive pneumonia and antibiotic consumption following the use of a SDD protocol including nasal and oral mupirocin. Eur J Emerg Med 2001;8:203-214

S226. Ong DS, Bonten MJ, Safdari K, Spitoni C, Frencken JF, Witteveen E, Horn J, Klein Klouwenberg PM, Cremer OL, MARS consortium, de Beer FM. Epidemiology, management, and risk-adjusted mortality of ICU-acquired enterococcal bacteremia. *Clin Infect Dis* 2015;61(9):1413-20.

S227. Oostdijk EAN, Kesecioglu J, Schultz MJ, et al. Notice of Retraction and Replacement: Oostdijk et al. Effects of Decontamination of the Oropharynx and Intestinal Tract on Antibiotic Resistance in ICUs: A Randomized Clinical Trial. *JAMA*. 2014;312(14):1429-1437. *JAMA* 2017

S228. Rouby JJ, Poete P, de Lassale EM, Nicolas MH, Bodin L, Jarlier V, Korinek AM, Viars P. Prevention of Gram negative noscomial bronchopneumonia by intratracheal colistin in critically ill patients. *Intensive Care Med*. 1994;20(3):187-92.

S229. Silvestri L, Bragadin CM, Milanese M, Gregori D, Consales C, Gullo A, Van Saene HK. Are most ICU infections really nosocomial? A prospective observational cohort study in mechanically ventilated patients. *J Hosp Infect.* 1999;42(2):125-33.

S230. Steffen R, Reinhartz O, Blumhardt G, Bechstein WO, Raakow R, Langrehr JM, Rossaint R, Slama K, Neuhaus P. Bacterial and fungal colonization and infections using oral selective bowel decontamination in orthotopic liver transplantations. *Transpl Inter.* 1994;7(2):101-8.

S231. Stoutenbeek CP, Van Saene HK, Miranda DR, Zandstra DF. The effect of selective decontamination of the digestive tract on colonisation and infection rate in multiple trauma patients. Intens Care Med. 1984;10(4):185-92.

S232. Stoutenbeek CP, van Saene HK, Miranda DR, Zandstra DF, Langrehr D; The effect of oropharyngeal decontamination using topical nonabsorbable antibiotics on the incidence of nosocomial respiratory tract infections in multiple trauma patients. J Trauma 1987;27:357-364

S233. Vallés J, Peredo R, Burgueño MJ, de Freitas AP, Millán S, Espasa M, Martín-Loeches I, Ferrer R, Suarez D, Artigas A. Efficacy of single-dose antibiotic against early-onset pneumonia in comatose patients who are ventilated. *Chest.* 2013;143(5):1219-25.

S234. Veelo DP, Bulut T, Dongelmans DA, et al. The incidence and microbial spectrum of ventilator-associated pneumonia after tracheotomy in a selective decontamination of the digestive tract-setting. *J Infect* 2008; 56:20-6.

S235. Winter R, Humphreys H, Pick A, MacGowan AP, Willatts SM, Speller DC: A controlled trial of selective decontamination of the digestive tract in intensive care and its effect on nosocomial infection. J Antimicrob Chemother. 1992;30:73-87

S236. Abele-Horn M, Dauber A, Bauernfeind A, Russwurm W, Seyfarth-Metzger I, Gleich P, Ruckdeschel G: Decrease in nosocomial pneumonia in ventilated patients by selective oropharyngeal decontamination (SOD). Intensive Care Med. 1997;23:187-95.

S237. Acquarolo A, Urli T, Perone G, Giannotti C, Candiani A, Latronico N. Antibiotic prophylaxis of early onset pneumonia in critically ill comatose patients. A randomized study. Intensive Care Med. 2005; 31(4):510-6.

S238. Aerdts SJ, van Dalen R, Clasener HA, Festen J, van Lier HJ, Vollaard EJ: Antibiotic prophylaxis of respiratory tract infection in mechanically ventilated patients. A prospective, blinded, randomized trial of the effect of a novel regimen. Chest. 1991;100:783-791

S239. Bion JF, Badger I, Crosby HA, Hutchings P, Kong KL, Baker J, Hutton P, McMaster P, Buckels JA, Elliott TSJ: Selective decontamination of the digestive tract reduces gram-negative pulmonary colonization but not systemic endotoxemia in patients undergoing elective liver transplantation. Crit Care Med. 1994;22:40-49

S240. Blair P, Rowlands BJ, Lowry K, Webb H, Armstrong P, Smilie J Selective decontamination of the digestive tract: a stratified, randomized, prospective study in a mixed intensive care unit. Surgery 1991;110:303-309

S241. Blaise M, Pateron D, Trinchet JC, Levacher S, Beaugrand M, Pourriat JL. Systemic antibiotic therapy prevents bacterial infection in cirrhotic patients with gastrointestinal hemorrhage. Hepatology. 1994;20(1):34-8.

S242. Bouza E, Granda MJ, Hortal J, Barrio JM, Cercenado E, Muñoz P. Pre-emptive broad-spectrum treatment for ventilator-associated pneumonia in high-risk patients. *Intensive Care Med* 2013;39(9):1547-55.

S243. Camus C, Bellissant E, Sebille V, Perrotin D, Garo B, Legras A, Renault A, Le Corre P, Donnio PY, Gacouin A: Prevention of acquired infections in intubated patients with the combination of two decontamination regimens. *Crit Care Med* 2005, 33:307-314.

S244. Cerra FB, Maddaus MA, Dunn DL, Wells CL, Konstantinides NN, Lehmann SL, Mann HJ. Selective gut decontamination reduces nosocomial infections and length of stay but not mortality or organ failure in surgical intensive care unit patients. Arch Surg 1992;127:163-167.

S245. Cockerill FR, 3rd, Muller SR, Anhalt JP, et al. Prevention of infection in critically ill patients by selective decontamination of the digestive tract. *Ann Intern Med* 1992;117:545-53.

S246. de La Cal MA, Cerdá E, Garcıa-Hierro P, Van Saene HK. G ómez-Santos D, Negro E & Lorente JA. Survival benefit in critically ill burned patients receiving selective decontamination of the digestive tract: a randomized, placebocontrolled, double-blind trial. Ann Surg. 2005;241:424-30.

S247. Ferrer M, Torres A, Gonzalez J, Puig de la Bellacasa J, el-Ebiary M, Roca M, Gatell JM, Rodriguez-Roisin R: Utility of selective digestive decontamination in mechanically ventilated patients. Ann Intern Med. 1994;120:389-395

S248. Flaherty J, Nathan C, Kabins SA, Weinstein RA. Pilot trial of selective decontamination for prevention of bacterial infection in an intensive care unit. J Infect Dis 1990;162:1393-1397.

S249. Garbino J, Pichard C, Pichna P, Pittet D, Lew D, Romand J (2004) Impact of enteral versus parenteral nutrition on the incidence of fungal infections: a retrospective study in ICU patients on mechanical ventilation with selective digestive decontamination. Clinical Nutrition 2004;23;705-710.

S250. Gaussorgues P, Salord M, Sirodot S, Tigaud S, Cagnin S, Gerard M, Robert D. Efficiency of selective decontamination of the digestive tract on the occurrence of nosocomial bacteremia in patients on mechanical ventilation receiving betamimetic therapy. Réan Soins Intens Méd Urg 1991;7:169-174.

S251. Georges B, Mazerolles M, Decun J-F, Rouge P, Pomies S, Cougot P Décontamination digestive sélective résultats d'une étude chez le polytraumatisé. *Réanimation Soins Intensifs Médecin d'Urgence* 1994;3:621-627

S252. Hammond JM, Potgieter PD. Neurologic disease requiring long-term ventilation: the role of selective decontamination of the digestive tract in preventing nosocomial infection. *Chest*. 1993; 104(2):547-51.

S253. Hammond JM, Potgieter PD, Saunders LG. Selective decontamination of the digestive tract in multiple trauma patients-Is there a role? Results of a prospective, double-blind, randomized trial. Crit Care Med. 1994;22(1):33-9.

S254. Hellinger WC, Yao JD, Alvarez S, Blair JE, Cawley JJ, Paya CV, O'Brien PC. A randomized, prospective, double-blinded evaluation of selective bowel decontamination in liver transplantation. Transplantation 2002;73:1904-9.

S255. Jacobs S, Foweraker JE, Roberts SE: Effectiveness of selective decontamination of the digestive tract (SDD) in an ICU with a policy encouraging a low gastric pH. Clin Intensive Med. 1992;3:52-58

S256. Karvouniaris M, Makris D, Zygoulis P, Triantaris A, Xitsas S, Mantzarlis K, Petinaki E, Zakynthinos E. Nebulised colistin for ventilator-associated pneumonia prevention. Eur Resp J. 2015:46:1544-1547.

S257. Kerver AJ, Rommes JH, Mevissen-Verhage EA, et al Prevention of colonization and infection in critically ill patients: a prospective randomized study. Crit Care Med 1988;16:1087-1093.

S258. Korinek AM, Laisne MJ, Nicolas MH, Raskine L, Deroin V, Sanson-lepors MJ: Selective decontamination of the digestive tract in neurosurgical intensive care unit patients: a double-blind, randomized, placebo-controlled study. Crit Care Med. 1993;21:1466-73.

S259. Laggner AN, Tryba M, Georgopoulos A, Lenz K, Grimm G, Graninger W, Schneeweiss B, Druml W: Oropharyngeal decontamination with gentamicin for long-term ventilated patients on stress ulcer prophylaxis with sucralfate? *Wien Klin Wochenschr* 1994, 106:15-19.

S260. Langlois-Karaga A, Bues-Charbit M, Davignon A, Albanese J, Durbec O, Martin C, Morati N, Balansard G. Selective digestive decontamination in multiple trauma patients: cost and efficacy. *Pharmacy World and Science*. 1995;17(1):12-6.

S261. Palomar M, Alvarez-Lerma F, Jorda R, Bermejo B, Catalan Study Group of Nosocomial Pneumonia Prevention: Prevention of nosocomial infection in mechanically ventilated patients: selective digestive decontamination versus sucralfate. Clin Intens Care. 1997;8:228-235

S262. Pneumatikos I, Koulouras V, Nathanail C, Goe D, Nakos G: Selective decontamination of subglottic area in mechanically ventilated patients with multiple trauma. *Intensive Care Med*. 2002;28:432-437

S263. Quinio B, Albanese J, Bues-Charbit M, Viviand X, Martin C; Selective decontamination of the digestive tract in multiple trauma patients. A prospective double-blind, randomized, placebo-controlled study. Chest 1996;109:765-772

S264. Rimola A, Bory F, Teres J, Perez-Ayuso RM, Arroyo V, Rodes J. Oral, nonabsorbable antibiotics prevent infection in cirrhotics with gastrointestinal hemorrhage. *Hepatol*. 1985;5(3):463-7.

S265. Rocha LA, Martin MJ, Pita S, Paz J, Seco C, Margusino L, Villanueva R, Duran MT: Prevention of nosocomial infection in critically ill patients by selective decontamination of the digestive tract. A randomized, double blind, placebo-controlled study. Intensive Care Med. 1992;18:398-404

S266. Rodríguez-Roldán JM, Altuna-Cuesta A, López A, Carrillo A, Garcia J, León J, Martínez-Pellús AJ: Prevention of nosocomial lung infection in ventilated patients: use of an antimicrobial pharyngeal nonabsorbable paste. Crit Care Med. 1990;18:1239-42

S267. Rolando N, Gimson A, Wade J, Philpott-Howard J, Casewell M, Williams R: Prospective controlled trial of selective parenteral and enteral antimicrobial regimen in fulminant liver failure. Hepatol. 1993;17:196-201

S268. Rolando N, Wade JJ, Stangou A, Gimson AE, Wendon J, Philpott-Howard J, Williams R. Prospective study comparing the efficacy of prophylactic parenteral antimicrobials, with or without enteral decontamination, in patients with acute liver failure. Liver transplantation and surgery. 1996; 2:8-13

S269. Sanchez-Garcia M, Cambronero JA, Lopez-Diaz J, et al. Effectiveness and cost of selective decontamination of the digestive tract in critically ill intubated patients. A randomized, double-blind, placebo-controlled multicenter trial. *Am Rev Respir Dis* 1998; 158:908-16.

S270. Sirvent JM, Torres A, El-Ebiary M, Castro P, de Batlle J, Bonet A. Protective effect of intravenously administered cefuroxime against nosocomial pneumonia in patients with structural coma. *Am J Respir Crit Care Med* 1997;155:1729-1734

S271. Smith SD, Jackson RJ, Hannakan CJ, Wadowsky RM, Tzakis AG, Rowe MI; Selective decontamination in pediatric liver transplants. Transplantation 1993;55:1306-1308

S272. Stoutenbeek CP, van Saene HKF, Little RA, Whitehead A: The effect of selective decontamination of the digestive tract on mortality in multiple trauma patients: a multicenter randomized controlled trial. Intensive Care Med. 2007;33:261-270

S273. Ulrich C, Harinck-deWeerd JE, Bakker NC, et al Selective decontamination of the digestive tract with norfloxacin in the prevention of ICU-acquired infections: A prospective randomized study. Intensive Care Med 1989;15:424-431.

S274. Unertl K, Ruckdeschel G, Selbmann HK, et al; Prevention of colonization and respiratory infections in long-term ventilated patients by local antimicrobial prophylaxis. Intensive Care Med 1987;13:106-113

S275. Van Delden C, Köhler T, Brunner-Ferber F, François B, Carlet J, Pechère JC. Azithromycin to prevent Pseudomonas aeruginosa ventilator-associated pneumonia by inhibition of quorum sensing: a randomized controlled trial. Intensive Care Med. 2012 Jul 1;38(7):1118-25.

S276. Verwaest C, Verhaegen J, Ferdinande P, Schetz M, Van den Berghe G, Verbist L, Lauwers P: Randomized, controlled trial of selective digestive decontamination in 600 mechanically ventilated patients in a multidisciplinary intensive care unit. Crit Care Med. 1997;25:63-71

S277. Wiener J, Itokazu G, Nathan C, Kabins SA, Weinstein RA: A randomized, double-blind, placebo-controlled trial of selective digestive decontamination in a medical-surgical intensive care unit. Clin Infect Dis. 1995;20:861-867

S278. Ables AZ, Blumer NA, Valainis GT, Godenick MT, Kajdasz DK, Palesch YY. Fluconazole Prophylaxis of Severe Candida Infection in Trauma and Postsurgical Patients: A Prospective, Double-Blind, Randomized, Placebo-Controlled Trial. Infect Dis Clin Practice 2000;9(4):169-175.

S279. Eggimann P, Francioli P, Bille J, Schneider R, Wu MM, Chapuis G, Chiolero R, Pannatier A, Schilling J, Geroulanos S, et al (1999) Fluconazole prophylaxis prevents intra-abdominal candidiasis in high-risk surgical patients. Crit Care Med 27:1066–1072.

S280. Giglio M, Caggiano G, Dalfino L, Brienza N, Alicino I, Sgobio A, Favale A, Puntillo F. Oral nystatin prophylaxis in surgical/trauma ICU patients: a randomised clinical trial. Crit Care 2012;16(2):R57.

S281. Jacobs S, Price Evans DA, Tariq M, Al Omar NF (2003) Fluconazole improves survival in septic shock: a randomized double-blind prospective study. Crit Care Med 31:1938–1946.

S282. Lumbreras C, Cuervas-Mons V, Jara P, Del Palacio A, Turrion VS, Barrios C, Paya C V (1996) Randomized trial of fluconazole versus nystatin for the prophylaxis of *Candida* infection following liver transplantation. J Infect Dis 174(3); 583-588.

S283. Normand S, François B, Dardé ML, Bouteille B, Bonnivard M, Preux PM, Gastinne H, Vignon P. Oral nystatin prophylaxis of *Candida* spp. colonization in ventilated critically ill patients. Intensive Care Med 2005;31:1508–1513.

S284. Ostrosky-Zeichner L, Shoham S, Vazquez J, Reboli A, Betts R, Barron MA, Pappas P G. MSG-01: a randomized, double-blind, placebo-controlled trial of caspofungin prophylaxis followed by pre-emptive therapy for invasive candidiasis in high-risk adults in the critical care setting. Clin Infect Dis 2014;58(9):1219-1226.

S285. Savino JA, Agarwal N, Wry P, Policastro A, Cerabona T, Austria L. Routine prophylactic antifungal agents (clotrimazole, ketoconazole, and nystatin) in nontransplant nonburned critically ill surgical and trauma patients. J Trauma 1994;36:20–26.

S286. Schuster MG, Edwards JE, Sobel JD, Darouiche RO, Karchmer AW, Hadley S, Rex JH (2008) Empirical fluconazole versus placebo for intensive care unit patients: a randomized trial. Ann Intern Med 149(2):83-90.

S287. Verhaegen J: Randomized study of selective digestive decontamination on colonization and prevention of infection in mechanically ventilated patients in the ICU. 1992. Doctor in Medical Sciences – thesis, University Hospital, Leuven, Belgium.

Fig S1. (p 45) Search method, screening criteria and resulting classification of eligible studies and subsequent decant of component groups. The four numbered arrows are as follows;

(1) An electronic search for systematic reviews or meta-analysis (SR/MA) containing potentially eligible studies using search terms; “ventilator associated pneumonia”, “mechanical ventilation”, “intensive care unit”, each combined with either “meta-analysis” or “systematic review” up to December 2018;

(2) The systematic reviews were then searched for studies of patient populations requiring prolonged (> 24 hours) ICU admission

(3) The studies were triaged from the systematic reviews into one of five categories; studies in which there was no intervention (observational studies), studies of various non-decontamination methods such as methods delivered either via the gastric route, the airway route or via the oral care route, studies of anti-septic methods, studies with a TAP (in any formulation) based intervention, and studies of antifungal prophylaxis as a single agent.

(4) All studies were reviewed for potentially eligible studies and screened against inclusion and exclusion criteria. Any duplicate or ineligible studies were removed and

(5) Studies identified outside of systematic reviews were included;

(6) The component groups were decanted from each study being control (rectangles), intervention (ovals) and observation (diamond) groups.

Note; the total numbers do not tally as some systematic reviews provided studies in more than one category and some studies provided groups in more than one category and some studies have unequal numbers of control and interventions groups.

TAP = Topical antibiotic prophylaxis

Figure 1: Flow chart of literature search and study and group decant

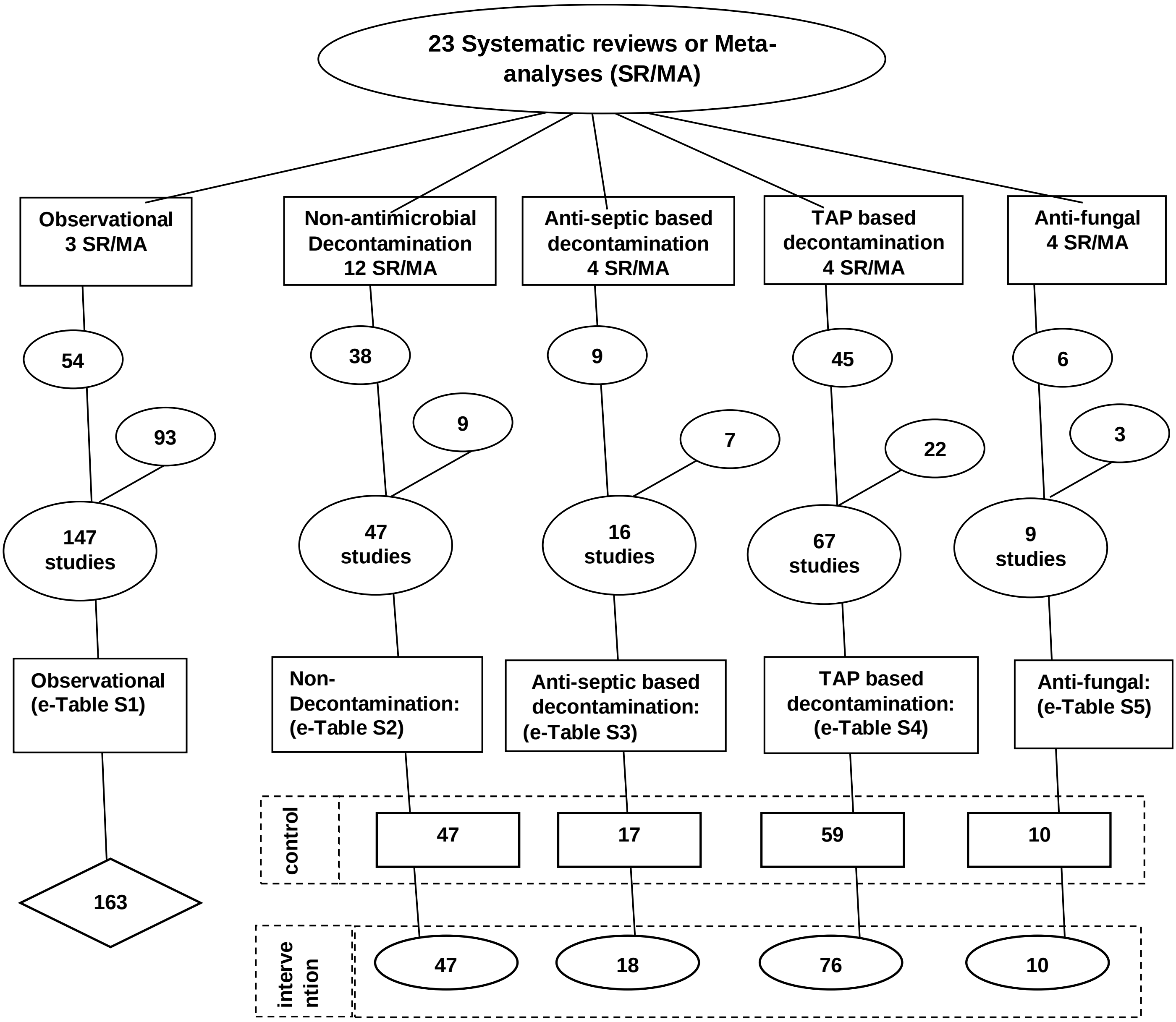

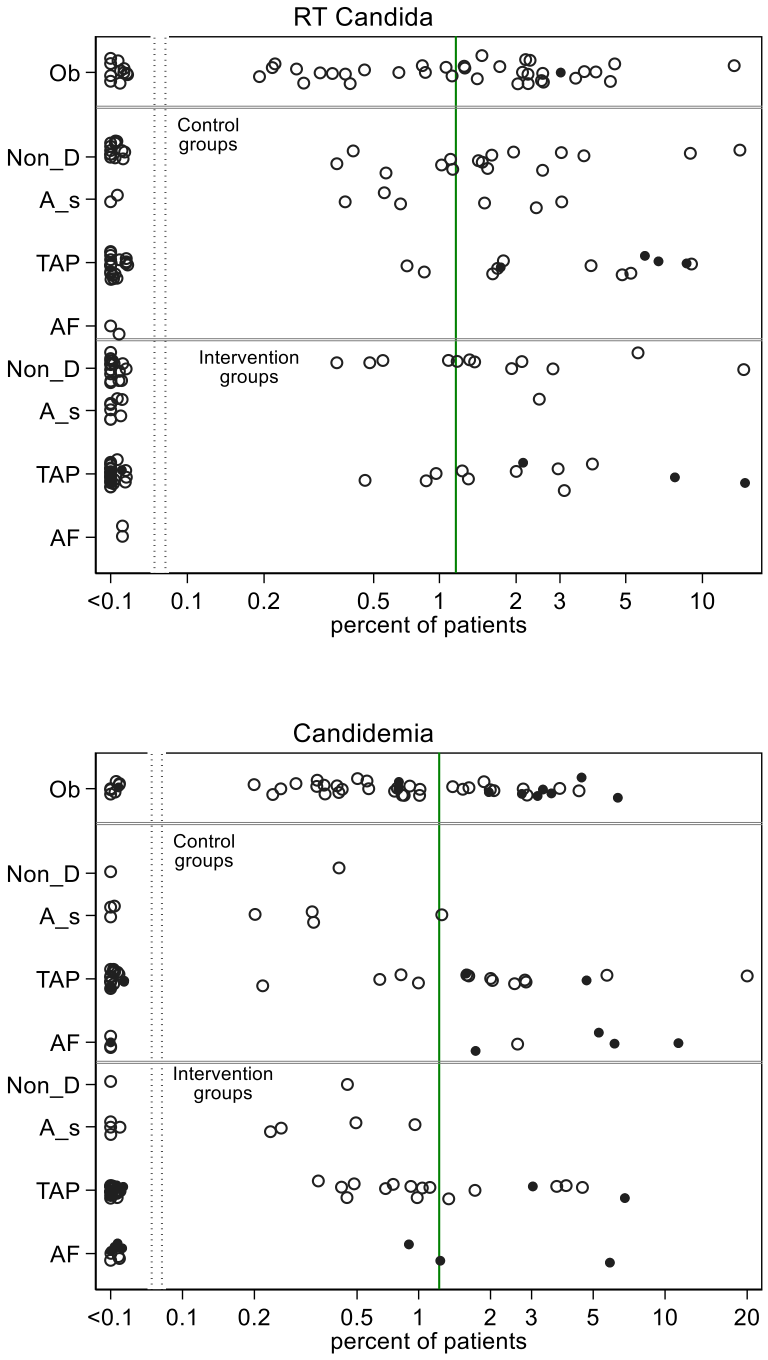

Figure s2a & b Scatter plots (logit scale) of RT Candida incidence (top) and Candidemia (bottom) in component (control and intervention) groups of various methods of infection prevention in the ICU (all studies). The benchmark incidence in each plot is the summary mean derived from the observation studies (central vertical line). The groups wide presence of candidemia risk factors (CRF) is identified by solid symbols versus not (open).
non-D is non-decontamination, A_s is anti-septic, TAP is topical antibiotic prophylaxis and AF is anti-fungal

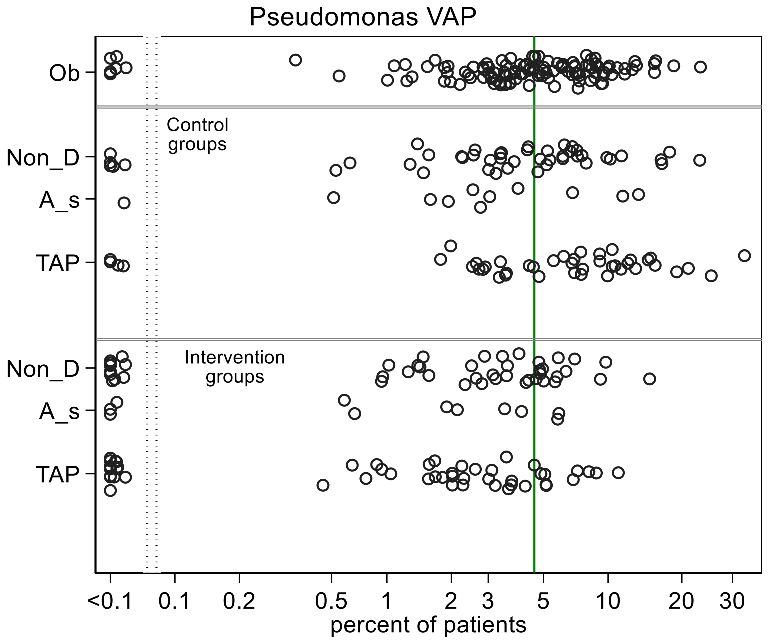

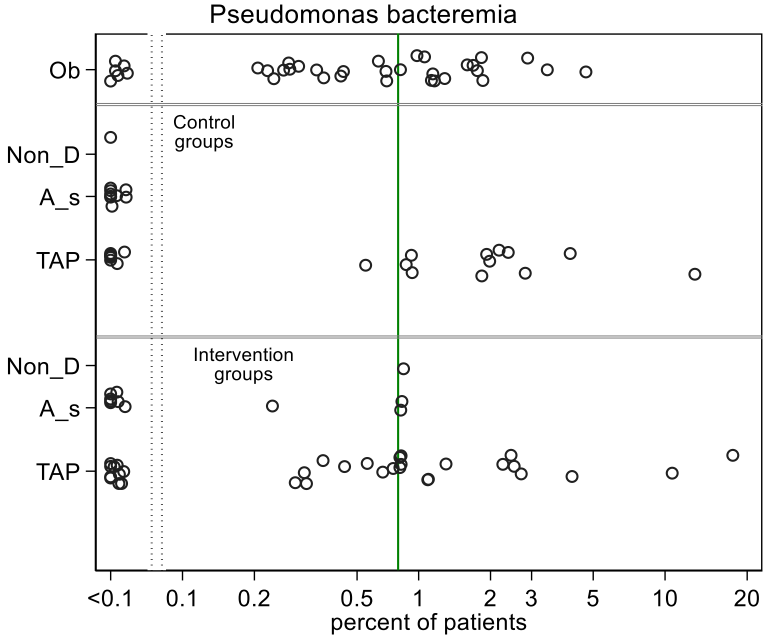

Figure s3a & b Scatter plots (logit scale) of Pseudomonas VAP incidence (top) and Pseudomonas bacteremia (bottom) in (control and intervention) component groups of various methods of infection prevention in the ICU (all studies). The benchmark incidence in each plot is the summary mean derived from the observation studies (central vertical line). non-D is non-decontamination, A_s is anti-septic, TAP is topical antibiotic prophylaxis and AF is anti-fungal

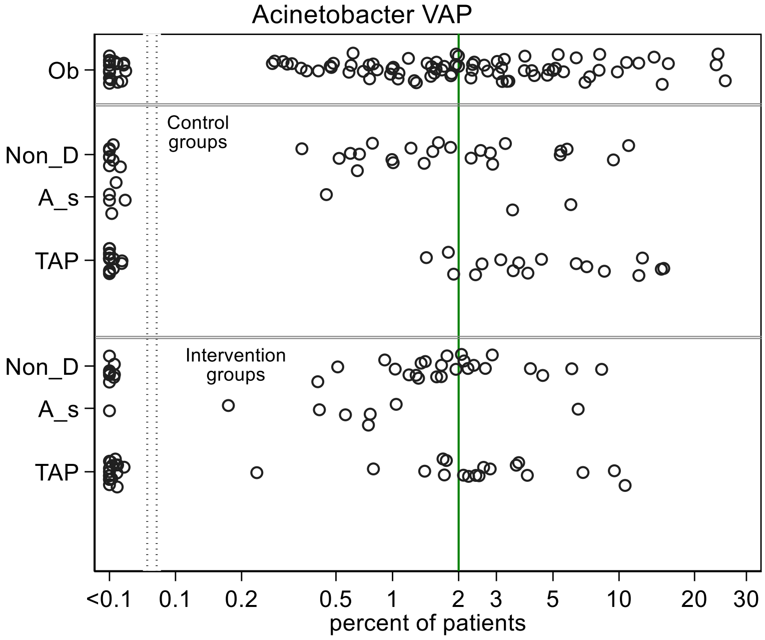

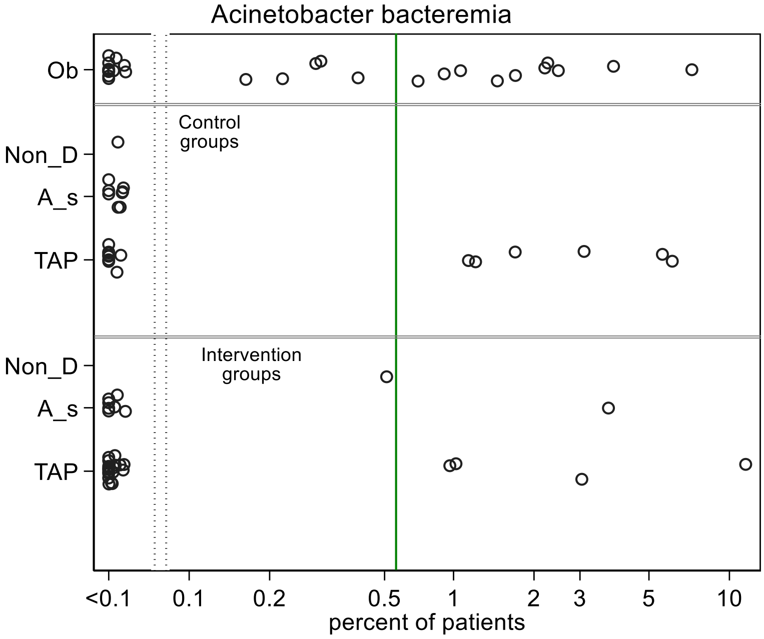

Figure s4a & b Scatter plots (logit scale) of Acinetobacter VAP incidence (top) and Acinetobacter bacteremia (bottom) in (control and intervention) component groups of various methods of infection prevention in the ICU (all studies). The benchmark incidence in each plot is the summary mean derived from the observation studies (central vertical line). non-D is non-decontamination, A_s is anti-septic, TAP is topical antibiotic prophylaxis and AF is anti-fungal

Figure s5a & b. GSEM model 1 (top) & 2 (bottom) – Pseudomonas – all studies (see Table S6 for abbreviations)

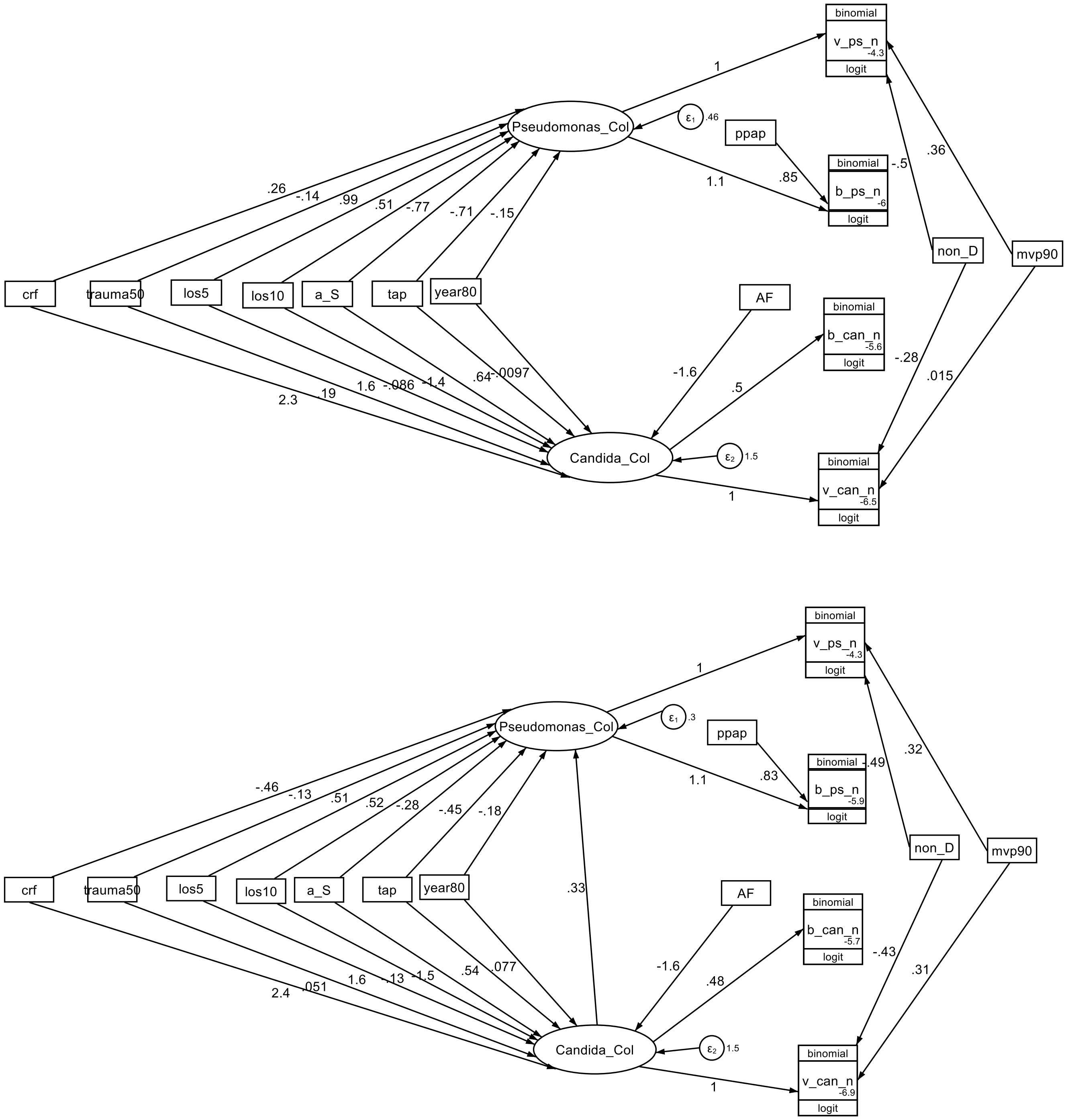

Figure s6 a & b. GSEM model 3 (top) & 4 (bottom) – Pseudomonas excluding studies with ICU-LOS<5 days (see Table S6 for abbreviations)

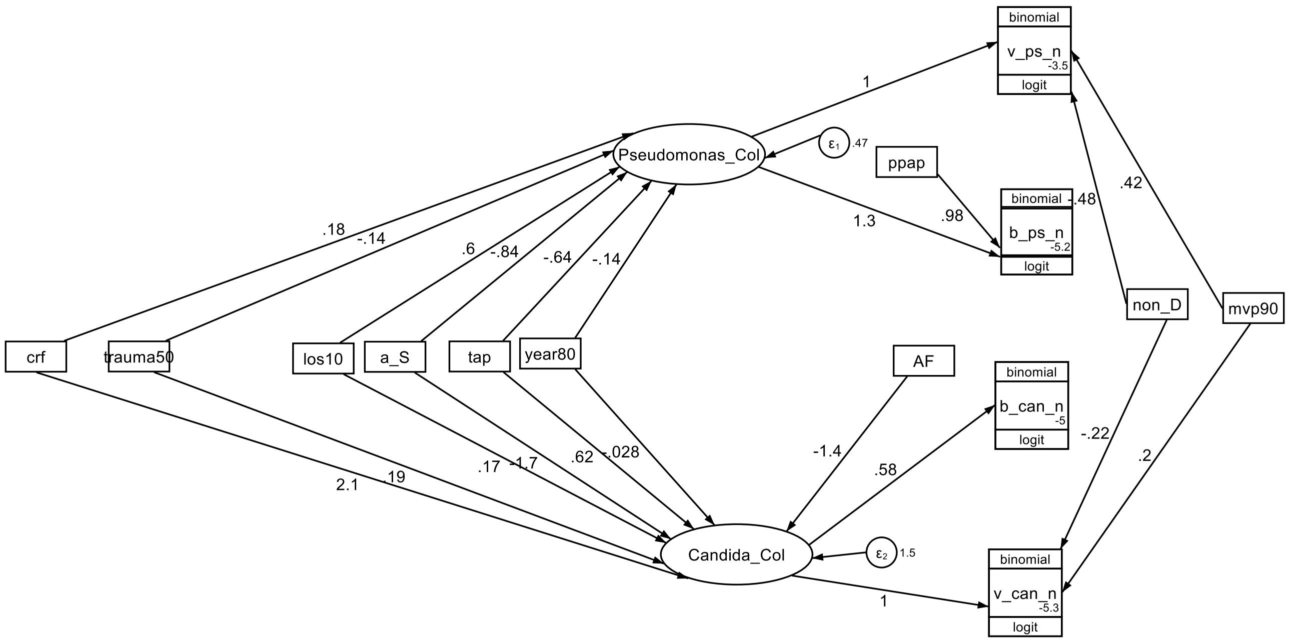

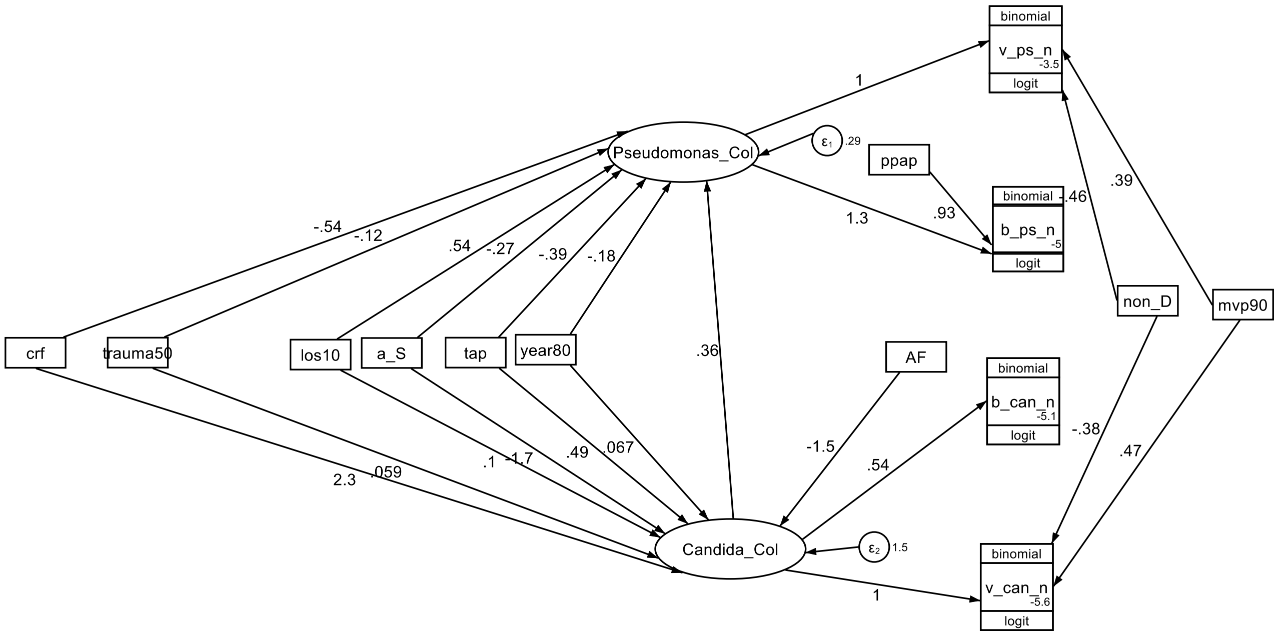

Figure s7 a & b. GSEM model 5 (top) & 6 (bottom) Acinetobacter – all studies (see Table S7 for abbreviations)

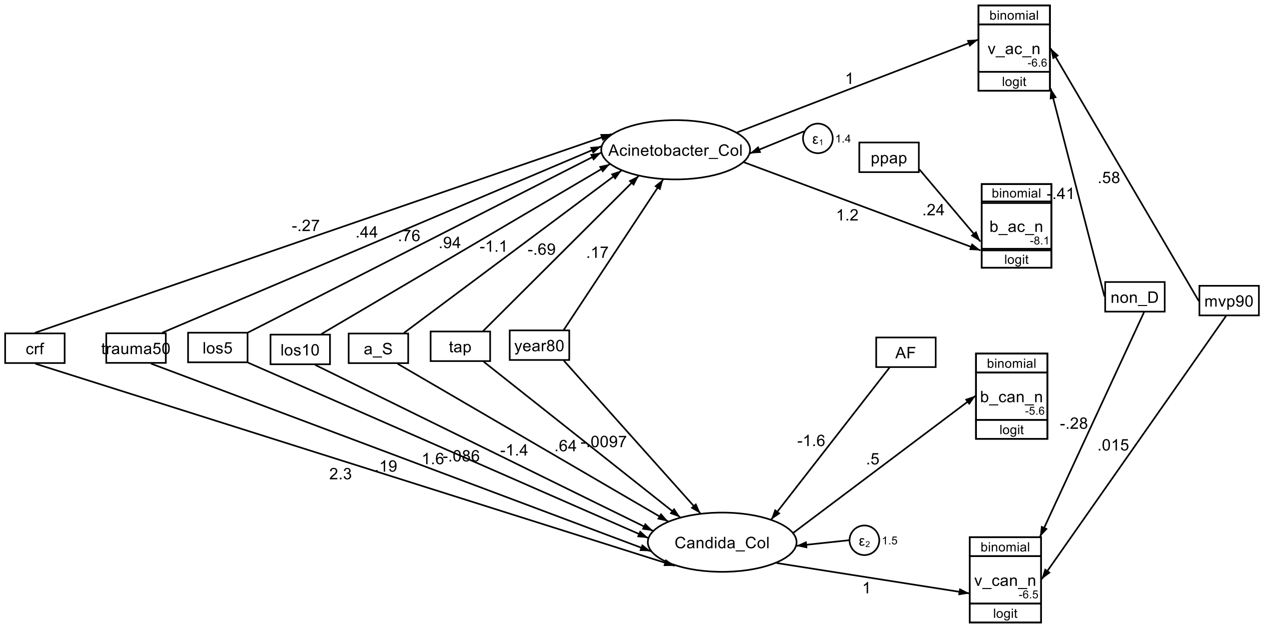

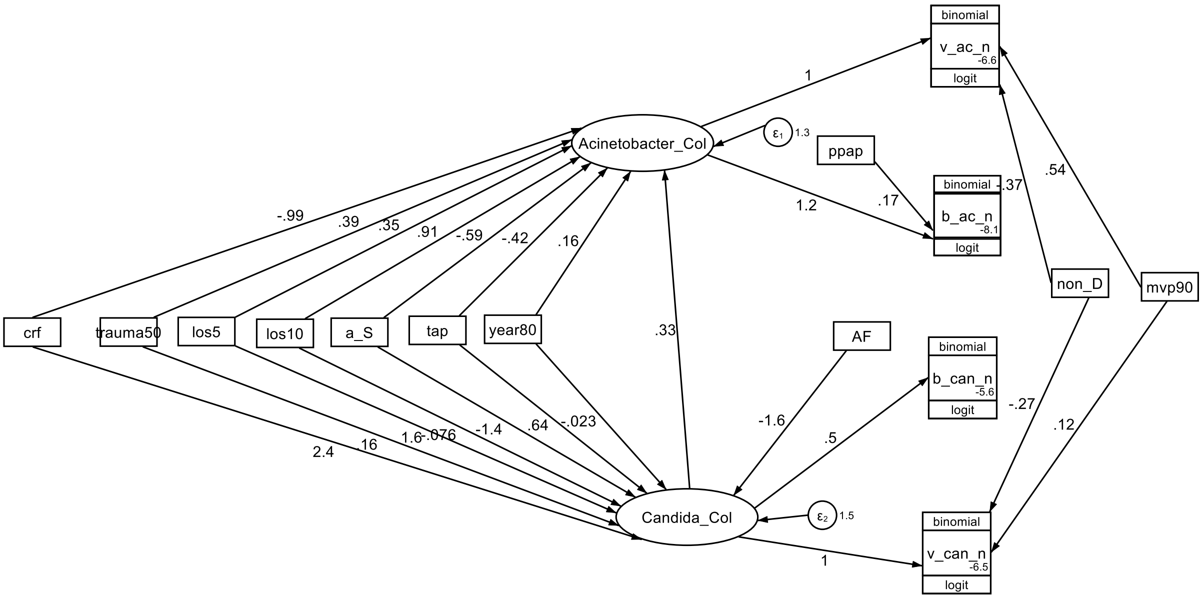

Figure s8 a & b. GSEM model 7(top) & 8 (bottom) – Acinetobacter excluding studies with ICU-LOS<5 days (see Table S7 for abbreviations)

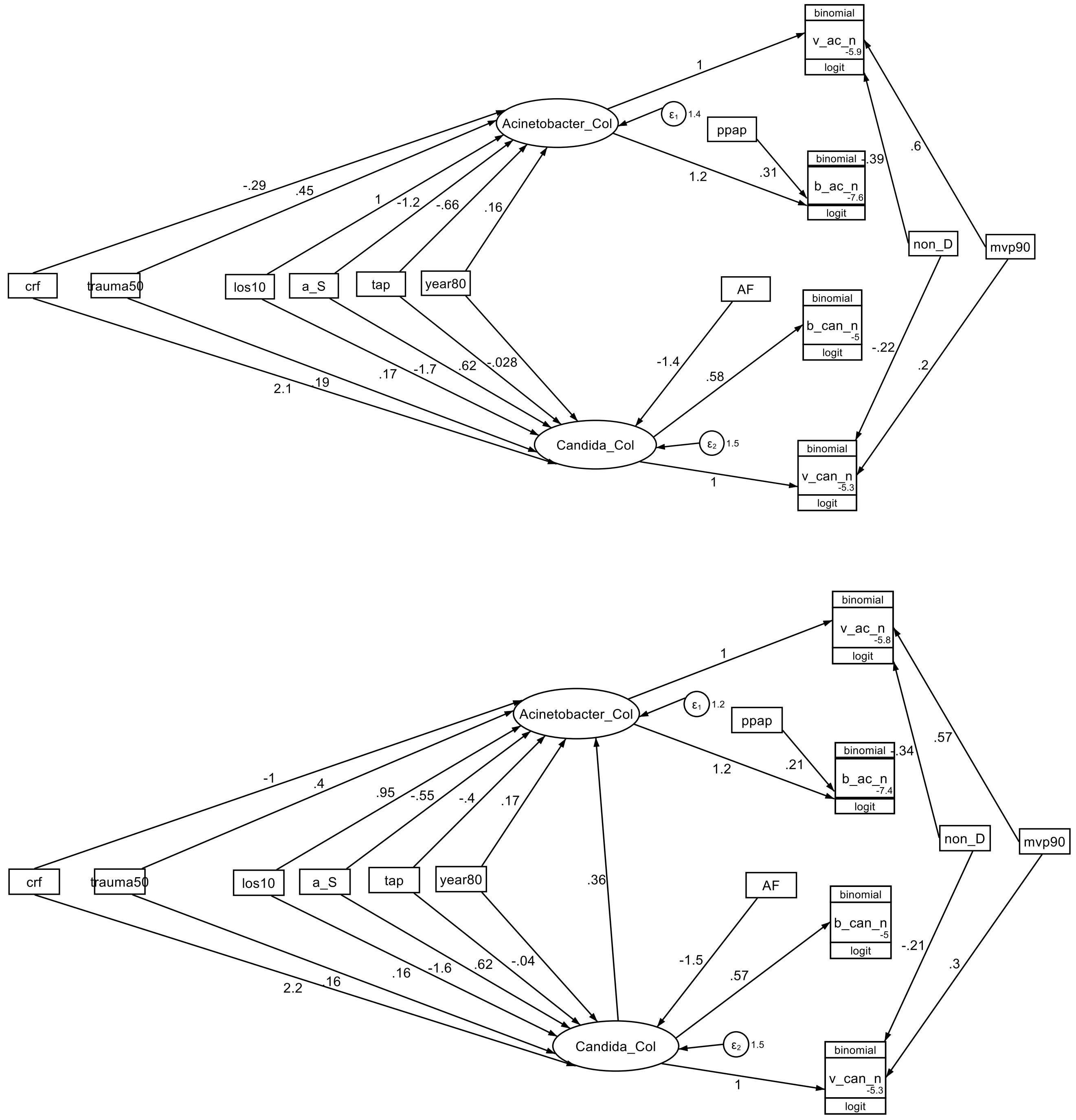

Supplement: Supplementary file 1 [file jof-06-00252-s001.pdf]
